# Mechanistic Divergence in the Hydrogenative Synthesis of Furans and Butenolides: Ruthenium Carbenes Formed by *gem*‐Hydrogenation or through Carbophilic Activation of Alkynes

**DOI:** 10.1002/anie.201912161

**Published:** 2019-11-06

**Authors:** Sebastian Peil, Alois Fürstner

**Affiliations:** ^1^ Max-Planck-Institut für Kohlenforschung 45470 Mülheim/Ruhr Germany

**Keywords:** Bürgi–Dunitz angle, carbophilic activation, furans, hydrogenation, metal carbenes, ruthenium

## Abstract

Enynes with a tethered carbonyl substituent are converted into substituted furan derivatives upon hydrogenation using [Cp*RuCl]_4_ as the catalyst. Paradoxically, this transformation can occur along two distinct pathways, each of which proceeds via discrete pianostool ruthenium carbenes. In the first case, hydrogenation and carbene formation are synchronized (“gem‐hydrogenation”), whereas the second pathway comprises carbene formation by carbophilic activation of the triple bond, followed by hydrogenative catalyst recycling. Representative carbene intermediates of either route were characterized by X‐ray crystallography; the structural data prove that the attack of the carbonyl group on the electrophilic carbene center follows a Bürgi–Dunitz trajectory.

## Graphical abstract

**One way or the other**: Enynes bearing a tethered carbonyl group are transformed into furans upon catalytic hydrogenation using a ruthenium catalyst. Strikingly though, the reaction can follow two distinct pathways, each of which involves discrete metal carbene intermediates. They differ in that the hydrogenation and carbene formation are either synchronized (*gem*‐hydrogenation) or fully uncoupled.

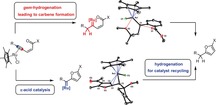

After a century of intense research by the scientific community on catalytic hydrogenation, our group has recently been able to identify an entirely new reactivity mode. Specifically, it was shown that alkynes can undergo *gem*‐hydrogenation, a reaction in which both H‐atoms of H_2_ are delivered to one and the same acetylenic C‐atom while the adjacent position is concomitantly transformed into a discrete metal carbene.^1^, ^2^ At the current stage of development, [Cp*RuCl]_4_ is the precatalyst of choice; moreover, heteroatom substituents in vicinity of the triple bond are often necessary to render the reaction efficient. Detailed spectroscopic and computational data indicate that the resulting pianostool ruthenium complexes basically exhibit a Fischer‐carbene character with a certain overtone reminiscent of Grubbs‐type catalysts.^3^ This view is corroborated by the fact that they participate in either intramolecular cyclopropanation or metathesis reactions, depending on the chosen substrate.^4^

For their largely electrophilic nature, such complexes should be able to participate in various other catalytic transformations too. If this is the case, *gem*‐hydrogenation might eventually evolve into an attractive alternative to diazoalkane decomposition, which is arguably the most common gateway to highly reactive late‐transition metal carbenes.^5^, ^6^ The foray along these lines outlined below was inspired by a recent publication describing an innovative entry into highly substituted furan derivatives (Scheme 1).^7^ Specifically, diazo compounds **A** were shown to react with catalytic [CpRu(MeCN)_3_]PF_6_ to generate transient cationic ruthenium carbenes **B**, which get trapped by the tethered ester group to give the corresponding heterocycle **C**. We reasoned that this type of transformation might be emulated by *gem*‐hydrogenation of enyne **D**, in which the propargylic ‐OR substituent directs carbene formation to the distal acetylenic site.^1^, ^2^ Akin to **B**, the resulting intermediate **E** might furnish furan **F**, even though **E** is a neutral rather than cationic entity.

**Scheme 1:**
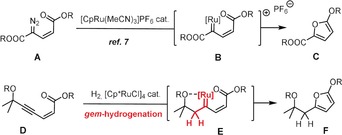
Conceived furan formation by alkyne *gem*‐hydrogenation, emulating known diazo‐decomposition chemistry; Cp=cyclopentadienyl; Cp*=pentamethylcyclopentadienyl.

In line with our expectations, product **2** was formed in almost quantitative yield (≥95 %) upon stirring of a solution of **1** and [Cp*RuCl]_4_ (2 mol %) in CH_2_Cl_2_ under an atmosphere of H_2_ (1 bar) for 3 h at ambient temperature (Scheme 2). Treatment of the crude material with silica released the corresponding butenolide **3**.^8^ Alternatively, **2** can be alkylated with allyl iodide to give product **4**.^9^ In other cases such as **5**–**7**, the furan itself was sufficiently stable for isolation. In line with our previous investigations,^2^, ^3^, ^4^ different propargylic substituents (for example, ‐OR, ‐OSiR_3_, ‐OMOM) were found to instigate *gem*‐hydrogenation. Gratifyingly though, even substrates devoid of such directing groups led to the formation of butenolides **10** and **11**;^10^ in these cases, the ester itself might serve as a directing group, fostering regioselective carbene formation by *gem*‐hydrogenation.2a This effect is not always sufficient, however, as illustrated by the formation of alkene **12** through *trans*‐hydrogenation of the corresponding alkyne substrate.^11^ This outcome is not overly surprising since *trans*‐hydrogenation has previously been shown to be a facile process downstream of an initial *gem*‐hydrogenation event (even though **12** could very well originate from a concerted pathway).^1^, ^2^, ^12^

**Scheme 2:**
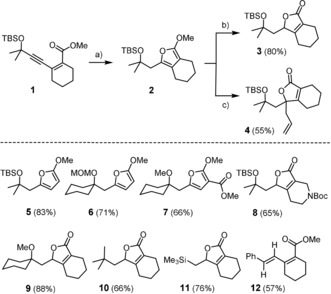
a) [Cp*RuCl]_4_ (2 mol %), H_2_ (1 atm), CH_2_Cl_2_, RT; b) SiO_2_; c) allyl iodide, NaI, THF, 100 °C; Boc=*tert*‐butoxycarbonyl; MOM=methoxymethyl; TBS=*tert*‐butyldimethylsilyl.

Although these results are fully consistent with the formation of pianostool ruthenium carbenes by *gem*‐hydrogenation in the first place, we sought to confirm this mechanistic interpretation. To this end, we resorted to substrate **13**, which is expected to undergo regular *gem*‐hydrogenation but should be resilient to cyclization because heterocycle formation comes at the prize of dearomatization of the phenyl ring (Scheme 3). Indeed, the reaction stopped at the stage of the pianostool ruthenium carbene complex **14**. The use of *para*‐hydrogen as the reagent leads to a massive amplification of the ^1^H NMR signal of the methylene group flanking the carbene center as a result of the PHIP effect (PHIP=*p*‐hydrogen induced polarization).^13^, ^14^ This spectroscopic signature provides unambiguous proof that these two H‐atoms originate from the same H_2_ molecule by pairwise delivery, a characteristic trait of *gem*‐hydrogenation.^2^

**Scheme 3:**
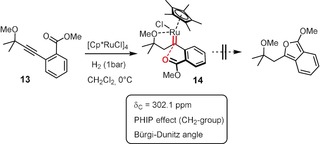
Interrupted process: *gem*‐Hydrogenation without subsequent heterocycle formation.

The structure of this sensitive complex in the solid state is highly informative (Figure 1). It suggests that the directing effect of the methyl ether substituent emanates from its interaction with the Lewis‐acidic Ru center at a distance of 2.18 Å; the actual C1−Ru1 carbene bond length (1.883(2) Å) is in the expected range.^2^ The arguably most remarkable structural feature, however, is the orientation of the carbonyl oxygen atom O1 relative to the carbene center C1: the O1−C1 distance (2.983(4) Å) is well below the sum of the van‐der‐Waals radii of these atoms (ca. 3.22 Å) and the Ru1−C1−O1 angle of 116.0° shows that the ester carbonyl approaches the trigonal carbene center along a Bürgi–Dunitz trajectory.^15^, ^16^ In view of this ideal geometric predisposition for an outer‐sphere attack, it is reasonable to believe that ring closure stalls because of the unfavorable thermodynamic rendering of this model compound, since loss of aromaticity of the benzene ring would not be compensated by the enthalpic gain of an emerging isobenzofuran. The Bürgi–Dunitz angle is well recognized as a fundamental principle of (dynamic) stereochemistry originating from the particular shape and occupancy of the relevant frontier orbitals.^17^ Yet, complex **14** seems to be the first case of an electrophilic transition metal carbene and its nucleophilic reaction partner, for which this prominent effect has been explicitly recognized as a structure‐determining element manifest in the crystallographic data.^18^

**Figure 1:**
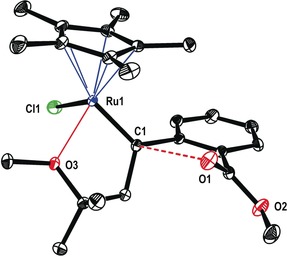
Structure of carbene complex **14** in the solid state. The dotted line indicates that the ester carbonyl oxygen atom O1 adopts a Bürgi–Dunitz trajectory in its approach to the electrophilic carbene site C1; the O1−C1 distance is well below the sum of the van‐der‐Waals radii.

Next, we attempted to extend the novel hydrogenative furan formation to substrates carrying nucleophiles other than an ester group. The readily available diketone derivative **15** gave the expected product **16** but required a reaction temperature of 70 °C (Scheme 4). Surprisingly, small amounts of ketone **18** were also isolated in one run from the crude mixture, which seems to indicate incidental oxidation of an intermediate of type **G** featuring the carbene site proximal to the directing ‐OMe substituent. Such a regiochemical course violates the mechanistic rationale outlined above for the ester series and is inconsistent with all other available information on alkyne *gem*‐hydrogenation,^1^, ^2^, ^3^, ^4^ not least with the X‐ray structure shown in Figure 1. Formation of **G** might be explained, however, by assuming that the π‐acidic metal fragment activates the substrate to the extent that attack of the ketone onto the triple bond is faster than binding of H_2_, as necessary for *gem*‐hydrogenation to occur. This type of mechanism has ample precedent in the literature for many different carbophilic catalysts,^19^ although rigorous proof for the intervention of discrete carbenes is exceedingly rare.^20^

**Scheme 4:**
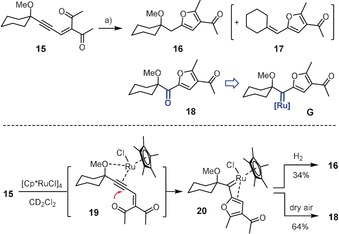
Extension to the ketone series is accompanied by a switch in mechanism: a) [Cp*RuCl]_4_ (5 mol %), H_2_ (1 atm), 1,2‐dichloroethane, 70 °C, 57 % (**16**), ≤5 % (**17**), ≤10 % (**18**).

To test this hypothesis, enyne **15** was reacted with stoichiometric amounts of [Cp*RuCl]_4_ in the presence as well as in the absence of H_2_: either set‐up afforded the very same pianostool ruthenium carbene **20** in less than 30 min reaction time. The structure of this remarkable complex in the solid state shows that the ruthenium atom no longer interacts with the adjacent ‐OMe group (Figure 2); rather, it is tightly ligated to the electron rich “enol ether” site of the newly formed furan ring as manifested in the observed distances as well as in an elongated C2−C3 bond. The distinctive up‐field shift of the NMR signals of C2 (*δ*_C_=132.3 ppm), C3 (*δ*_C_=89.7 ppm), and the carbene center C1 (*δ*_C_=266.7 ppm) suggest that this bonding situation persists in CD_2_Cl_2_ solution.^21^ Despite the stabilizing interaction, **20** is a competent intermediate on the way to product and by‐product alike. It reacts with H_2_ at 70 °C to give furan **16**,^22^ whereas stirring of a solution in air afforded the furyl ketone **18** (Scheme 4).

**Figure 2:**
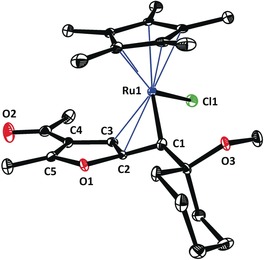
Structure of the furyl carbene **20** in the solid state; selected bond lengths (Å): Ru1−C1 1.890(3), C2−C3 1.399(4), C4−C5 1.363(4), Ru1−C2 2.217(2), Ru1−C3 2.330(2).

Since π‐bond activation by the carbophilic catalyst does not require assistance by a neighboring directing group,^23^ substrates of type **21** (R=alkyl, aryl) devoid of donor substituents at the propargylic position should react analogously (Scheme 5). As the formation of furans **23 a**,**b** and **24** shows, this is indeed the case as long as the substituent R shields the transient carbene center in **22**. If not, dimerization with formation of a tetrasubstituted alkene **26** becomes competitive. Thus, substrate **21 c** (R=Ph) gave a mixture of the monomeric furan **23 c** and olefin **26c** (R=Ph, *E*/*Z* ca. 1:1),^24^ whereas **21 d** (R=*n*‐Bu) afforded only traces of the monomeric furan derivative but furnished the product **26d** (R=*n*‐Bu) as the major product in the form of a single isomer. The *E*‐configuration of the central double bond could only be assigned by crystallographic means.^21^, ^25^ It is assumed that the triple bond of a second substrate inserts into the [Ru=C] bond of **22** in an enyne metathesis fashion to furnish a vinylcarbene **25**,^26^ which is then interrcepted by the adjacent ketone to close the second furan ring of the resulting dimer **26**.

**Scheme 5:**
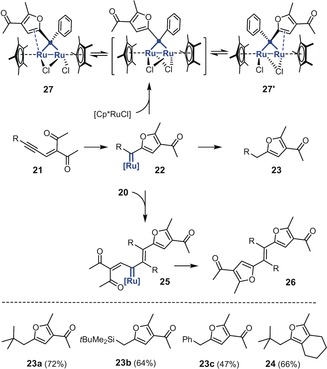
The fate of the pianostool ruthenium carbene formed by carbophilic activation.

Insufficient steric shielding of the carbene center can open yet another sidetrack. The NMR data of carbene **22a** (R=*t*Bu, *δ*_C_=285.3 ppm) correspond to those of **20** and its structure must hence be similar,^22^ but the ruthenium complex derived from enyne **21c** (R=Ph) and [Cp*RuCl]_4_ is strikingly different. Whereas **20** and **22a** (R=*t*Bu) are both rather sensitive burgundy‐red compounds, a very robust green solid material is formed that does not react with H_2_ even at 70 °C, is stable towards air and moisture for extended periods of time, and does not catalyze furan formation either; it is hence an off‐cycle product. The structure of this complex was determined by X‐ray diffraction. As shown in Figure 3, **27** incorporates a bridging rather than terminal carbene moiety; the dimer persists in solution as indicated by the markedly upfield‐shifted carbene resonance (*δ*_C_=189.9 ppm).^27^, ^28^ The distances between C1 and the two Ru atoms are uneven, as are the distances between the bridging chloride Cl2 and the metal atoms. Ru2 benefits from the same stabilizing interaction with the proximal enol site of the furan ring discussed above for **20**, whereas Ru1 carries an additional terminal chloride ligand.

**Figure 3:**
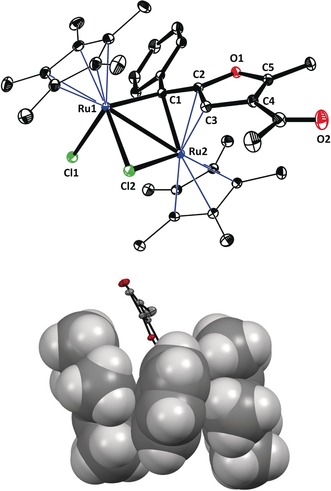
Top: Structure of complex **27** in the solid state; selected bond lengths (Å): Ru1−Ru1 2.822(3), Ru1−C1 2.099(1), Ru2−C1 2.118(1), Ru1−Cl1 2.448(3), Ru1−Cl2 2.456(3), Ru2−Cl2 2.393(4), Ru2−C2 2.208(1), Ru2−C3 2.284(1), C2−C3 1.416(2), C3−C4 1.452(2), C4−C5 1.365(2). Bottom: partial space‐filling representation (except for the furyl ring in the back), showing the immersion of the phenyl group between the lateral Cp* rings.

The static picture of the structure in the solid state, however, provides an incomplete description of this complex. Rather, the ^13^C NMR signals of the two Cp* rings show massive line broadening at ambient temperature; a reversible dyotropic process **27**/**27′** is the likely cause for the equilibration of the two different metal subunits,^29^ a dynamic behavior that can be frozen out by lowering the temperature to −50 °C. At the same time, the rotation of the phenyl substituent comes to a halt. Inspection of the space‐filling model of **27** shows that this phenyl ring is “sandwiched” between the lateral Cp* rings. Actually, it is plausible that the complex draws some of its remarkable stability from this peripheral interaction, even though this aspect needs further scrutiny. In any case, only slim substituents will be able to intercalate analogously. Only two related complexes are known in the literature: indeed, they carry H/alkenyl and Me/Me substituents at a bridging ruthenium carbene site between the Cp* ligands.^4^, ^30^ Sterically demanding branched alkyl residues in lieu of the phenyl group are unlikely to fit into the groove formed by the Cp* rings and will hence prevent bridging carbene formation from occurring; the structure of the complex **20** discussed above bears witness of this notion.

The subtlety of the transformations described above is deemed remarkable: hydrogenation of enynes carrying a tethered carbonyl group with the help of [Cp*RuCl]_4_ as the catalyst invariably affords highly substituted furan products (Scheme 6). Depending on the nucleophilicity of the carbonyl substituent, however, the reaction takes place along two distinctly different pathways, each of which involves pianostool ruthenium carbenes as the key intermediates. These reactive species evolve, however, on the opposite ends of the central alkyne subunit of the substrate. In the first case, the π‐complex initially formed binds H_2_, which in turn triggers the unorthodox *gem*‐hydrogenation of the triple bond with concomitant formation of a ruthenium carbene;^1^, ^2^, ^3^, ^4^ hydrogenation and carbene formation are hence synchronized. Attack of the tethered ester carbonyl onto the electrophilic carbene center delivers the heterocyclic product and, at the same time, regenerates the catalyst.

**Scheme 6:**
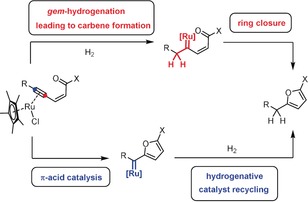
Conceptual framework featuring two unorthodox roles that hydrogenation can gain in metal carbene chemistry.

Alternatively, direct attack of a ketone onto the ligated alkyne outperforms *gem*‐hydrogenation.^31^ The ensuing five‐*exo‐dig* cyclization places the emerging ruthenium carbene away from the incoming nucleophile. In this π‐acid catalysis scenario, carbene formation precedes the actual hydrogenation reaction, which is necessary to regenerate the catalyst and ensure turn‐over. Insertions of either free carbenes or metal carbene complexes into H_2_ are known;^32^, ^33^ to the best of our knowledge, however, the furan synthesis described herein is the first example in which carbene hydrogenation is essential for the release of the desired product and catalyst recovery alike; it therefore keeps the actual catalytic carbene formation up and running.^34^ From the conceptual viewpoint, the chemistry described herein outlines new strategic roles for catalytic hydrogenation chemistry. Ongoing studies in our laboratory try to leverage some of the opportunities that this unconventional reactivity paradigm may provide.

## Conflict of interest

The authors declare no conflict of interest.

## Supporting information

As a service to our authors and readers, this journal provides supporting information supplied by the authors. Such materials are peer reviewed and may be re‐organized for online delivery, but are not copy‐edited or typeset. Technical support issues arising from supporting information (other than missing files) should be addressed to the authors.

Supplementary
