# Supplementary material for: Mechanistic Divergence in the Hydrogenative Synthesis of Furans and Butenolides: Ruthenium Carbenes Formed by gem‐Hydrogenation or through Carbophilic Activation of Alkynes
Source: Angew Chem Int Ed Engl. 2019 Nov 6;58(51):18476–81. doi: 10.1002/anie.201912161 (PMC6916381; doi:10.1002/anie.201912161)
Supplement: Supplementary file 1 — Supplementary [file ANIE-58-18476-s001.pdf]

Angewandte Chemie
Eine Zeitschrift der Gesellschaft Deutscher Chemiker

Supporting Information

Mechanistic Divergence in the Hydrogenative Synthesis of Furans and Butenolides: Ruthenium Carbenes Formed by *gem*-Hydrogenation or through Carbophilic Activation of Alkynes

*Sebastian Peil and Alois Fürstner**

anie_201912161_sm_miscellaneous_information.pdf

SUPPORTING INFORMATION

Contents

SUPPORTING CRYSTALLOGRAPHIC INFORMATION........ 2
GENERAL........ 8
PREPARATION OF CARBENES........ 8
H_2/D_2 AND PHIP EXPERIMENTS........ 16
SUBSTRATES........ 20
FURAN AND BUTENOLIDE SYNTHESES........ 28
REFERENCES........ 74

SUPPORTING CRYSTALLOGRAPHIC INFORMATION

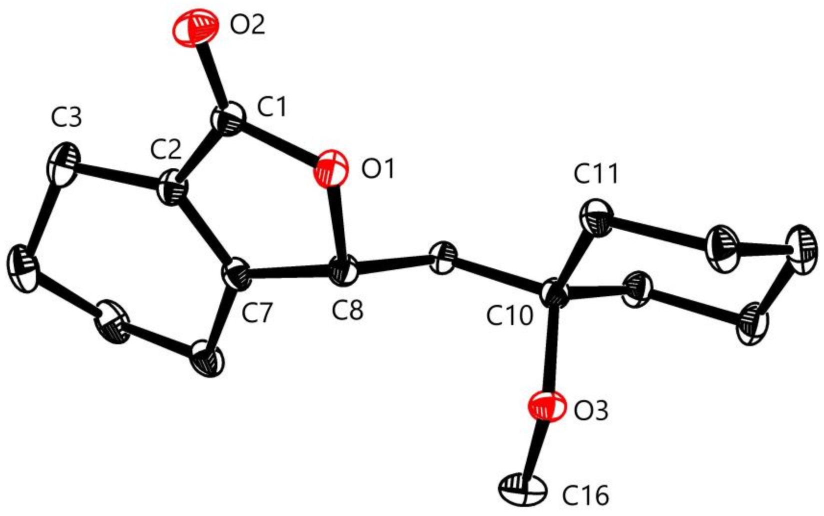

Figure S1. The structure of butenolide **9** in the solid state; H-atoms are omitted for clarity

X-ray Crystal Structure Analysis of Comopound 9: $C_{16}H_{24}O_3$, *Mr* = 264.35 g · mol^{-1}, colorless prism, crystal size 0.46 x 0.25 x 0.14 mm^3, triclinic, space group $P\bar{1}$, *a* = 7.1702(3) Å, *b* = 9.5363(10) Å, *c* = 12.0112(8) Å, α = 67.247(6)°, α = 87.597(7)°, γ = 73.737(7)°, *V* = 725.03(10) Å^3, *T* = 100(2) K, *Z* = 2, D_{calc} = 1.211 g · cm^3, λ= 0.71073 Å, μ(*Mo-K*α) = 0.082 mm^{-1}, Gaussian absorption correction (T_{min} = 0.97, T_{max} = 0.99), Bruker-AXS Kappa Mach3 APEX-II diffractometer, 3.359 < Θ < 39.039°, 40383 measured reflections, 8426 independent reflections, 6488 reflections with $I > 2\sigma(I)$, R_{int} = 0.0370.

The structure was solved by direct methods and refined by full-matrix least-squares against F^2 to R_1 = 0.041 [$I > 2\sigma(I)$], wR_2 = 0.120, 268 parameters. The H atoms were found and refined, *S* = 1.073, residual electron density 0.4 (0.64 Å from C1)/ -0.3 (1.13 Å from C7) e · Å^{-3}. **CCDC- 1954857**.

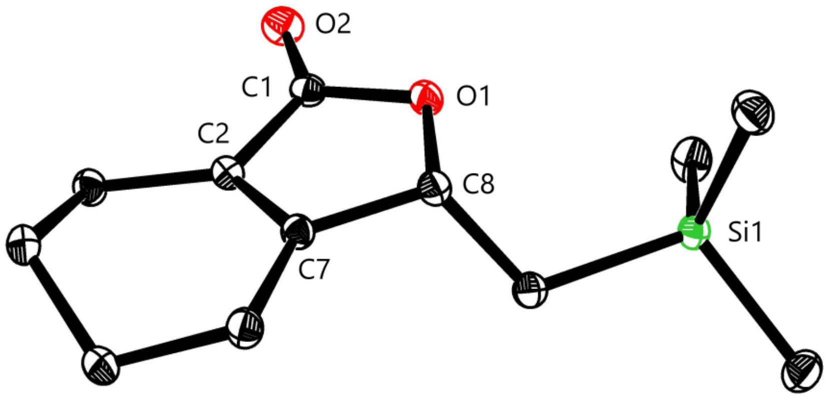

Figure S2. The structure of butenolide **11** in the solid state; H-atoms are omitted for clarity

X-ray Crystal Structure Analysis of Compound 11: $C_{12}H_{20}O_2Si$, *Mr* = 224.37 g · mol^{-1}, colourless plate, crystal size 0.312 x 0.142 x 0.045 mm^3, triclinic, space group $P\bar{1}$, *a* = 6.8743(4) Å, *b* = 9.8221(5) Å, *c* = 10.0180(5) Å, α = 107.556(2)°, β = 91.128(2)°, γ = 99.904(2)°, *V* = 633.45(6) Å^3, *T* = 100(2) K, *Z* = 2, D_{calc} = 1.176 g · cm^3, λ = 0.71073 Å, μ(*Mo-K*α) = 0.166 mm^{-1}, Gaussian absorption correction (T_{min} = 0.94, T_{max} = 1.00), Bruker-AXS Kappa Mach3 APEX-II diffractometer, 2.558 < Θ < 32.031°, 22101 measured reflections, 4400 independent reflections, 3963 reflections with $I > 2\sigma(I)$, R_{int} = 0.0208.

The structure was solved by direct methods and refined by full-matrix least-squares against F^2 to R_1 = 0.030 [$I > 2\sigma(I)$], wR_2 = 0.083, 139 parameters. The H atoms were refined using a riding model, *S* = 1.044, residual electron density 0.5 (0.70 Å from C2)/ -0.2 (0.79 Å from C1) e · Å^{-3}. **CCDC- 1954855**.

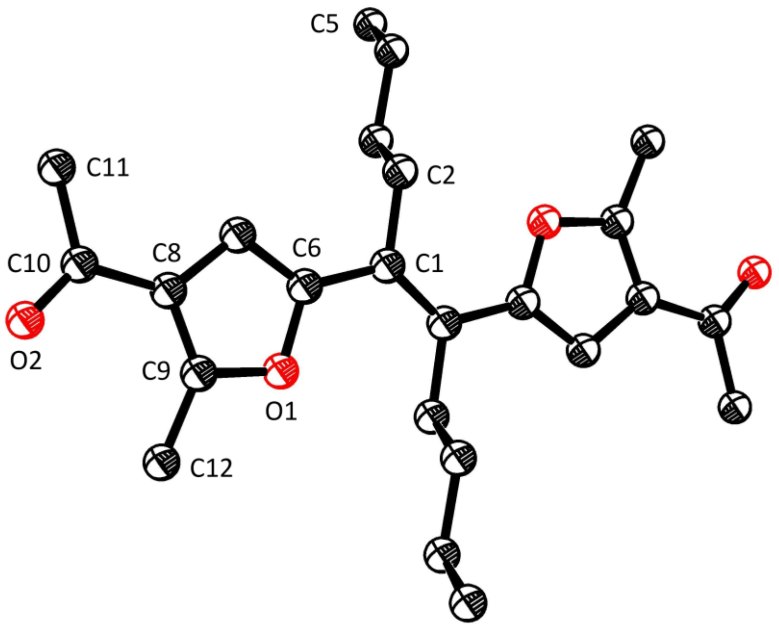

Figure S3. The structure of olefin **26 (R = *n*-Bu)** in the solid state; H-atoms are omitted for clarity

X-ray Crystal Structure Analysis of Compound 26 (R = *n*-Bu): $C_{24}H_{32}O_4$, *Mr* = 384.49 g · mol^{-1}, yellow prism, crystal size 0.141 x 0.097 x 0.090 mm^3, triclinic, space group $P\bar{1}$, *a* = 5.0966(3) Å, *b* = 10.6198(6) Å, *c* = 11.4706(6) Å, ⍰ = 62.787(2)°, β = 85.006(3)°, γ = 81.435(3)°, *V* = 545.84(5) Å^3, *T* = 150(2) K, *Z* = 1, D_{calc} = 1.170 g · cm^3, λ = 0.71073 Å, μ(*Mo-Kα*) = 0.078 mm^{-1}, Gaussian absorption correction (T_{min} = 0.99, T_{max} = 1.00), Bruker-AXS Kappa Mach3 APEX-II diffractometer, 3.554 < Θ < 33.949°, 19787 measured reflections, 4365 independent reflections, 3122 reflections with $I > 2\sigma(I)$, R_{int} = 0.0303.

The structure was solved by direct methods and refined by full-matrix least-squares against F^2 to R_1 = 0.048 [$I > 2\sigma(I)$], wR_2 = 0.136, 130 parameters. The H atoms were refined using a riding model, *S* = 1.034, residual electron density 0.3 (0.67 Å from C8)/ -0.2 (0.31 Å from H12A) e · Å^{-3}. **CCDC- 1954856**.

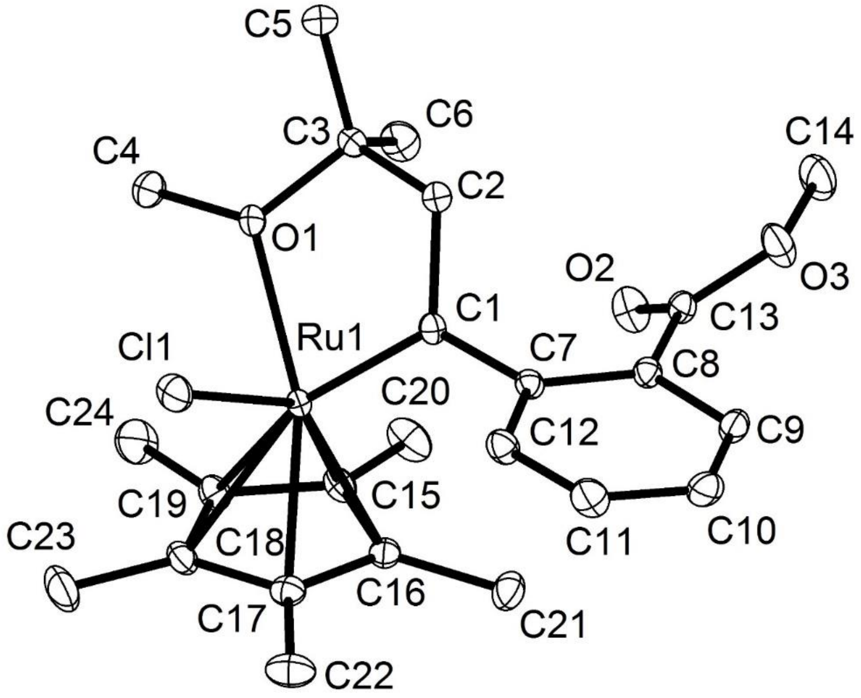

Figure S4. The structure of carbene complex **14** in the solid state; H-atoms are omitted for clarity

X-ray Crystal Structure Analysis of Complex 14: $C_{24}H_{33}ClO_3Ru$, *Mr* = 506.02 g · mol^{-1}, orange-red plate, crystal size 0.102 x 0.082 x 0.021 mm^3, monoclinic, space group *P*n, *a* = 8.2751(7) Å, *b* = 11.1713(10) Å, *c* = 12.4844(11) Å, β = 92.485(3)°, *V* = 1153.02(17) Å^3, *T* = 100(2) K, *Z* = 2, D_{calc} = 1.458 g · cm^3, λ = 0.71073 Å, μ(*Mo-K*α) = 0.817 mm^{-1}, Gaussian absorption correction (T_{min} = 0.94, T_{max} = 0.98), Bruker-AXS Kappa Mach3 APEX-II diffractometer, 3.015 < Θ < 35.129°, 82084 measured reflections, 10151 independent reflections, 9700 reflections with $I > 2\sigma(I)$, R_{int} = 0.0348.

The structure was solved by direct methods and refined by full-matrix least-squares against F^2 to R_1 = 0.019 [$I > 2\sigma(I)$], wR_2 = 0.044, 271 parameters, Absolute structure parameter = -0.022(6).

The H atoms were refined using a riding model, *S* = 1.031, residual electron density 0.5 (0.72 Å from Ru1)/ -0.4 (0.66 Å from Ru1) e · Å^{-3}. **CCDC- 1954859**.

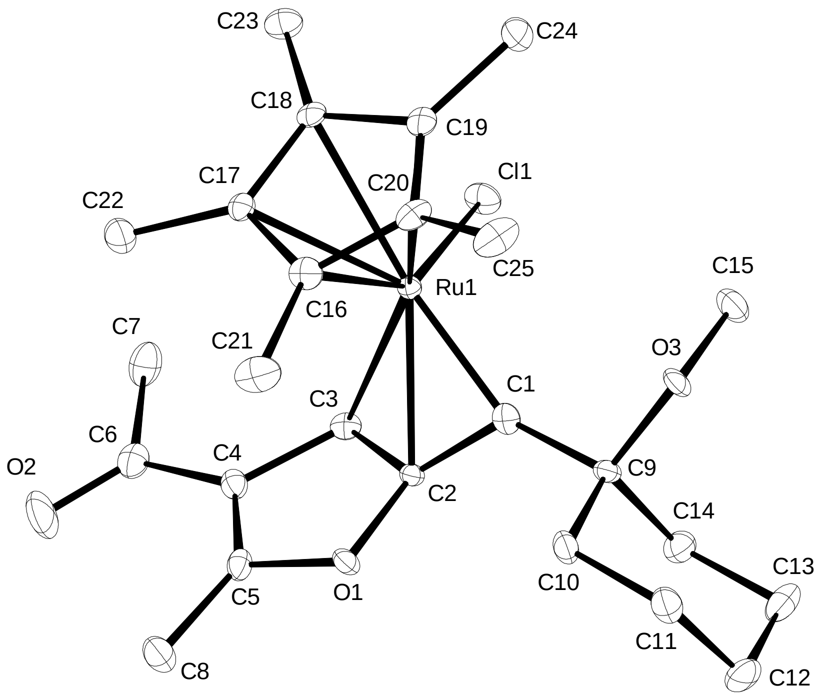

Figure S5. The structure of furyl carbene **20** in the solid state; H-atoms are omitted for clarity

X-ray Crystal Structure Analysis of Complex 20: $C_{25} H_{35} Cl O_3 Ru$, *Mr* = 520.05 g · mol^{-1}, red prism, crystal size 0.062 x 0.024 x 0.022 mm^3, monoclinic, space group $P2_1/c$, *a* = 19.8935(7) Å, *b* = 8.4325(3) Å, *c* = 14.2252(5) Å, β = 100.151(2)°, *V* = 2348.95(14) Å^3, *T* = 100(2) K, *Z* = 4, D_{calc} = 1.471 g · cm^3, λ = 0.71073 Å, μ(*Mo-K*α) = 0.805 mm^{-1}, Gaussian absorption correction (T_{min} = 0.97, T_{max} = 0.99), Bruker-AXS Kappa Mach3 APEX-II diffractometer, 1.040 < Θ < 27.500°, 46004 measured reflections, 5379 independent reflections, 4220 reflections with $I > 2\sigma(I)$, R_{int} = 0.0752.

The structure was solved by direct methods and refined by full-matrix least-squares against F^2 to R_1 = 0.034 [$I > 2\sigma(I)$], wR_2 = 0.067, 806 parameters. The H atoms were refined using a riding model, *S* = 1.024, residual electron density 0.5 (0.78 Å from C14)/ -0.7 (0.87 Å from Ru1) e · Å^{-3}. **CCDC- 1954858**.

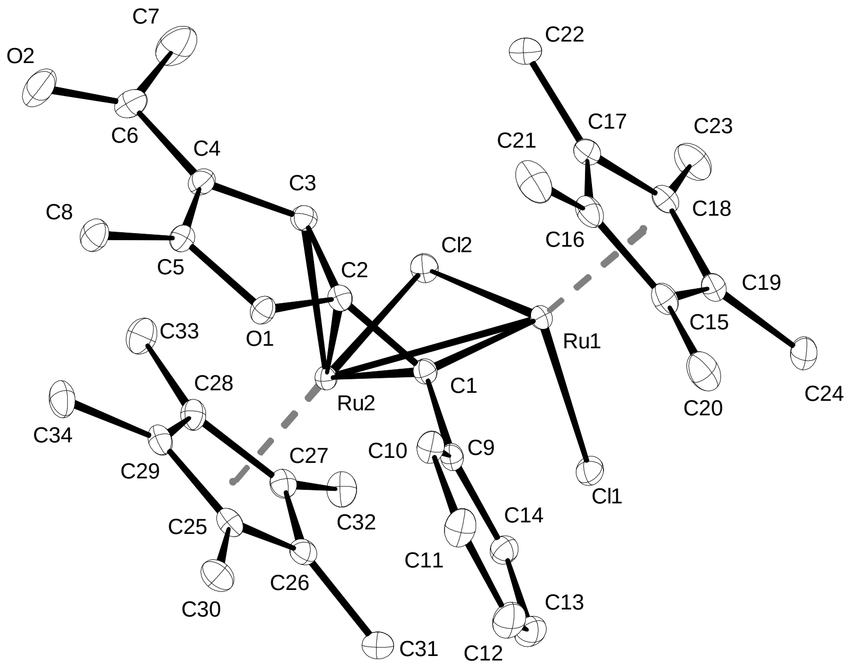

Figure S6. The structure of bridged carbene complex **27** in the solid state; H-atoms are omitted for clarity

X-ray Crystal Structure Analysis of Complex 27: $C_{34}H_{42}Cl_2O_2Ru_2$, Mr = 755.71 g · mol^{-1}, dark green block, crystal size 0.26 x 0.18 x 0.11 mm^3, monoclinic, space group $P2_1/n$, a = 10.0450(14) Å, b = 16.958(2) Å, c = 18.0338(4) Å, β = 93.416(3)°, V = 3066.4(6) Å^3, T = 100(2) K, Z = 4, D_{calc} = 1.637 g · cm^3, λ = 0.71073 Å, $\mu(Mo\text{-}K\alpha)$ = 1.189 mm^{-1}, Gaussian absorption correction (T_{min} = 0.74, T_{max} = 0.88), Bruker-AXS Kappa Mach3 APEX-II diffractometer, 3.146 < Θ < 37.057°, 159402 measured reflections, 15613 independent reflections, 13022 reflections with $I > 2\sigma(I)$, R_{int} = 0.0388.

The structure was solved by direct methods and refined by full-matrix least-squares against F^2 to R_1 = 0.023 [$I > 2\sigma(I)$], wR_2 = 0.055, 377 parameters. The H atoms were refined using a riding model, S = 1.045, residual electron density 1.2 (0.98 Å from Ru2)/ -1.3 (0.66 Å from Ru2) e · Å^{-3}. **CCDC- 1954860**.

GENERAL

Unless stated otherwise, all reactions were carried out under argon atmosphere in flame dried Schlenk glassware. The solvents were purified by distillation over the indicated drying agents under argon: THF, Et_2O (Mg/anthracene), hexanes (Na/K), EtOH, MeOH (Mg), 1,2-dichloroethane, CD_2Cl_2, CH_2Cl_2 (CaH_2). DMF, MeCN and Et_3N were dried by an absorbtion solvent purification system based on molecular sieves. 1,2-Dichloroethane, CD_2Cl_2 and CH_2Cl_2 were degassed via freeze-pump-thaw procedure (3 x) and stored over molecular sieves. Column chromatography: Merck Geduran silica gel 60 (40 – 63 µm). NMR spectra were recorded on Bruker DPX 300, AMX 300, AV 400, AV III 500 or AV III 600 spectrometers in the solvents indicated; chemical shifts are given in ppm relative to TMS, coupling constants (*J*) in Hz. The solvent signals were used as references and the chemical shifts converted to the TMS scale ($CDCl_3$: δ_C = 77.16 ppm; residual $CHCl_3$: δ_H = 7.26 ppm; CD_2Cl_2: δ_C = 54.00 ppm; residual $CHDCl_2$: δ_H = 5.32 ppm). Proton and carbon assignments were established using HSQC, HMBC and NOESY experiments. PHIP NMR experiments were acquired on a Bruker AVIII 500 MHz (11.7 T) NMR Magnet equipped with a BBFO probe with z-gradient. OPSY spectra were recorded using the opsy-d pulse sequence.[1] *para*-Hydrogen enriched to 92% was freshly generated using a commercially available *p*-H_2 Generator from Bruker BioSpin GmbH with an F–DGSi electrolytic hydrogen generator (WM.H2.500.V3) as the hydrogen source. IR: Alpha Platinum ATR (Bruker), wavenumbers ($\tilde{\nu}$) in cm^{-1}. MS (EI): Finnigan MAT 8200 (70 eV), ESI-MS: ESQ 3000 (Bruker), Thermo Scientific LTQ-FT or Thermo Scientific Exactive. HRMS: Bruker APEX III FT-MS (7 T magnet), MAT 95 (Finnigan), Thermo Scientific LTQ-FT or Thermo Scientific Exactive. GC-MS: Shimadzu GCMS-QP2010 Ultra instrument.

Unless stated otherwise, all commercially available compounds (abcr, Acros, TCI, Aldrich, Alfa Aesar) were used as received. The ruthenium complex $[Cp^*RuCl]_4$ [2] was prepared according to the literature procedure. Intermediates for the synthesis of substrates were prepared according to the cited literature (see below).

PREPARATION OF CARBENES

***gem*-Hydrogenation: Carbene Complex 14.** $[Cp^*RuCl]_4$ (91.0 mg, 0.08 mmol) was added to a stirred solution of enyne **13** (77.8 mg, 0.33 mmol) in CH_2Cl_2 (7.9 mL, 0.04 M) in a flame dried Schlenk tube at 0 °C under argon. H_2 was bubbled through the mixture for 2 min before the mixture was stirred for 1 h at 0 °C under am hydrogen atmosphere (balloon). The solvent was removed by purging the mixture with argon and subsequent evacuation of the tube. The brown solidified foam was dissolved in CD_2Cl_2 and immediately subjected to NMR analysis. Crystals suitable for X-ray analysis were obtained by extracting the residue with pentane (3 mL) at 0 °C followed by slow cooling the resulting solution to −55 °C over the course of 36 h (fine brown needles).

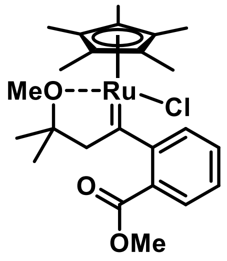

^1H NMR (500 MHz, −50 °C, CD_2Cl_2) δ 8.00 (dd, *J* = 7.6, 1.4 Hz, 1H), 7.64 (dd, *J* = 7.6, 1.4 Hz, 1H), 7.43 (td, *J* = 7.6, 1.4 Hz, 1H), 7.36 (td, *J* = 7.6, 1.4 Hz, 1H), 3.77 (s, 3H), 3.31 (s, 3H), 2.19 (d, *J* = 18.0 Hz, 1H), 1.51 (d, *J* = 18.0 Hz, 1H), 1.27 (s, 15H), 1.21 (s, 3H), 1.18 (s, 3H). ^{13}C NMR (101 MHz, −50 °C, CD_2Cl_2) δ 302.2, 168.8, 161.0, 131.2, 129.0,

126.4, 119.9, 93.0, 85.1, 70.8, 55.3, 52.6, 22.8, 22.6, 9.9. IR (solid) ṽ 2904, 1764, 1720, 1436, 1275, 1252, 1073 cm^{-1}. HRMS (ESI^{+}) for $C_{24}H_{33}ClO_3Ru$ $[M]^{+}$: calcd 506.1162, found 506.1156.

Table S1: NMR signal assignments of complex **14** (500 MHz, −50 °C, CD_2Cl_2)

Atom [#] C	H	δ [ppm]	J [Hz]	HMBC	NOESY
**1**		52,64		1	
	**1**	3,77		1, 2	4, 10a, 12, 16
**2**		168,76		1, 4	
**3**		119,93		5, 7	
**4**		129,00		6	
	**4**	7,64	d 1.4, d 7.6	2, 6, 8	1, 16
**5**		126,42		7	
	**5**	7,36	d 1.4, d 7.4, d 7.6	3, 7	16
**6**		131,22		4	
	**6**	7,43	d 7.6, d 7.4, d 1.4	4, 8	16
**7**		129,00		5, 7	
	**7**	8,01	d 7.6, d 1.4	3, 5, 7, 9	10b, 13, 16
**8**		161,02		4, 6, 10a, 10b	
**9**		302,16		7, 10a, 10b	
**10**		70,82		10b, 12, 13	
	**10a**	2,19	d 18.0	8, 9, 11, 12, 13	1, 12, 16
	**10b**	1,51	d 18.0	8, 9, 10, 11, 12, 13	7, 13
**11**		85,05		10a, 10b, 12, 13, 14	
**12**		22,55		10a, 10b, 13	
	**12**	1,18		10, 11, 13	1, 10a, 14, 16
**13**		22,77		10a, 10b, 12	
	**13**	1,21		10, 11, 12	7, 10b, 14
**14**		55,28			
	**14**	3,31		11	12, 13, 16
**15**		93,04		16	
**16**		9,85		16	
	**16**	1,27		15, 16	1, 4, 5, 6, 7, 10a, 12, 14

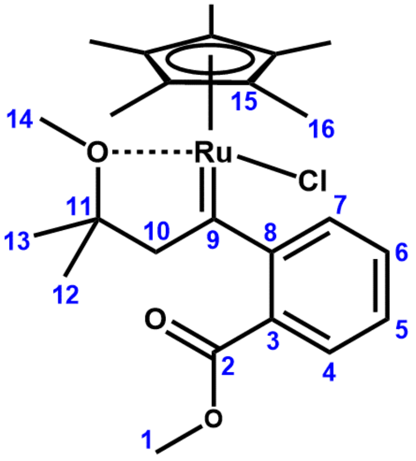

Furyl Carbene Complex 20. $[Cp^*RuCl]_4$ (109 mg, 0.1 mmol) was added to a stirred solution of enyne **15** (100 mg, 0.4 mmol) in CH_2Cl_2 (9 mL) in a flame dried Schlenk tube at room temperature under argon. The mixture was stirred for 30 min before the solvent was removed in vacuo. The crude material was triturated with pentane (3 mL) and subsequently extracted with Et_2O (3 mL). The extract was cooled from 25 °C to −50 °C over the course of 48 h to obtain deep burgundy colored prisms. ^{1}H NMR (400 MHz, CD_2Cl_2) δ 6.79 (s, 1H), 3.50 (s, 3H), 2.45 (s, 3H), 2.44 (s, 3H), 2.26 – 2.13 (m, 2H), 2.01 (m, 1H), 1.86 – 1.70 (m, 2H), 1.64 – 1.53 (m, 4H), 1.52 (s, 15H), 1.42 – 1.29 (m, 1H). ^{13}C NMR (101 MHz, $CDCl_3$) δ 266.7, 194.4, 165.7, 132.3, 124.4, 100.1, 96.6, 89.7, 53.1, 32.7, 29.7, 27.4, 25.8, 21.6, 15.7, 10.8. IR (solid) ṽ 2927, 1672, 1566, 1378, 1196, 1068 cm^{-1}. HRMS (ESI$^+$) for $C_{25}H_{35}O_3RuCl$ $[M]^+$: calcd 520.1313, found 520.1321.

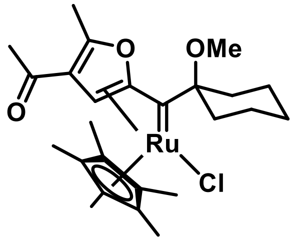

Table S2: NMR signal assignments of complex **20** (400 MHz, 25 °C, CD_2Cl_2)

Atom [#] C	H	δ [ppm]	J [Hz]	HMBC	NOESY
1		32.08			
	1	2,03; 2,22			
2		20,99			
	2	1,81; 1,58			
3		25,15			
	3	1,78; 1,37			
4		20,99			
	4	1,81; 1,58			
5		26.80			
	5	2,19; 1,58			
6		99,53		15	
7		266,08			
8		131,76		9	
9		89,08		12, 14	
	9	6,82		8, 13, 11, 10	14
10		123,86		9, 12, 14	
11		165,11		9, 12	
12		15,13			
	12	2,47		13, 11, 10, 9	
13		193,83		9, 12, 14	
14		29,17			
	14	2,48		13, 10, 9	9
15		52,52			
	15	3,53		6	1, 5
16		96,05		17	
17		10,21			
	17	1,55		16	1, 9, 12, 14, 15

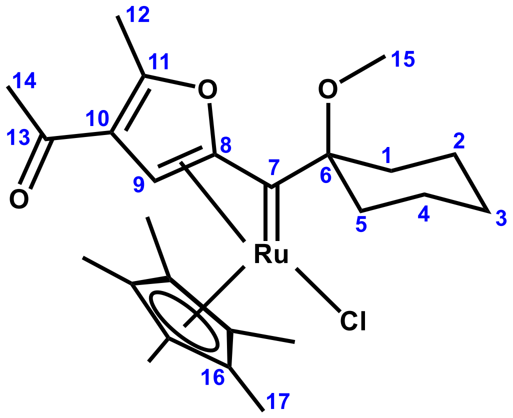

Furyl Carbene Complex 22 (R = *t*Bu). [Cp*RuCl]$_4$ (12.2 mg, 0.01 mmol) was added to a stirred solution of enyne **21** (R = *t*Bu) (8.6 mg, 0.044 mmol) in CH_2Cl_2 (0.8 mL, 0.06 M) in a flame dried Schlenk tube at room temperature under argon. The mixture was stirred for 30 min before the solvent was removed by purging of the mixture with argon and subsequent evacuation of the tube to give the complex as a brown solid (quant.). ^1H NMR (400 MHz, CD_2Cl_2) δ 6.26 (d, *J* = 0.7 Hz, 1H), 2.48 (d, *J* = 0.6 Hz, 3H), 2.45 (s, 3H), 1.53 (s, 15H), 1.45 (s, 9H). ^{13}C NMR (101 MHz, CD_2Cl_2) δ 285.3, 194.5, 168.6, 123.0, 118.2, 98.1, 80.3, 52.2, 29.8, 27.1, 15.7, 11.0. IR (solid) ṽ 2965, 2908, 1671, 1578, 1260, 1216, 1018 cm^{-1}. HRMS (ESI$^+$) for $C_{22}H_{31}ClO_2Ru$ [M]$^+$: calcd 464.1051, found 464.1047.

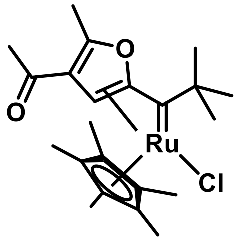

Table S3: NMR signal assignments of complex **22 (R = *t*Bu)** (400 MHz, 25 °C, CD_2Cl_2)

Atom [#] C	H	δ [ppm]	J [Hz]	HMBC	NOESY
1		27,05		1	
	1	1,45		1, 2, 3	
2		52,22		1	
3		285,25		1	
4		118,17		5	
5		80,29		10	
	5	6,26		4, 6, 7	10, 12
6		122,98		5, 8, 10	
7		168,62		5, 8	
8		15,67			
	8	2,48		6, 7	12
9		194,52		10	
10		29,82			
	10	2,45		5, 6, 9	5, 12
11		98,08		12	
12		10,95			
	12	1,53		11	5, 8, 10

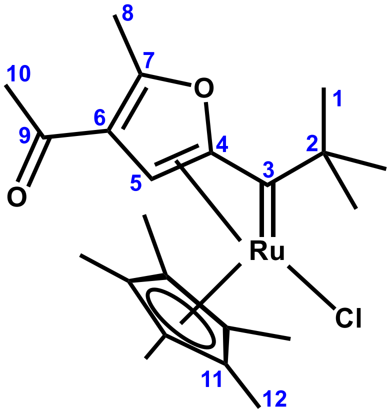

Bridged Carbene Complex 27. To a stirred solution of [Cp*RuCl]$_4$ (128 mg, 0.12 mmol) in CD_2Cl_2 (6 mL) was added a solution of enyne **21** (R = Ph) (50 mg, 0.24 mmol) in CD_2Cl_2 (6 mL) over 1 h via syring pump under argon at room temperature. The solvent was evaporated in vacuo and the residue triturated with pentane (3 mL) and subsequently extracted with Et_2O (3 mL). The extract was cooled from 25 °C to −50 °C over the course of 48 h to obtain dark green prisms. ^{1}H NMR (400 MHz, −50 °C, CD_2Cl_2) δ 8.89 (dt, *J* = 7.9, 1.5 Hz, 1H), 8.32 (dt, *J* = 7.5, 1.7 Hz, 1H), 7.36 (td, *J* = 8.1, 7.5, 2.2 Hz, 1H), 7.33 – 7.21 (m, 2H), 3.63 (s, 1H), 2.52 (s, 3H), 2.28 (s, 3H), 1.13 (s, 15H), 1.12 (s, 15H). ^{13}C NMR (101 MHz, −50 °C, CD_2Cl_2) δ 194.6, 189.9, 162.2, 150.7, 138.9, 133.7, 128.4, 126.5, 126.1, 124.5, 122.2, 76.2, 29.8, 15.5. IR (solid) ṽ 3051, 2966, 2904, 1669, 1579, 1373, 1021 cm^{-1}. HRMS (ESI$^+$) for $C_{34}H_{42}Cl_2O_2Ru_2$ [M]$^+$: calcd 756.0649, found 756.0633.

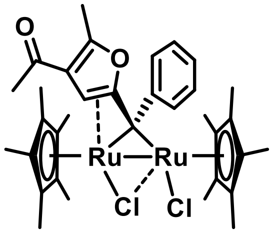

Table S4: NMR signal assignments of complex **27** (400 MHz, −50 °C, CD_2Cl_2)

Atom [#] C	H	δ [ppm]	J [Hz]	HMBC	NOESY
1		29,76			
	1	2,28		2, 3	4, 16
2		194,63		1	
3		122,2		1, 4, 14	
4		76,24			
	4	3,63		3, 5, 13	1, 16
5		133,75		4	
6		189,93		8, 12	
7		150,69		9, 11	
8		124,55		10, 12	
	8	8,32	7,7	6, 10, 12	9, 16
9		126,12		11	
	9	7,28	7,7	7, 11	8, 16
10		126,55		8, 12	
	10	7,28		8, 12	
11		128,41		9	
	11	7,36	8,1	7, 9	12, 16
12		138,92		8, 10	
	12	8,89	8,1	6, 8, 10	11, 16
13		162,23		4, 14	
14		15,49			
	14	2,52		3, 13	
15a		87,53		16a	
15b		94,13		16b	
16a		9,63			
	16a	1,12		15a	1, 4, 8, 9, 10, 11, 12, 14
16b		9,04			
	16b	1,13		15b	1, 4, 8, 9, 10, 11, 12, 14

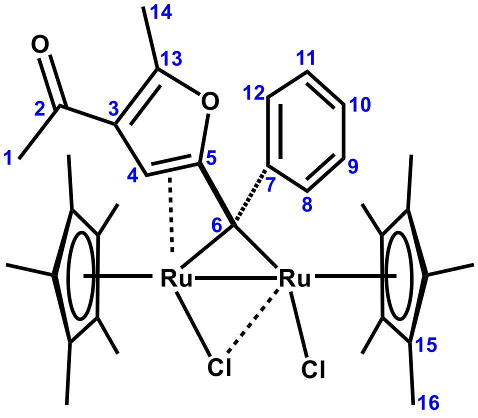

Dynamic Behavior of Complex 27. Complex **27** exhibits a marked dynamic behavior at room temperature (compared to −50 °C) as evident by NMR. At room temperature rotation around the carbene-phenyl bond (Fig. S7a) is slow enough to yield two well resolved ^{1}H resonances for H8 and H12 (arbitrary assignment). Nevertheless, exchange between these protons is evident by EXSY correlation (Fig. S8). At −50 °C this rotation is frozen out as indicated by the missing exchange correlation.

A second (potentially coupled) phenomenon concerns the ligand exchange dynamic about the homobimetallic core (Fig. S7b). At room temperature fast interconversion of **27** and **27'** (inversion) lead to a single ^{1}H Cp* resonance (Fig. S9), a broad ^{13}C Cp*(*Me*) resonance and no detectable ^{13}C Cp*(C_q) resonance (Fig. S10), likely due to extreme signal broadening. At −50 °C this interconversion is slow enough, such that two sets of Cp* signals corresponding to the two inequivalent Cp* fragments in **27** are detected.

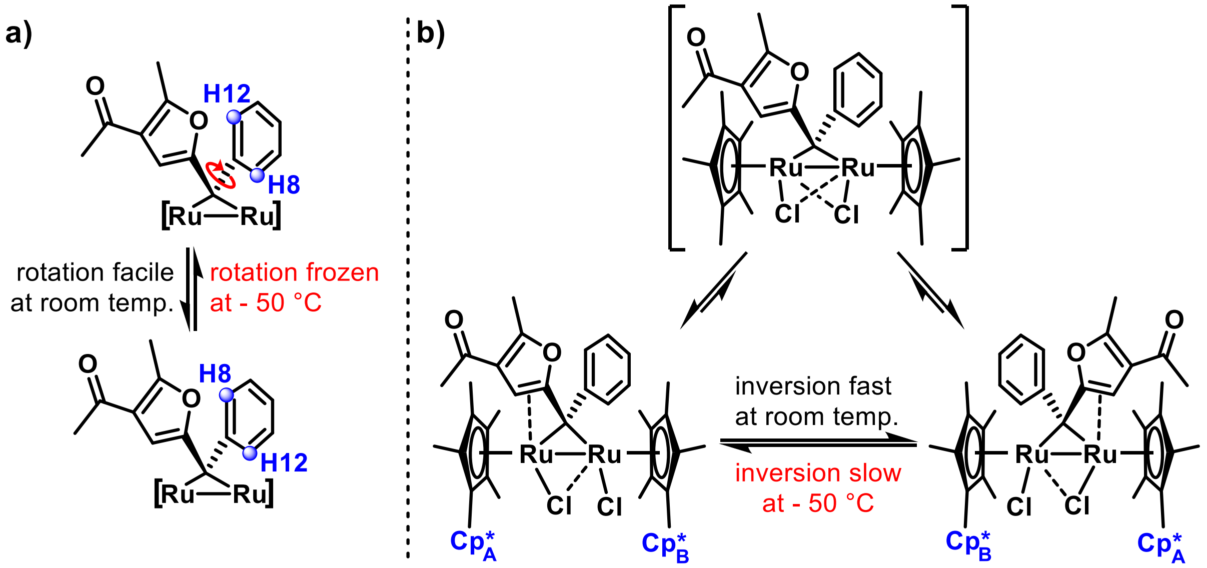

Figure S7. Dynamic behavior of bridging carbene complex **27**.

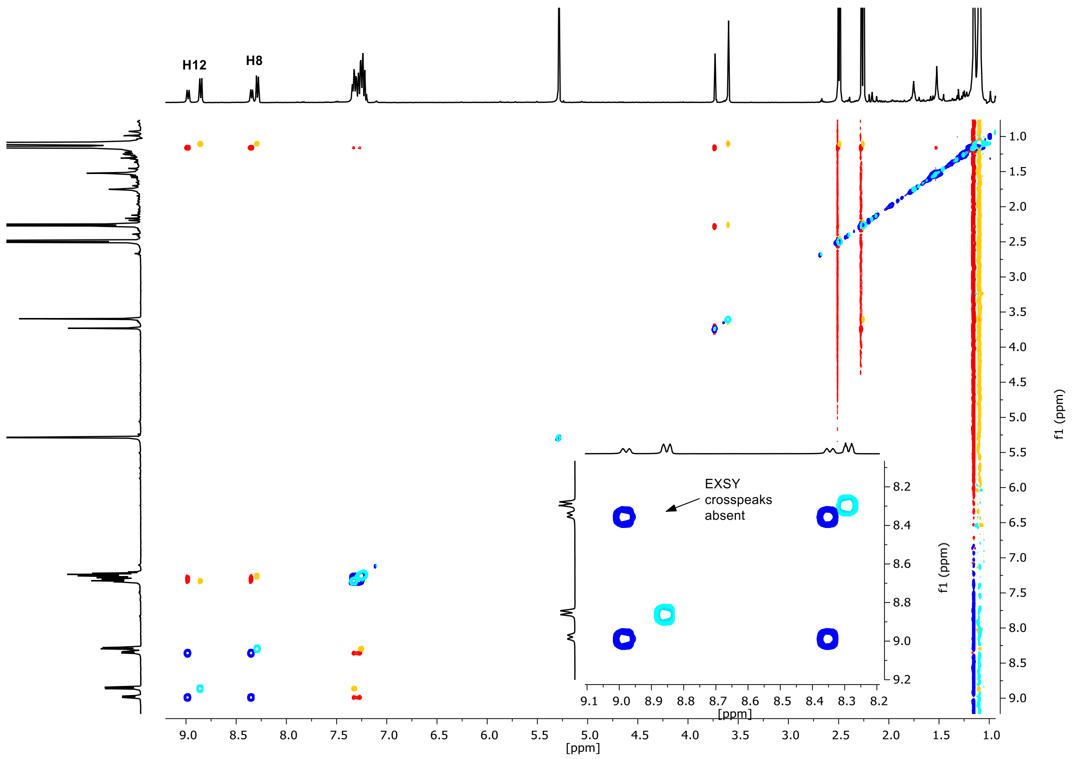

Figure S8. Overlay of EXSY/NOESY spectra of carbene **27** at 25 °C (red/blue) and −50 °C (orange/cyan). The insert reveals the missing exchange correlation between H8/H12 at low temperature. Horizontal and vertical trace spectra were generated by arithmetic addition of the separate 25 °C and −50 °C spectra.

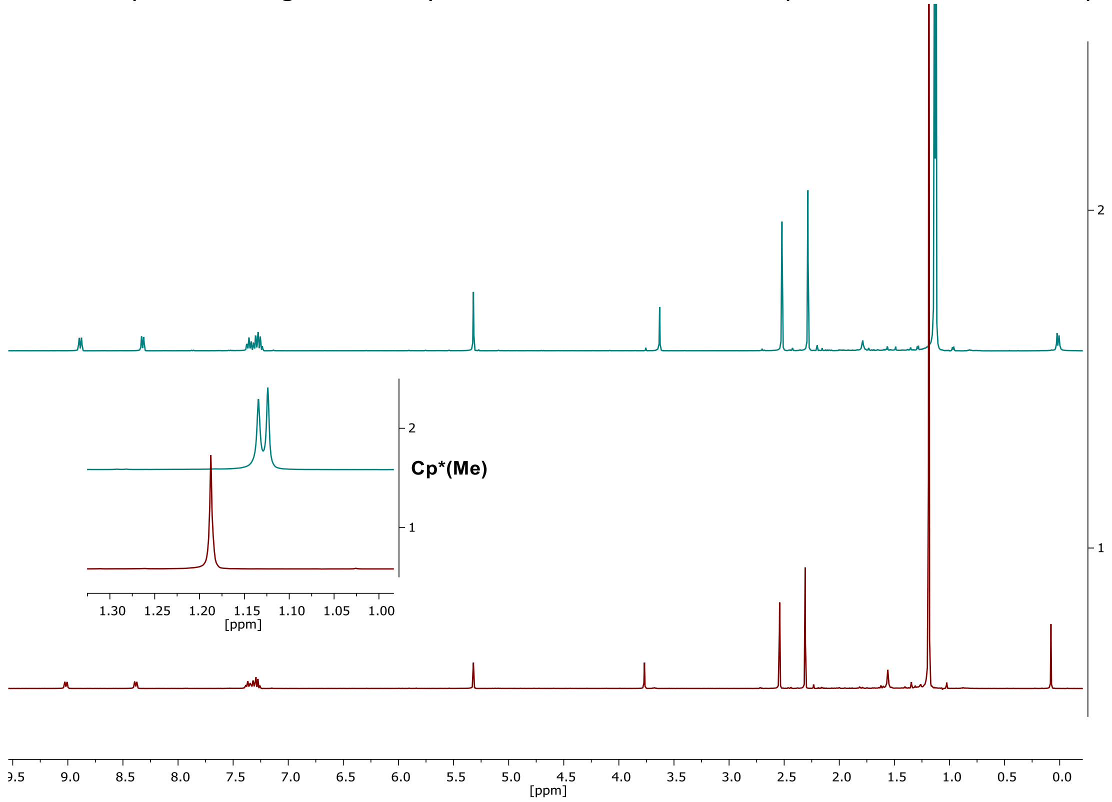

Figure S9. ^{1}H NMR spectra of complex **27** at room temperature (maroon) and −50 °C (turquoise); the insert shows the coalescence of the non-equivalent Cp* fragments at higher temperature.

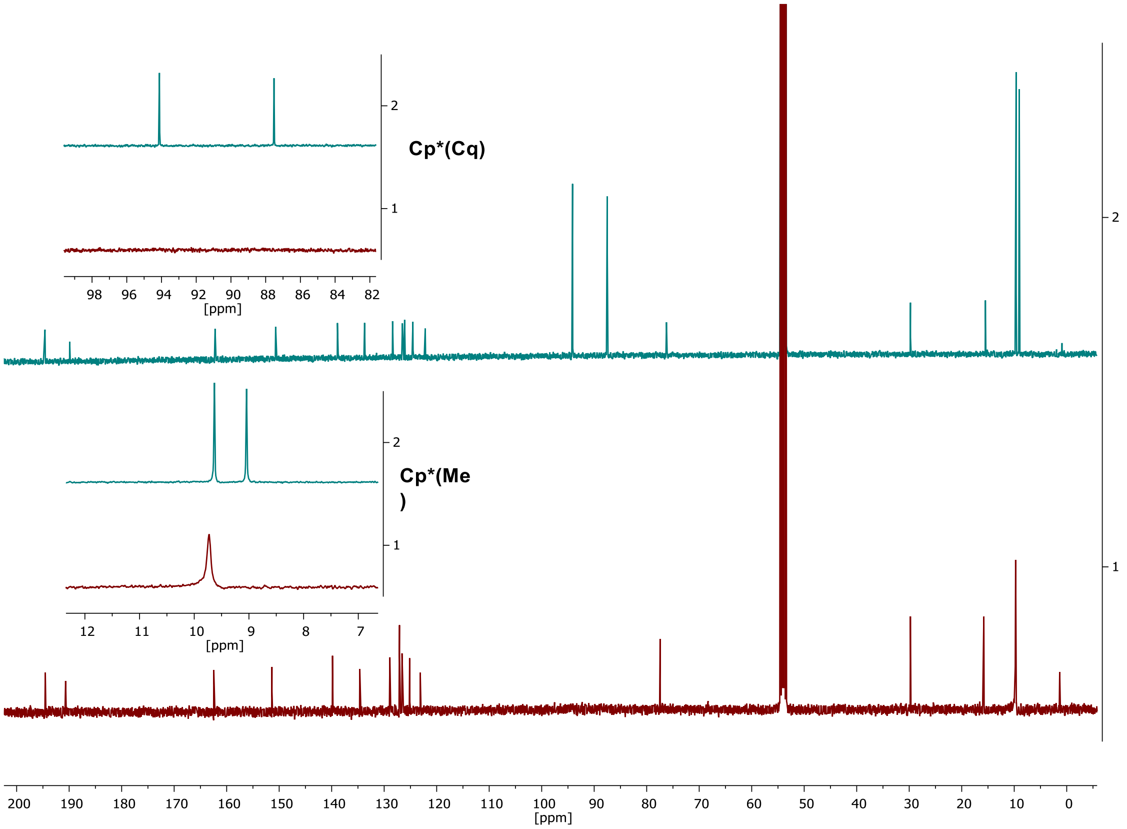

Figure S10. ^{13}C NMR spectra of complex **27** at room temperature (maroon) and −50 °C (turquoise); the inserts show the extreme line broadening of the signals of the quarternary C-atoms (C_q) of the Cp* ring (top) at room temperature and the coalescence of the Me-signals of the Cp* ring (bottom).

H_2/D_2 AND PHIP EXPERIMENTS

Competitive H_2/D_2 Carbene Insertion Experiments. The insertion of ruthenium carbene **22** (R = *t*Bu) was investigated by its reaction with H_2/D_2 (1:1 *v/v*) mixtures. Catalytic and stoichiometric experiments were performed as shown in Fig. S11.

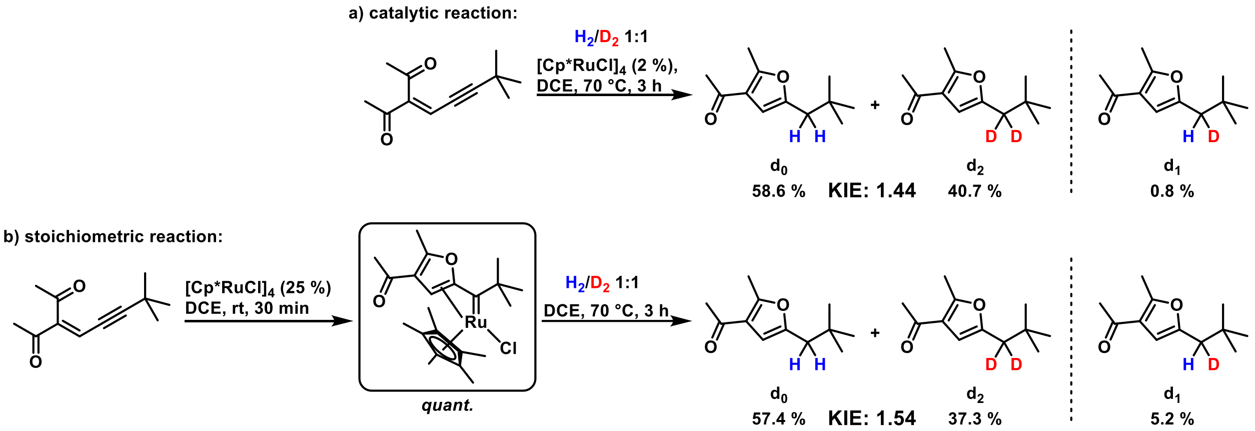

Figure S11. Catalytic and stoichiometric crossover and competition KIE experiments under H_2/D_2

Catalytic Reaction. The experiment was performed according to the Representative Procedure B (with enyne **21** (R = *t*Bu) (32.7 mg, 0.17 mmol) under an H_2/D_2 atmosphere (prepared by manually mixing H_2 and D_2 in a 10-mL-Hamilton syringe and injecting the mixture through a rubber septum). The product (colorless oil, 19.8 mg, 59 %) contains **[D$_0$]-23a** (58.6%), **[D$_2$]-23a** (40.7%) and traces of **[D$_1$]-23a** (determined by MS, see below). The analytical data of these compounds are contained in the product characterization section.

Stoichiometric Reaction. [Cp*RuCl]$_4$ (16.7 mg, 0.015 mmol) was added to a stirred solution of enyne **21** (R = *t*Bu) (11.8 mg, 0.061 mmol) in 1,2-dichloroethane (0.8 mL) in a flame dried Schlenk tube at room temperature under argon. The mixture was stirred for 30 min to complete carbene formation. H_2/D_2 (1:1) was then bubbled through the mixture before the mixture was stirred for 3 h at 70 °C under ambient pressure of H_2/D_2 (balloon). The solvent was removed under reduced pressure and the residue was purified by flash chromatography. The product (colorless oil, 86 %) contains **[D$_0$]-23a** (57.4%), **[D$_2$]-23a** (37.3%), and **[D$_1$]-23a** (5.2%).

```
No.  MW.  Comment

 1   194  D0 = 58.6% +/- 0.3%
          Compare E67500:
          overlapping with
 1   196  D2 = 40.7% +/- 0.3%
          Compare U20701:143262a-00 PEV-PA-531-01

Contains 0.8% +/- 0.3% D1

Other measurements will follow (HRMS)
```

```
31.07.2019
File: 143262a-00.raw

Analyse: PEV-PA-531-01
FUE: Peil, Sebastian
Messung:        GC-MS
Ionisierung:    GC-EI
Spektrometer:   ISQ
Säule:          MS 84 TG-5 SILMS
Länge:          28
Temp.:          35-10-285-5
GC-Nr.:         -
MS-Nr.:         21484
Auswerter:      Margold (2242)
```

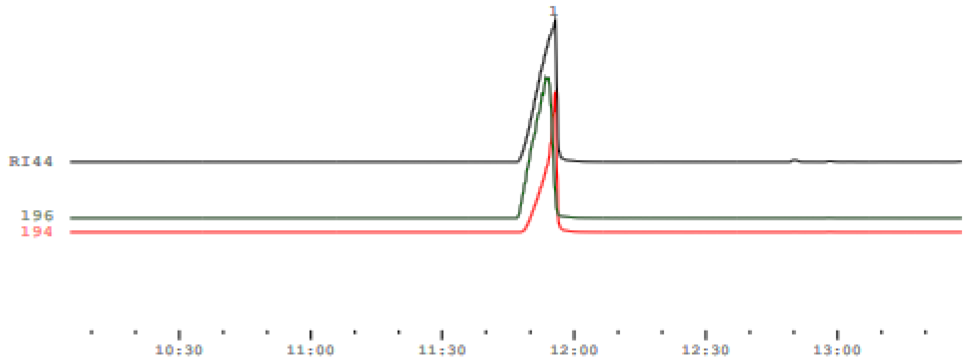

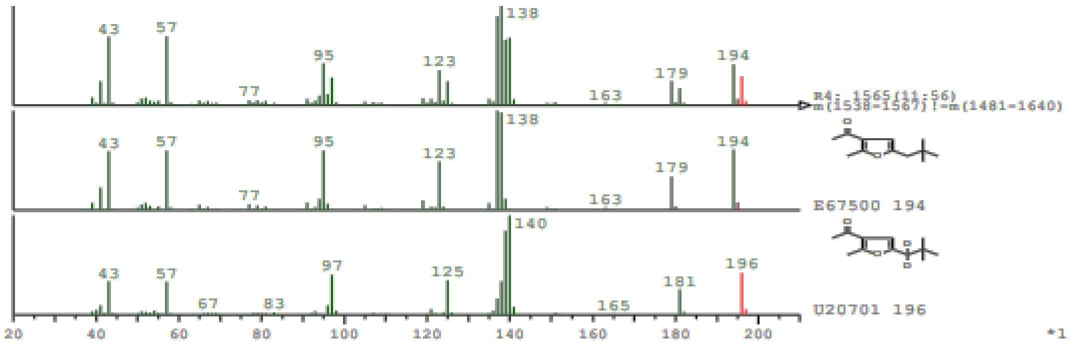

36	0.01	57	69.30	78	2.70	99	0.22	120	2.23	141	5.69	166	0.39
37	0.12	58	2.94	79	5.04	100	0.02	121	5.93	142	0.47	167	0.18
38	0.38	59	0.14	80	2.55	101	0.04	122	3.19	143	0.07	168	0.02
39	7.62	60	0.05	81	4.06	102	0.08	123	35.32	144	0.03	175	0.01
40	2.10	61	0.34	82	0.80	103	0.43	124	4.49	145	0.11	176	0.01
41	23.97	62	0.49	83	1.98	104	0.22	125	23.84	146	0.12	177	0.15
42	1.68	63	1.24	84	0.33	105	3.33	126	1.73	147	0.14	178	0.76
43	68.96	64	0.65	85	0.10	106	0.85	127	0.18	148	0.08	179	24.04
44	2.30	65	4.53	86	0.05	107	3.13	128	0.06	149	1.87	180	3.20
45	0.73	66	2.25	87	0.07	108	1.54	129	0.05	150	1.26	181	17.23
46	0.06	67	3.91	88	0.03	109	2.21	130	0.03	151	2.99	182	1.98
47	0.01	68	1.71	89	0.37	110	1.07	131	0.13	152	0.80	183	0.17
48	0.01	69	2.15	90	0.28	111	0.69	132	0.08	153	0.21	184	0.01
49	0.09	70	0.35	91	5.91	112	0.14	133	0.46	154	0.02	192	0.05
50	2.43	71	0.34	92	2.13	113	0.03	134	0.19	159	0.05	193	0.13
51	6.44	72	0.03	93	4.36	114	0.02	135	6.10	160	0.03	194	41.13
52	7.44	73	0.07	94	9.38	115	0.42	136	3.60	161	0.38	195	6.01
53	4.72	74	0.32	95	41.97	116	0.23	137	89.69	162	0.13	196	29.12
54	3.33	75	0.72	96	11.26	117	0.49	138	100.00	163	1.78	197	3.65
55	4.29	76	0.52	97	27.64	118	0.28	139	65.83	164	1.02	198	0.33
56	0.75	77	5.20	98	2.87	119	7.05	140	67.94	165	0.84	199	0.02

R4:1565(11:56) m(1538-1567) 143262a-00! -m(1481-1640) 17371 11% 143262a-00 * P lim: 0.01%

```
No.  MW.  Comment

 1   194  D0 = 57.4% +/- 0.1%
          Compare E67500:
          overlapping with
 1   196  D2 = 37.3% +/- 0.1%
          Compare U20701:143262a-00 PEV-PA-531-01

Contains 5.2% +/- 0.1% D1

Other measurements will follow (HRMS)
```

```
 9.08.2019
File: 143384a-00.raw
Analyse: PEV-PA-548-01
FUE: Peil, Sebastian
-----------------------------------
Messung:        GC-MS
Ionisierung:    GC-EI
Spektrometer:   ISQ Series
Säule:          MS 84 TG-5 SILMS
Länge:          28
Temp.:          35-10-285-5
GC-Nr.:         -
ELNA-Nr.:       21599
-----------------------------------
Auswerter:      Haupt (2243)
```

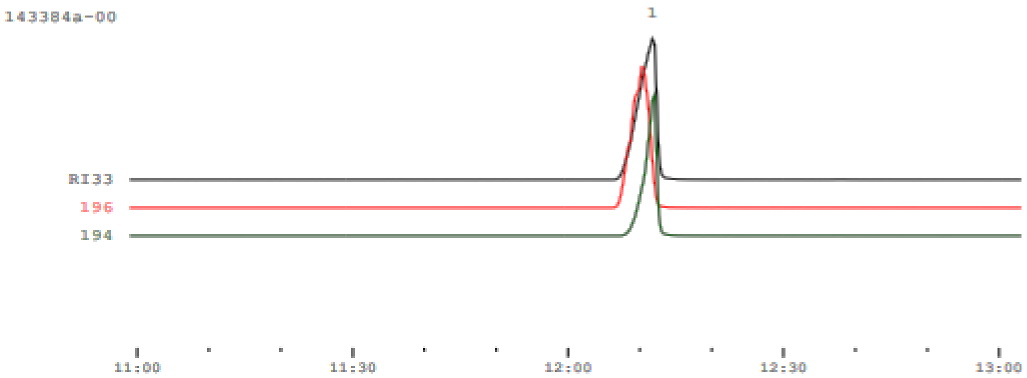

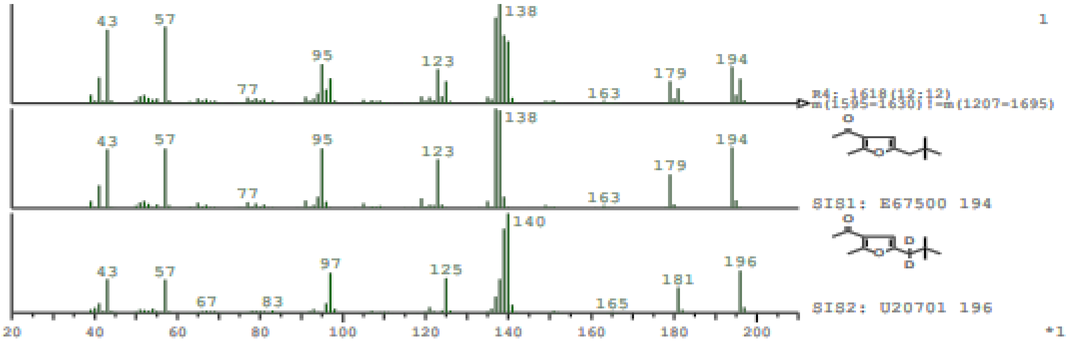

36	0.02	57	76.86	78	2.99	99	0.22	120	2.53	141	5.25	166	0.36
37	0.12	58	3.18	79	5.09	100	0.02	121	5.63	142	0.45	167	0.16
38	0.44	59	0.15	80	2.79	101	0.05	122	3.20	143	0.06	168	0.02
39	8.42	60	0.05	81	3.94	102	0.08	123	33.98	144	0.03	175	0.01
40	2.30	61	0.37	82	0.97	103	0.45	124	6.90	145	0.10	176	0.02
41	25.60	62	0.52	83	1.93	104	0.20	125	21.81	146	0.12	177	0.15
42	2.12	63	1.35	84	0.35	105	3.28	126	1.62	147	0.13	178	0.65
43	73.87	64	0.69	85	0.10	106	1.05	127	0.18	148	0.07	179	21.81
44	2.58	65	4.78	86	0.05	107	2.97	128	0.06	149	1.78	180	4.67
45	0.75	66	2.47	87	0.07	108	1.55	129	0.05	150	1.34	181	14.92
46	0.06	67	3.91	88	0.03	109	2.20	130	0.03	151	2.82	182	1.77
47	0.02	68	1.87	89	0.41	110	1.09	131	0.12	152	0.74	183	0.16
48	0.01	69	2.11	90	0.24	111	0.63	132	0.08	153	0.20	184	0.01
49	0.13	70	0.38	91	5.96	112	0.12	133	0.41	154	0.02	192	0.06
50	2.58	71	0.32	92	2.40	113	0.04	134	0.25	159	0.05	193	0.27
51	6.98	72	0.03	93	4.22	114	0.01	135	5.90	160	0.03	194	36.29
52	7.98	73	0.07	94	9.52	115	0.42	136	3.58	161	0.33	195	8.09
53	5.24	74	0.35	95	39.38	116	0.24	137	86.09	162	0.13	196	24.41
54	3.45	75	0.71	96	13.57	117	0.47	138	100.00	163	1.63	197	3.16
55	4.57	76	0.54	97	25.19	118	0.22	139	68.09	164	1.01	198	0.30
56	0.53	77	5.39	98	2.63	119	6.55	140	62.07	165	0.76	199	0.02

R4:1618(12:12) m(1595-1630) 143384a-00! -m(1207-1695) 5894 11% 143384a-00 * PE lim: 0.01%

PHIP Experiment: Formation of Carbene 14. [Cp*RuCl]$_4$ (1.9 mg, 4 mol%) was added to a stirred solution of enyne **13** (10.1 mg, 0.04 mmol) in CD_2Cl_2 (1 mL) in a flame dried Schlenk tube under argon. The mixture was transferred into a pressure NMR tube (5 mm medium wall precision pressure/vacuum valve NMR sample tube, *Wilmad-LabGlass*), which was tightly closed. The tube was connected to the *p*-H_2 generator and all tubings were evacuated and backfilled with *para*-hydrogen (*p*-H_2) three times. Then, the pressure was increased to 5 bar and the valve was opened to fill the tube with *para*-hydrogen to a total pressure of ≈ 6 bar. After closing the valve, the tube was shaken and immediately inserted into the NMR magnet.[3]

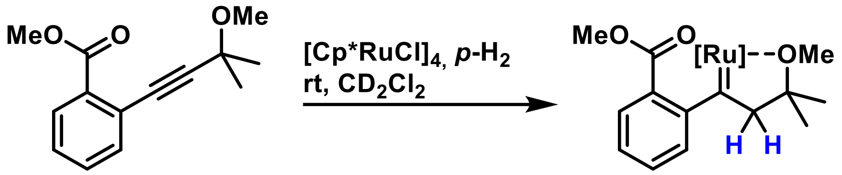

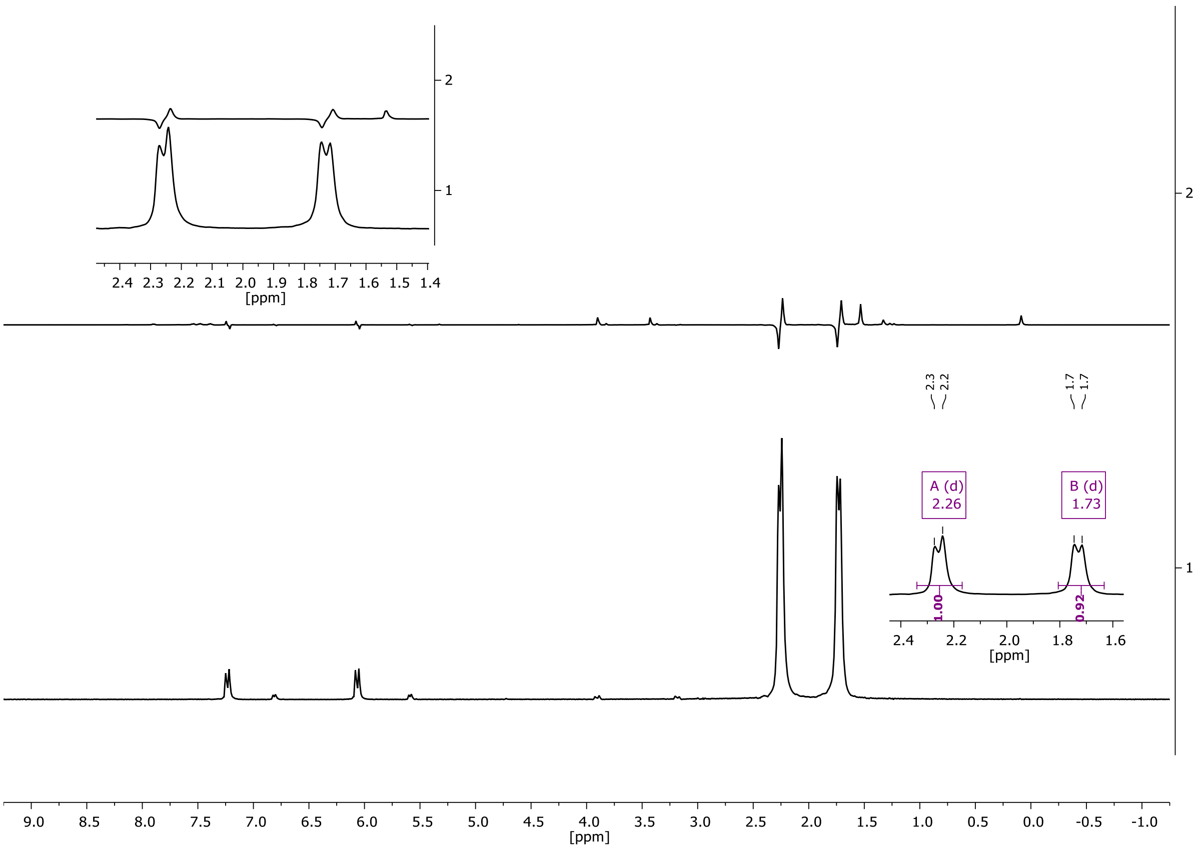

Figure S12. OPSY spectrum recorded upon *gem*-hydrogenation of **13**.

SUBSTRATES

Methyl 2-(((trifluoromethyl)sulfonyl)oxy)cyclohex-1-ene-1-carboxylate (S1). Methyl 2-oxo-cyclohexane-1-carboxylate (900 mg, 5.76 mmol) was added dropwise to a stirred suspension of NaH (166 mg, 6.92 mmol) in CH_2Cl_2 (30 mL) at 0 °C. The mixture was stirred for 30 min at 0 °C before Tf_2O (1.16 mL, 6.92 mmol) was slowly added. Stirring was continued at room temperature for 16 h before water (10 mL) was added. The layers were separated, the aqueous phase was extracted with CH_2Cl_2 (2 x 30 mL) and the combined organic layers were washed with sat. NaCl solution and dried over $MgSO_4$. The solvent was removed under reduced pressure to yield the title compound as a brown oil, which was used without further purification (1.66 g, quant.). ^{1}H NMR (400 MHz, $CDCl_3$) δ 3.80 (s, 3H), 2.48 (m, 2H), 2.40 (m, 2H), 1.84 – 1.74 (m, 2H), 1.67 (m, 2H). ^{13}C NMR (101 MHz, $CDCl_3$) δ 165.3, 151.9, 123.0, 118.5 (q, J = 319.7 Hz), 52.3, 28.7, 26.2, 22.4, 21.1. The spectral data is consistent with those reported in the literature.[4]

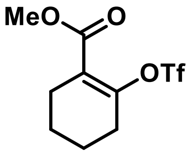

***tert*-Butyldimethyl((2-methylbut-3-yn-2-yl)oxy)silane (S2).** TBSOTf (3.28 mL, 14.3 mmol) was added to a solution of 2-methy-3-butyn-2-ol (1.00 g, 11.9 mmol) and 2,6-lutidine (2.77 mL, 23.8 mmol) in CH_2Cl_2 (32 mL) at 0 °C. The mixture was warmed to room temperature and stirred for 2 h before water (10 mL) and sat. NH_4Cl solution (5 mL) were added. The layers were separated and the aqueous phase was extracted with *tert*-butyl methyl ether (2 x 50 mL). The combined organic layers were washed with sat. NaCl solution and dried over $MgSO_4$. The solvent was removed under reduced pressure and the residue was purified by flash chromatography (silica, hexanes) to yield the title compound as a colorless liquid (1.73 g, 73%). ^{1}H NMR (400 MHz, $CDCl_3$) δ 2.39 (s, 1H), 1.47 (s, 6H), 0.86 (s, 9H), 0.17 (s, 6H). ^{13}C NMR (101 MHz, $CDCl_3$) δ 89.5, 70.7, 66.3, 33.0, 25.8, 18.1, -2.9. IR (film) ṽ 2931, 2858, 1252, 1163, 1040 cm^{-1}.

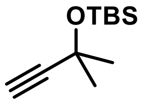

Methyl 2-(3-((*tert*-butyldimethylsilyl)oxy)-3-methylbut-1-yn-1-yl)cyclohex-1-ene-1-carboxylate (1). *i*-Pr_2NEt (0.85 mL, 4.86 mmol), CuI (93 mg, 0.49 mmol) and $Pd(PPh_3)_2Cl_2$ (170 mg, 0.24 mmol) were added to a stirred solution of alkenyl triflate **S1** (700 mg, 2.43 mmol) and alkyne **S2** (963 mg, 4.86 mmol) in DMF (12.2 mL). The mixture was stirred for 1 h at room temperature before sat. NH_4Cl solution (10 mL) and EtOAc (20 mL) were added. The layers were separated and the aqueous phase was extracted with EtOAc (2 x 40 mL). The combined organic layers were washed with sat. NaCl solution (3 x 10 mL) and dried over $MgSO_4$. The solvent was removed under reduced pressure and the residue was purified by flash chromatography (silica, hexanes/EtOAc 1:0 – 50:1– 20:1) to yield the title compound as a pale yellow oil (755 mg, 92%). ^{1}H NMR (400 MHz, $CDCl_3$) δ 3.75 (s, 3H), 2.40 – 2.34 (m, 2H), 2.30 (m, 2H), 1.63 (m, 4H), 1.50 (s, 6H), 0.86 (s, 9H), 0.16 (s, 6H). ^{13}C NMR (101 MHz, $CDCl_3$) δ 168.2, 134.2, 128.0, 101.6, 82.7, 66.9, 51.6, 33.1, 32.1, 26.4, 25.9, 21.9, 21.8, 18.1, -2.9. IR (film) ṽ 2931, 2857, 1727, 1707, 1228, 1160, 1037 cm^{-1}. HRMS (ESI$^+$) for $C_{19}H_{32}O_3Si$ $[M+Na]^+$: calcd 359.2013, found 359.2008.

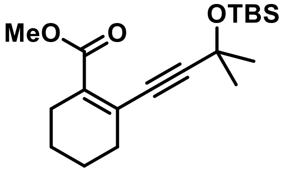

Methyl 2-(3,3-dimethylbut-1-yn-1-yl)cyclohex-1-ene-1-carboxylate (S3). *i*-Pr_2NEt (0.85 mL, 4.86 mmol), CuI (93 mg, 0.49 mmol) and $Pd(PPh_3)_2Cl_2$ (170 mg, 0.24 mmol) were added to a stirred solution of alkenyl triflate **S1** (700 mg, 2.43 mmol) and 3,3-dimethyl-1-butyne (400 mg, 4.86 mmol) in DMF (12.2 mL). The mixture was stirred for 1 h at room temperature before sat. NH_4Cl solution (10 mL) and EtOAc (20 mL) were

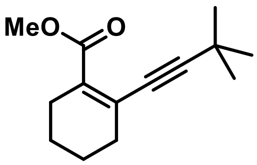

added. The layers were separated and the aqueous phase was extracted with EtOAc (2 x 40 mL). The combined organic layers were washed with sat. NaCl solution (3 x 10 mL) and dried over $MgSO_4$. The solvent was removed under reduced pressure and the residue was purified by flash chromatography (silica, hexanes/EtOAc 1:0 – 40:1– 20:1) to yield the title compound as a pale yellow oil (267 mg, 50%). ^{1}H NMR (400 MHz, $CDCl_3$) δ 3.75 (s, 3H), 2.37 – 2.31 (m, 2H), 2.31 – 2.25 (m, 2H), 1.66 – 1.55 (m, 4H), 1.26 (s, 9H). ^{13}C NMR (101 MHz, $CDCl_3$) δ 168.5, 133.1, 129.0, 105.9, 79.4, 51.4, 32.8, 31.0, 28.4, 26.4, 22.0, 21.9. IR (film) ṽ 2968, 2934, 2215, 1702, 1257, 1227, 1048 cm^{-1}. HRMS (EI$^+$) for $C_{14}H_{20}O_2$ [M]$^+$: calcd 220.1458, found 220.1460.

Methyl 2-(phenylethynyl)cyclohex-1-ene-1-carboxylate (S4). *i*-Pr_2NEt (0.85 mL, 4.86 mmol), CuI (93 mg, 0.49 mmol) and $Pd(PPh_3)_2Cl_2$ (170 mg, 0.24 mmol) were added to a stirred solution of alkenyl triflate **S1** (700 mg, 2.43 mmol) and phenylacetylene (496 mg, 4.86 mmol) in DMF (12.2 mL). The mixture was stirred for 1 h at room temperature before sat. NH_4Cl solution (10 mL) and EtOAc (20 mL) were added. The layers were separated and the aqueous phase was extracted with EtOAc (2 x 40 mL). The combined organic layers were washed with sat. NaCl solution (3 x 10 mL) and dried over $MgSO_4$. The solvent was removed under reduced pressure and the residue was purified by flash chromatography (silica, hexanes/EtOAc 20:1) to yield the title compound as a pale brown oil (413 mg, 71%). ^{1}H NMR (400 MHz, $CDCl_3$) δ 7.86 (ddd, *J* = 7.8, 1.5, 0.6 Hz, 1H), 7.48 (ddd, *J* = 7.8, 1.5, 0.6 Hz, 1H), 7.40 (td, *J* = 7.6, 1.4 Hz, 1H), 7.30 (ddd, *J* = 7.9, 7.4, 1.4 Hz, 1H), 3.92 (s, 3H), 1.34 (s, 9H). ^{13}C NMR (101 MHz, $CDCl_3$) δ 167.5, 134.1, 132.4, 131.5, 130.3, 127.3, 124.3, 103.8, 78.0, 52.1, 31.0, 28.4. IR (film) ṽ 2968, 2867, 2238, 1732, 1715, 1288, 1246, 1079 cm^{-1}. HRMS (EI$^+$) for $C_{14}H_{16}O_2$ [M]$^+$: calcd 216.1145, found 216.1143.

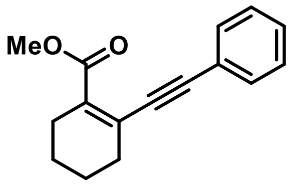

Methyl 2-((trimethylsilyl)ethynyl)cyclohex-1-ene-1-carboxylate (S5). *i*-Pr_2NEt (0.88 mL, 5.02 mmol), CuI (96 mg, 0.50 mmol) and $Pd(PPh_3)_2Cl_2$ (176 mg, 0.25 mmol) were added to a stirred solution of alkenyl triflate **S1** (724 mg, 2.51 mmol) and trimethylsilylacetylene (493 mg, 5.02 mmol) in DMF (12.6 mL). The mixture was stirred for 1 h at room temperature before sat. NH_4Cl solution (10 mL) and EtOAc (20 mL) were added. The layers were separated and the aqueous phase was extracted with EtOAc (2 x 40 mL). The combined organic layers were washed with sat. NaCl solution (3 x 10 mL) and dried over $MgSO_4$. The solvent was removed under reduced pressure and the residue was purified by flash chromatography (silica, hexanes/EtOAc 1:0 – 40:1– 20:1) to yield the title compound as a yellow oil (542 mg, 91%). ^{1}H NMR (400 MHz, CDCl3) δ 3.76 (s, 3H), 2.40 – 2.29 (m, 4H), 1.62 (m, 4H), 0.21 (s, 9H). ^{13}C NMR (101 MHz, CDCl3) δ 168.1, 135.7, 128.0, 104.7, 101.6, 51.6, 32.3, 26.4, 21.8, 21.7, 0.1. The spectral data is consistent with those reported in the literature.[5]

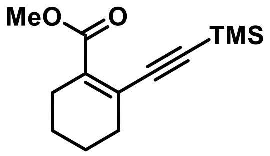

Ethyl 2-(((trifluoromethyl)sulfonyl)oxy)cyclohex-1-ene-1-carboxylate (S6). Ethyl 2-oxocyclohexane-1-carboxylate (400 mg, 2.35 mmol) was added dropwise to a stirred suspension of NaH (68 mg, 2.82 mmol) in CH_2Cl_2 (12 mL) at 0 °C. The mixture was stirred for 30 min at 0 °C before Tf_2O (0.47 mL, 2.82 mmol) was slowly added. The mixture was stirred at room temperature for 16 h before water (5 mL) was added. The layers were separated, the aqueous phase was extracted with CH_2Cl_2 (2 x 20 mL) and the combined organic layers were washed with sat. NaCl solution and dried over $MgSO_4$. The solvent was removed under reduced pressure to yield the title compound as a brown oil, which was used without further purification (525 mg,

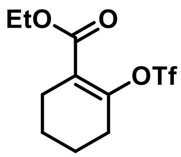

74%). ^{1}H NMR (400 MHz, $CDCl_3$) δ 4.27 (q, *J* = 7.1 Hz, 2H), 2.48 (m, 2H), 2.40 (m, 2H), 1.82 – 1.74 (m, 2H), 1.67 (m, 2H), 1.32 (t, *J* = 7.1 Hz, 3H). ^{13}C NMR (101 MHz, $CDCl_3$) δ 164.9, 151.5, 123.4, 118.5 (q, *J* = 320 Hz), 61.7, 28.6, 26.3, 22.4, 21.2, 14.1. The spectral data is consistent with those reported in the literature.[6]

Ethyl 2-((1-methoxycyclohexyl)ethynyl)cyclohex-1-ene-1-carboxylate (S7). *i*-Pr_2NEt (0.23 mL, 1.32 mmol), CuI (25 mg, 0.13 mmol) and $Pd(PPh_3)_2Cl_2$ (46 mg, 0.07 mmol) were added to a stirred solution of alkenyl triflate **S6** (200 mg, 0.66 mmol) and 1-ethynyl-1-methoxycyclohexane (183 mg, 1.32 mmol)[7] in DMF (3.3 mL). The mixture was stirred for 1 h at room temperature before sat. NH_4Cl solution (3 mL) and EtOAc (10 mL) were added. The layers were separated and the aqueous phase was extracted with EtOAc (2 x 20 mL). The combined organic layers were washed with sat. NaCl solution (3 x 4 mL) and dried over $MgSO_4$. The solvent was removed under reduced pressure and the residue was purified by flash chromatography (silica, hexanes/EtOAc 20:1 – 15:1) to yield the title compound as a light yellow oil (106 mg, 55%). ^{1}H NMR (400 MHz, $CDCl_3$) δ 4.22 (q, *J* = 7.1 Hz, 2H), 3.39 (s, 3H), 2.40 – 2.30 (m, 4H), 1.98 – 1.88 (m, 2H), 1.71 – 1.48 (m, 11H), 1.33 – 1.26 (m, 1H), 1.31 (t, *J* = 7.1 Hz, 3H). ^{13}C NMR (101 MHz, $CDCl_3$) δ 167.7, 134.5, 127.4, 97.6, 86.3, 74.7, 60.7, 51.0, 36.9, 32.7, 26.4, 25.7, 23.0, 21.9, 21.8, 14.4. IR (film) ṽ 2933, 2858, 1719, 1698, 1231, 1089 cm^{-1}. HRMS (EI^+) for $C_{18}H_{26}O_3$ $[M]^+$: calcd 290.1876, found 290.1880.

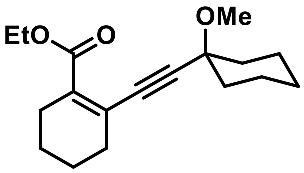

Methyl 2-(3-hydroxy-3-methylbut-1-yn-1-yl)benzoate (S8). To a solution of methyl 2-iodobenzoate (1.78 g, 6.81 mmol) and 2-methy-3-butyn-2-ol (687 mg, 8.17 mmol) in Et_3N (19 mL) was added $Pd(PPh_3)_2Cl_2$ (96 mg, 0.14 mmol), CuI (26 mg, 0.14 mmol) and PPh_3 (71 mg, 0.27 mmol). The mixture was stirred for 12 h at 80 °C before sat. NH_4Cl solution (15 mL) and Et_2O (20 mL) were added. The layers were separated and the aqueous phase was extracted with Et_2O (2 x 20 mL). The combined organic layers were washed with sat. NaCl solution and dried over Na_2SO_4. The solvent was removed under reduced pressure and the residue was purified by flash chromatography (silica, hexanes/EtOAc 10:1 – 5:1) to yield the title compound as a yellow oil (1.12 g, 75%). ^{1}H NMR (400 MHz, $CDCl_3$) δ 7.92 (dd, *J* = 8.2, 1.0 Hz, 1H), 7.51 (dd, *J* = 7.7, 1.4 Hz, 1H), 7.45 (td, *J* = 7.0, 1.4 Hz, 1H), 7.35 (td, *J* = 7.4, 1.4 Hz, 1H), 3.92 (s, 3H), 2.26 – 2.08 (br, 1H), 1.64 (s, 6H). ^{13}C NMR (101 MHz, $CDCl_3$) δ 166.8, 134.0, 132.2, 131.8, 130.5, 128.1, 123.3, 99.0, 81.1, 65.8, 53.6, 52.3, 31.4. The spectral data is consistent with those reported in the literature.[8]

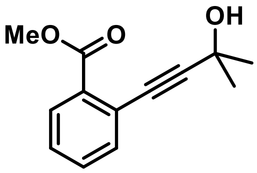

Methyl 2-(3-methoxy-3-methylbut-1-yn-1-yl)benzoate (13). Propargylic alcohol **S8** (500 mg, 2.29 mmol) was added slowly to a stirred suspension of NaH (110 mg, 4.58 mmol) in THF (9.3 mL) at 0 °C. The mixture was stirred for 10 min at room temperature before MeI (0.72 mL, 11.5 mmol) was added dropwise. After stirring for 30 min the reaction was quenched with water (2 mL) and EtOAc (10 mL). The layers were separated and the aqueous phase was extracted with EtOAc (2 x 30 mL). The combined organic layers were washed with sat. NaCl solution and dried over $MgSO_4$. The solvent was removed under reduced pressure and the residue was purified by flash chromatography (silica, hexanes/EtOAc 10:1 – 5:1) to yield the title compound as a colorless oil (291 mg, 55%). ^{1}H NMR (400 MHz, $CDCl_3$) δ 7.91 (ddd, *J* = 7.8, 1.4, 0.5 Hz, 1H), 7.54 (ddd, *J* = 7.8, 1.4, 0.6 Hz, 1H), 7.45 (td, *J* = 7.6, 1.5 Hz, 1H), 7.36 (ddd, *J* = 7.9, 7.4, 1.4 Hz, 1H), 3.92 (s, 3H), 3.47 (s, 3H), 1.57 (s, 6H). ^{13}C NMR (101 MHz, $CDCl_3$) δ 166.9,

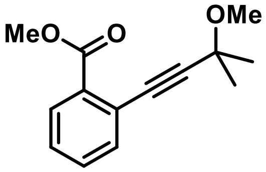

134.23, 132.3, 131.7, 130.5, 128.0, 123.3, 96.3, 83.1, 71.2, 52.3, 52.0, 28.4. IR (film) ṽ 2984, 2949, 1730, 1717, 1250, 1073 cm^{-1}. HRMS (CI^+) for $C_{14}H_{16}O_3$ $[M+H]^+$: calcd 255.0992, found 255.0988.

Methyl (*Z*)-6-((*tert*-butyldimethylsilyl)oxy)-6-methylhept-2-en-4-ynoate (S9). *i*-Pr_2NEt (0.37 mL, 2.12 mmol), CuI (41 mg, 0.21 mmol) and $Pd(PPh_3)_2Cl_2$ (77 mg, 0.11 mmol) were added to a stirred solution of methyl (*Z*)-3-iodoacrylate (225 mg, 1.06 mmol) and alkyne **S2** (422 mg, 2.13 mmol) in DMF (5.4 mL). The mixture was stirred for 1 h at room temperature before sat. NH_4Cl solution (5 mL) and EtOAc (10 mL) were added. The layers were separated and the aqueous phase was extracted with EtOAc (2 x 20 mL). The combined organic layers were washed with sat. NaCl solution (3 x 5 mL) and dried over $MgSO_4$. The solvent was removed under reduced pressure and the residue was purified by flash chromatography (silica, hexanes/EtOAc 1:0 – 20:1) to yield the title compound as a light yellow oil (244 mg, 81%). ^{1}H NMR (400 MHz, $CDCl_3$) δ 6.15 (d, *J* = 11.5 Hz, 1H), 6.06 (d, *J* = 11.5 Hz, 1H), 3.76 (s, 3H), 1.54 (s, 6H), 0.87 (s, 9H), 0.16 (s, 6H). ^{13}C NMR (101 MHz, $CDCl_3$) δ 165.2, 128.1, 122.9, 107.2, 79.6, 67.1, 51.6, 32.8, 25.8, 18.1, –3.0. IR (film) ṽ 2954, 2930, 2857, 1733, 1611, 1159, 1034 cm^{-1}. HRMS (ESI^+) for $C_{15}H_{26}O_3Si$ $[M+Na]^+$: calcd 305.1543, found 305.1540.

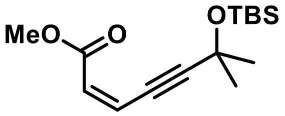

1-(*tert*-Butyl) 3-methyl 4-(3-((*tert*-butyldimethylsilyl)oxy)-3-methylbut-1-yn-1-yl)-5,6-dihydropyridine-1,3(2H)-dicarboxylate (S10). *i*-Pr_2NEt (0.37 mL, 2.12 mmol), CuI (41 mg, 0.21 mmol) and $Pd(PPh_3)_2Cl_2$ (77 mg, 0.11 mmol) were added to a stirred solution of the corresponding piperidyl triflate (414 mg, 1.06 mmol)[9] and alkyne **S2** (422 mg, 2.13 mmol) in DMF (5.4 mL). The mixture was stirred for 1 h at room temperature before sat. NH_4Cl solution (5 mL) and EtOAc (10 mL) were added. The layers were separated and the aqueous phase was extracted with EtOAc (2 x 20 mL). The combined organic layers were washed with sat. NaCl solution (3 x 5 mL) and dried over $MgSO_4$. The solvent was removed under reduced pressure and the residue was purified by flash chromatography (silica, hexanes/EtOAc 20:1 – 10:1) to yield the title compound as a pale brown oil (353 mg, 76%). ^{1}H NMR (400 MHz, $CDCl_3$) δ 4.19 (t, *J* = 2.6 Hz, 2H), 3.77 (s, 3H), 3.48 (t, *J* = 5.8 Hz, 2H), 2.40 (m, 2H), 1.52 (s, 6H), 1.47 (s, 9H), 0.86 (s, 9H), 0.15 (s, 6H). ^{13}C NMR (101 MHz, $CDCl_3$) δ 165.4 (br), 154.5, 130.4 (br), 128.4 (br), 104.2, 81.6, 80.4 (br), 67.0, 51.7, 43.8 (br), 39.0 (br), 32.9, 31.9 (br), 28.6, 25.8, 18.1, –2.8. IR (film) ṽ 2931, 2856, 1732, 1699, 1238, 1159, 1035 cm^{-1}. HRMS (ESI^+) for $C_{23}H_{39}NO_5Si$ $[M+Na]^+$: calcd 460.2490, found 460.2484.

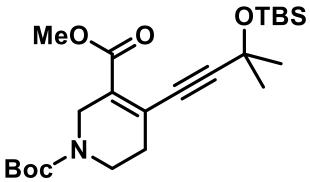

2-(3,3-Dimethylbut-1-yn-1-yl)cyclohex-1-ene-1-carbaldehyde (S11). *i*-Pr_2NEt (0.52 mL, 2.96 mmol), CuI (56 mg, 0.30 mmol) and $Pd(PPh_3)_2Cl_2$ (104 mg, 0.15 mmol) were added to a stirred solution of 2-bromocyclohex-1-ene-1-carbaldehyde (280 mg, 1.48 mmol)[10] and 3,3-dimethyl-1-butyne (243 mg, 2.96 mmol) in DMF (7.5 mL). The mixture was stirred for 1 h at room temperature before sat. NH_4Cl solution (7 mL) and EtOAc (15 mL) were introduced. The layers were separated and the aqueous phase was extracted with EtOAc (2 x 30 mL). The combined organic layers were washed with sat. NaCl solution (3 x 5 mL) and dried over $MgSO_4$. The solvent was removed under reduced pressure and the residue was purified by flash chromatography (silica, hexanes/EtOAc 1:0 – 40:1) to yield the title compound as a colorless oil (249 mg, 88%). ^{1}H NMR (400 MHz, $CDCl_3$) δ 10.17 (s, 1H), 2.37 (m, 2H), 2.22 (m, 2H), 1.69 – 1.59 (m, 4H), 1.27 (s,

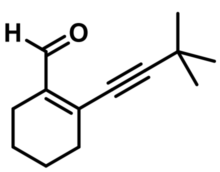

9H). ^{13}C NMR (101 MHz, $CDCl_3$) δ 193.6, 141.6, 141.3, 108.7, 76.6, 32.9, 30.9, 28.5, 22.0 (2C), 21.3. IR (film) ṽ 2968, 2932, 2214, 1673, 1601, 1225 cm^{-1}. HRMS (EI^+) for $C_{13}H_{18}O$ $[M]^+$: calcd 190.1352, found 190.1351.

1-(2-(3,3-Dimethylbut-1-yn-1-yl)cyclohex-1-en-1-yl)ethan-1-ol (S12). MeLi (1.6 M in Et_2O, 0.58 mL, 0.93 mmol) was added to a stirred solution of **S11** (147 mg, 0.77 mmol) in Et_2O (4.0 mL) at −78 °C. The mixture was stirred for 1 h at −78 °C before sat. NH_4Cl solution (2 mL) was introduced and the mixture warmed to room temperature. The layers were separated and the organic phase was extracted with EtOAc (3 x 10 mL). The combined organic layers were washed with sat. NaCl solution and dried over $MgSO_4$. The solvent was removed under reduced pressure and the crude product was purified by flash chromatography (silica, hexanes/EtOAc 20:1 – 10:1) to give the title product as a colorless oil (143 mg, 90%). ^1H NMR (400 MHz, $CDCl_3$) δ 4.92 (q, *J* = 6.5 Hz, 1H), 2.24 – 1.98 (m, 4H), 1.81 (s, br, 1H), 1.68 – 1.49 (m, 4H), 1.27 (d, *J* = 6.5 Hz, 3H), 1.25 (s, 9H). ^{13}C NMR (101 MHz, $CDCl_3$) δ 146.0, 115.7, 102.7, 78.3, 69.7, 31.3, 30.8, 28.2, 23.4, 22.6, 22.4, 20.7. IR (film) ṽ 3326 (br), 2967, 2927, 1450, 1361, 1265, 1055 cm^{-1}. HRMS (EI^+) for $C_{14}H_{22}O$ $[M]^+$: calcd 206.1665, found 206.1666.

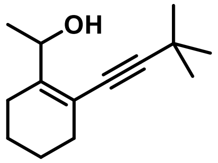

1-(2-(3,3-Dimethylbut-1-yn-1-yl)cyclohex-1-en-1-yl)ethan-1-one (S13). $NaHCO_3$ (274 mg, 3.3 mmol) and Dess-Martin periodinane (333 mg, 0.9 mmol) were added to a solution of alcohol **S12** (135 mg, 0.65 mmol) in wet CH_2Cl_2 (6 mL) at 0 °C. The mixture was stirred for 1 h before sat. $Na_2S_2O_3$ solution (2 mL) and water (2 mL) were introduced. The layers were separated and the aqueous phase was extracted with CH_2Cl_2 (2 x 10 mL). The combined organic layers were washed with sat. NaCl solution and dried over $MgSO_4$. The solvent was removed under reduced pressure and the residue was purified by flash chromatography (silica, hexanes/EtOAc 20:1) to give the title compound as a colorless oil (106 mg, 79%). ^1H NMR (400 MHz, $CDCl_3$) δ 2.55 (s, 3H), 2.36 – 2.26 (m, 4H), 1.60 (m, 4H), 1.26 (s, 9H). ^{13}C NMR (101 MHz, $CDCl_3$) δ 201.7, 142.1, 128.9, 108.3, 80.6, 33.5, 30.9, 30.8, 28.5, 25.6, 22.2, 21.9. IR (film) ṽ 2968, 2932, 2863, 2212, 1656, 1362, 1243 cm^{-1}. HRMS (EI^+) for $C_{14}H_{20}O$ $[M]^+$: calcd 204.1509, found 204.1509.

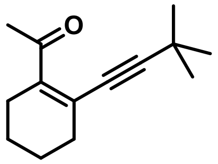

Dimethyl 2-(3-(1-methoxycyclohexyl)prop-2-yn-1-ylidene)malonate (S14). *n*-BuLi (1.6 M in hexanes, 1.85 mL, 2.95 mmol) was added dropwise to a stirred solution of 1-ethynyl-1-methoxycyclohexane (340 mg, 2.46 mmol)[7] in THF (8.0 mL) at −78 °C. The mixture was stirred for 10 min at that temperature before DMF (0.46 mL, 5.90 mmol) was introduced and stirring was continued for 30 min. 10% aq. KH_2PO_4 solution (10 mL) was added and the mixture was warmed to 0 °C and stirred for 30 min. *tert*-Butyl methyl ether (20 mL) was added, the layers were separated and the aqueous phase was extracted with *tert*-butyl methyl ether (2 x 20 mL). The combined organic layers were washed with sat. NaCl solution and dried over $MgSO_4$. The solvent was removed under reduced pressure to yield the crude propargylic aldehyde, which was directly used in the subsequent step.

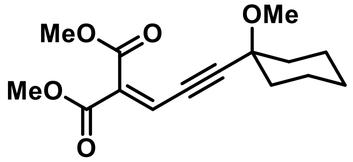

The crude aldehyde was added to a mixture of HOAc (0.07 mL, 1.23 mmol), piperidine (49 µL, 0.49 mmol) and dimethyl malonate (0.24 mL, 2.09 mmol) in toluene (2.1 mL). $MgSO_4$ (120 mg, 0.98 mmol) was introduced and the mixture stirred at 60 °C for 1 h before the reaction was quenched with water (3 mL). The layers were separated and the organic phase was extracted with EtOAc (3 x 20 mL). The combined organic layers were washed with sat. NaCl solution and dried over $MgSO_4$. The solvent was removed under reduced pressure and the residue was purified by flash chromatography (silica, hexanes/EtOAc 10:1 – 6:1)

to yield the title compound as a pale yellow oil (152 mg, 22% over two steps). ^{1}H NMR (400 MHz, $CDCl_3$) δ 6.96 (s, 1H), 3.85 (s, 3H), 3.81 (s, 3H), 3.35 (s, 3H), 1.94 (m, 2H), 1.74 – 1.42 (m, 7H), 1.36 – 1.23 (m, 1H). ^{13}C NMR (101 MHz, $CDCl_3$) δ 164.8, 163.7, 135.1, 125.4, 107.4, 81.3, 74.8, 52.9, 52.7, 51.2, 36.5, 25.4, 22.8. IR (film) ṽ 2936, 2858, 2200, 1725, 1608, 1436, 1254, 1071 cm^{-1}. HRMS (ESI$^+$) for $C_{15}H_{20}O_5$ [M+Na]$^+$: calcd 303.1203, found 303.1200.

Methyl (*Z*)-5-(1-(methoxymethoxy)cyclohexyl)pent-2-en-4-ynoate (S15). *i*-Pr_2NEt (0.49 mL, 2.83 mmol),

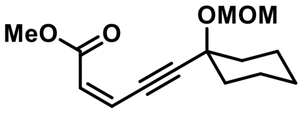

CuI (54 mg, 0.28 mmol) and $Pd(PPh_3)_2Cl_2$ (99 mg, 0.14 mmol) were added to a stirred solution of methyl (*Z*)-3-iodoacrylate (300 mg, 1.42 mmol) and 1-ethynyl-1-(methoxymethoxy)cyclohexane (422 mg, 2.13 mmol) in DMF (7.1 mL). The mixture was stirred for 1 h at room temperature before sat. NH_4Cl solution (5 mL) and EtOAc (10 mL) were added. The layers were separated and the aqueous layer was extracted with EtOAc (2 x 20 mL). The combined extracts were washed with sat. NaCl solution (3 x 5 mL) and dried over $MgSO_4$. The solvent was removed under reduced pressure and the residue was purified by flash chromatography (silica, hexanes/EtOAc 20:1 – 10:1) to yield the title compound as a light yellow oil (249 mg, 70%). ^{1}H NMR (400 MHz, $CDCl_3$) δ 6.20 (d, *J* = 11.6 Hz, 1H), 6.09 (d, *J* = 11.5 Hz, 1H), 5.01 (s, 2H), 3.76 (s, 3H), 3.41 (s, 3H), 2.03 (m, 2H), 1.76 – 1.52 (m, 7H), 1.35 – 1.22 (m, 1H). ^{13}C NMR (101 MHz, $CDCl_3$) δ 165.1, 128.4, 122.7, 102.9, 93.2, 83.6, 75.6, 56.0, 51.7, 38.6, 25.5, 23.1. IR (film) ṽ 2935, 2210, 1728, 1611, 1438, 1172, 1021 cm^{-1}. HRMS (ESI$^+$) for $C_{14}H_{20}O_4$ [M+Na]$^+$: calcd 275.1254, found 275.1252.

3-(3-(1-Methoxycyclohexyl)prop-2-yn-1-ylidene)pentane-2,4-dione (15). *n*-BuLi (1.6 M in hexanes,

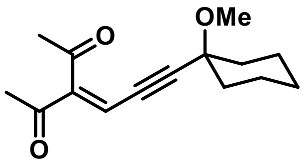

2.71 mL, 4.34 mmol) was added dropwise to a stirred solution of 1-ethynyl-1-methoxycyclohexane (500 mg, 3.62 mmol)[7] in THF (11.7 mL) at −78 °C. The mixture was stirred for 10 min at that temperature before DMF (0.67 mL, 8.68 mmol) was added and stirring was continued for 30 min. 10% aq. KH_2PO_4 solution (10 mL) was introduced and the mixture was warmed to 0 °C and stirred for 30 min. *tert*-Butyl methyl ether (20 mL) was added, the layers were separated and the aqueous phase was extracted with *tert*-butyl methyl ether (2 x 30 mL). The combined organic layers were washed with sat. NaCl solution and dried over $MgSO_4$. The solvent was removed under reduced pressure to yield the crude propargylic aldehyde which was directly used in the subsequent step.

The crude aldehyde was added to a mixture of HOAc (0.10 mL, 1.80 mmol), piperidine (36 µL, 0.36 mmol) and acetylacetone (0.32 mL, 3.07 mmol) in toluene (3.1 mL). $MgSO_4$ (87 mg, 0.72 mmol) was added and the mixture was stirred at 30 °C for 1 h before water (3 mL) was introduced. The layers were separated and the organic layer was extracted with *tert*-butyl methyl ether (3 x 20 mL). The combined organic layers were washed with sat. NaCl solution and dried over $MgSO_4$. The solvent was removed under reduced pressure and the residue was purified by flash chromatography (silica, hexanes/EtOAc 5:2 – 4:1) to yield the title compound as a light yellow oil (486 mg, 54% over two steps). ^{1}H NMR (400 MHz, $CDCl_3$) δ 6.73 (s, 1H), 3.34 (s, 3H), 2.47 (s, 3H), 2.33 (s, 3H), 1.96 – 1.86 (m, 2H), 1.73 – 1.41 (m, 7H), 1.38 – 1.26 (m, 1H). ^{13}C NMR (101 MHz, $CDCl_3$) δ 201.1, 195.7, 150.5, 121.7, 109.3, 81.4, 74.7, 51.3, 36.4, 31.1, 27.3, 25.4, 22.7. IR (film) ṽ 2936, 2858, 2208, 1666, 1247, 1090 cm^{-1}. HRMS (ESI$^+$) for $C_{15}H_{20}O_3$ [M+Na]$^+$: calcd 271.1305, found 271.1301.

3-(4,4-Dimethylpent-2-yn-1-ylidene)pentane-2,4-dione (21a). *n*-BuLi (1.6 M in hexanes, 3.74 mL, 6.00 mmol) was added dropwise to a stirred solution of 3,3-dimethyl-but-1-yne (410 mg, 4.99 mmol) in Et_2O (16.8 mL) at −78 °C. The mixture was stirred for 10 min at that temperature before DMF (0.93 mL, 12.0 mmol) was introduced and stirring was continued for 30 min. 10% aq. KH_2PO_4 solution (15 mL) was added and the mixture was warmed to 0 °C and stirred for 30 min. The mixture was diluted with Et_2O (30 mL), the layers were separated and the aqueous phase was extracted with Et_2O (2 x 30 mL). The combined organic layers were washed with sat. NaCl solution and dried over $MgSO_4$. The solvent was removed under reduced pressure to yield the crude propargylic aldehyde, which was directly used in the subsequent step.

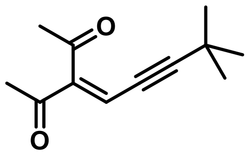

The crude aldehyde was added to a mixture of HOAc (0.14 mL, 2.49 mmol), piperidine (49 µL, 0.49 mmol) and acetylacetone (0.44 mL, 4.24 mmol) in toluene (4.3 mL). $MgSO_4$ (120 mg, 1.00 mmol) was introduced and the mixture was stirred at 40 °C for 1 h before water (3 mL) was added. The layers were separated and the aqueous phase was extracted with *tert*-butyl methyl ether (3 x 30 mL). The combined organic layers were washed with sat. NaCl solution and dried over $MgSO_4$. The solvent was removed under reduced pressure and the residue was purified by flash chromatography (silica, hexanes/EtOAc 20:1 – 10:1) to yield the title compound as a pale yellow oil (277 mg, 29% over two steps). ^{1}H NMR (400 MHz, $CDCl_3$) δ 6.71 (s, 1H), 2.48 (s, 3H), 2.30 (s, 3H), 1.27 (s, 9H). ^{13}C NMR (101 MHz, $CDCl_3$) δ 201.4, 195.9, 149.4, 123.5, 118.1, 75.9, 31.0, 130.4, 29.0, 27.5. IR (film) ṽ 2971, 2220, 2195, 1691, 1664, 1362, 1245, 1225 cm^{-1}. HRMS (CI$^+$) for $C_{12}H_{16}O_2$ [M+H]$^+$: calcd 193.1223, found 193.1224.

3-(*tert*-Butyldimethylsilyl)propiolaldehyde (S16). *n*-BuLi (1.6 M in hexanes, 6.24 mL, 9.98 mmol) was added dropwise to a stirred solution of (*tert*-butyldimethylsilyl)acetylene (1.40 g, 9.98 mmol) in THF (10.5 mL) at 0 °C. The mixture was stirred for 30 min at that temperature before DMF (3.09 mL, 39.9 mmol) was introduced and stirring was continued for 30 min. sat. NH_4Cl solution (30 mL) was added and the mixture was stirred for 30 min. *tert*-butyl methyl ether (60 mL) was added, the layers were separated and the aqueous phase was extracted with *tert*-butyl methyl ether (2 x 70 mL). The combined organic layers were washed with sat. NaCl solution and dried over $MgSO_4$. The solvent was removed under reduced pressure and the residue was purified by flash chromatography (silica, hexanes/EtOAc 1:0 – 20:1) to yield the title compound as a pale yellow oil (1.54 g, 92%). ^{1}H NMR (400 MHz, $CDCl_3$) δ 9.18 (s, 1H), 0.97 (s, 9H), 0.20 (s, 6H). ^{13}C NMR (101 MHz, $CDCl_3$) δ 176.8, 103.2, 102.1, 26.1, 16.7, –5.1. The spectral data is consistent with those reported in the literature.[11]

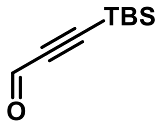

3-(3-(*tert*-Butyldimethylsilyl)prop-2-yn-1-ylidene)pentane-2,4-dione (21b). Aldehyde **S16** (500 mg, 2.97 mmol) was added to a mixture of HOAc (0.09 mL, 1.49 mmol), piperidine (29 µL, 0.30 mmol) and acetylacetone (0.27 mL, 2.67 mmol) in toluene (2.5 mL). $MgSO_4$ (72 mg, 0.59 mmol) was introduced and the suspension was stirred at ambient temperature for 1 h before water (2 mL) was added. The layers were separated and the organic phase was extracted with *tert*-butyl methyl ether (3 x 20 mL). The combined organic layers were washed with sat. NaCl solution and dried over $MgSO_4$. The solvent was removed under reduced pressure and the residue was purified by flash chromatography (silica, hexanes/EtOAc 20:1 – 10:1) to yield the title compound as a pale yellow oil (572 mg, 77%). ^{1}H NMR (400 MHz, $CDCl_3$) δ 6.67 (s, 1H), 2.50 (s, 3H), 2.31 (s, 3H), 0.94 (s, 9H), 0.15 (s, 6H). ^{13}C NMR (101 MHz, $CDCl_3$) δ 201.1, 195.7, 150.9, 121.7,

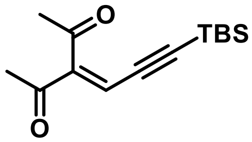

113.8, 100.4, 31.1, 27.5, 26.1, 16.8, -4.9. IR (film) ṽ 2954, 2930, 2858, 1668, 1362, 1248 cm^{-1}. HRMS (CI^+) for $C_{14}H_{22}O_2Si$ $[M+H]^+$: calcd 251.1462, found 251.1460.

3-(3-Phenylprop-2-yn-1-ylidene)pentane-2,4-dione (21c). *n*-BuLi (1.6 M in hexanes, 3.06 mL, 4.90 mmol) was added dropwise to a stirred solution of phenylacetylene (500 mg, 4.90 mmol) in THF (15.9 mL) at −78 °C. The mixture was stirred for 10 min at that temperature before DMF (0.91 mL, 11.7 mmol) was introduced and stirring was continued for 30 min. 10% aq. KH_2PO_4 solution (10 mL) was added and the mixture was warmed to 0 °C and stirred for 30 min. The mixture was diluted with *tert*-butyl methyl ether (20 mL), the layers were separated and the aqueous phase was extracted with *tert*-butyl methyl ether (2 x 30 mL). The combined organic layers were washed with sat. NaCl solution and dried over $MgSO_4$. The solvent was removed under reduced pressure to yield the crude propargylic aldehyde, which was directly used in the subsequent step.

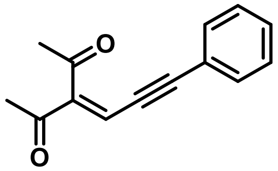

The crude aldehyde was added to a mixture of HOAc (0.14 mL, 2.45 mmol), piperidine (48 µL, 0.49 mmol) and acetylacetone (0.42 mL, 4.16 mmol) in toluene (4.2 mL). $MgSO_4$ (118 mg, 0.98 mmol) was introduced and the suspension was stirred at 40 °C for 1 h before water (3 mL) was added. The layers were separated and the organic phase was extracted with *tert*-butyl methyl ether (3 x 20 mL). The combined organic layers were washed with sat. NaCl solution and dried over $MgSO_4$. The solvent was removed under reduced pressure and the residue was purified by flash chromatography (silica, hexanes/EtOAc 20:1 – 10:1) to yield the title compound as a pale yellow oil (513 mg, 49% over two steps). ^1H NMR (400 MHz, $CDCl_3$) δ 7.50 – 7.45 (m, 2H), 7.44 – 7.34 (m, 3H), 6.94 (s, 1H), 2.57 (s, 3H), 2.37 (s, 3H). ^{13}C NMR (101 MHz, $CDCl_3$) δ 201.0, 195.7, 149.5, 132.3, 130.3, 128.8, 122.4, 121.7, 107.2, 85.4, 31.2, 27.6. IR (film) ṽ 3032, 2191, 1662, 1572, 1243, 1175 cm^{-1}. HRMS (ESI^+) for $C_{14}H_{12}O_2$ $[M+Na]^+$: calcd 235.0729, found 235.0727.

3-(Hept-2-yn-1-ylidene)pentane-2,4-dione (21d). *n*-BuLi (1.6 M in hexanes, 3.74 mL, 6.00 mmol) was added dropwise to a stirred solution of 1-hexyne (410 mg, 4.99 mmol) in Et_2O (16.8 mL) at −78 °C. The mixture was stirred for 10 min at that temperature before DMF (0.93 mL, 12.0 mmol) was introduced and stirring was continued for 30 min. 10% aq. KH_2PO_4 solution (15 mL) was added and the mixture was warmed to 0 °C and stirred for 30 min. The mixture was diluted with Et_2O (30 mL), the layers were separated and the aqueous phase was extracted with Et_2O (2 x 30 mL). The combined organic layers were washed with sat. NaCl solution and dried over $MgSO_4$. The solvent was removed under reduced pressure to yield the crude propargylic aldehyde, which was directly used in the subsequent step.

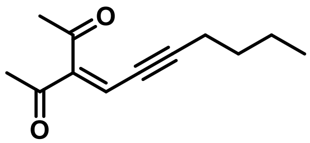

The crude aldehyde was added to a mixture of HOAc (0.14 mL, 2.49 mmol), piperidine (49 µL, 0.49 mmol) and acetylacetone (0.44 mL, 4.24 mmol) in toluene (4.3 mL). $MgSO_4$ (120 mg, 1.00 mmol) was introduced and the mixture was stirred at 40 °C for 1 h before water (3 mL) was added. The layers were separated and the aqueous phase was extracted with *tert*-butyl methyl ether (3 x 30 mL). The combined organic layers were washed with sat. NaCl solution and dried over $MgSO_4$. The solvent was removed under reduced pressure and the residue was purified by flash chromatography (silica, hexanes/EtOAc 20:1 – 10:1) to yield the title compound as a pale yellow oil (403 mg, 42% over two steps). ^1H NMR (400 MHz, $CDCl_3$) δ 6.69 (t, J = 2.5 Hz, 1H), 2.46 (s, 3H), 2.43 (td, J = 7.0, 2.5 Hz, 2H), 2.31 (s, 3H), 1.58 – 1.51 (m, 2H), 1.48 – 1.34 (m, 2H), 0.92 (t, J = 7.3 Hz, 3H). ^{13}C NMR (101 MHz, $CDCl_3$) δ 201.51, 195.94, 149.65, 123.42, 110.63, 76.98,

31.08, 30.25, 27.36, 22.10, 20.05, 13.63. IR (film) ṽ 2959, 2934, 2209, 1689, 1663, 1375, 1247 cm^{-1}. HRMS (CI^+) for $C_{12}H_{16}O_2$ $[M+H]^+$: calcd 193.1223, found 193.1223.

FURANS AND BUTENOLIDES

Representative Procedure A. Preparation of 3-((1-methoxycyclohexyl)methyl)-4,5,6,7-tetrahydroisobenzofuran-1(3*H*)-one (9).

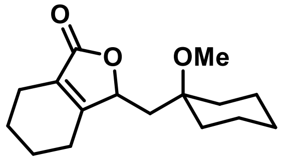

$[Cp^*RuCl]_4$ (2.2 mg, 2 mol%) was added to a stirred solution of enyne **S7** (29.7 mg, 0.10 mmol) in CH_2Cl_2 (1 mL) in a flame dried Schlenk tube under argon. H_2 was bubbled through the solution for 2 min before the mixture was stirred for 3 h at room temperature under ambient pressure of H_2 (balloon). The solvent was removed under reduced pressure and the residue was loaded onto a prepared silica column and left for 1 h to ensure complete hydrolysis of the furan initially formed. The product was then eluted with hexanes/EtOAc (4:1 – 2:1) to give butenolide **8** as a colorless oil (23.9 mg, 88%). Single crystals suitable for X-ray analysis were obtained by layering a saturated Et_2O solution with pentane at 0 °C. ^1H NMR (400 MHz, $CDCl_3$) δ 4.98 – 4.92 (m, 1H), 3.17 (s, 3H), 2.35 – 2.15 (m, 4H), 1.92 (dd, *J* = 15.5, 1.6 Hz, 1H), 1.88 – 1.39 (m, 13H), 1.36 – 1.22 (m, 2H). ^{13}C NMR (101 MHz, $CDCl_3$) δ 173.9, 164.3, 126.4, 79.5, 74.5, 48.3, 39.3, 34.8, 33.9, 25.7, 23.3, 22.1, 21.9, 21.8, 21.8, 20.1. IR (film) ṽ 2926, 2857, 1738, 1677, 1064, 1031 cm^{-1}. HRMS (ESI^+) for $C_{16}H_{24}O_3$ $[M+Na]^+$: calcd 287.1618, found 287.1617.

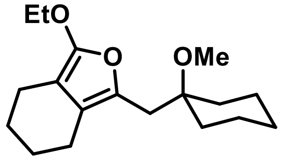

The 2-ethoxyfuran primarily formed can be obtained in reasonable purity (≈ 95%) upon filtration of the crude product through Florisil. It analyzed as follows: ^1H NMR (400 MHz, $CDCl_3$) δ 4.11 (q, *J* = 7.1 Hz, 2H), 3.25 (s, 3H), 2.55 (s, 2H), 2.37 (m, 4H), 1.75 – 1.68 (m, 2H), 1.66 – 1.40 (m, 11H), 1.32 (t, *J* = 7.0 Hz, 3H), 1.23 – 1.08 (m, 1H). ^{13}C NMR (101 MHz, $CDCl_3$) δ 152.5, 136.3, 119.3, 96.8, 76.4, 68.3, 48.7, 34.4, 33.9, 25.8, 23.6, 23.4, 21.9, 21.5, 19.6, 15.4.

3-(2-((*tert*-Butyldimethylsilyl)oxy)-2-methylpropyl)-4,5,6,7-tetrahydroisobenzofuran-1(3*H*)-one (3).

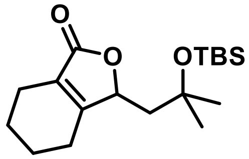

Prepared according to the Representative Procedure A as a colorless solid (27.9 mg, 80 %). ^1H NMR (400 MHz, $CDCl_3$) δ 5.10 – 5.03 (m, 1H), 2.33 – 2.11 (m, 4H), 1.90 (d, *J* = 14.5 Hz, 1H), 1.84 – 1.60 (m, 4H), 1.40 (dd, *J* = 14.6, 9.5 Hz, 1H), 1.39 (s, 3H), 1.30 (s, 3H), 0.86 (s, 9H), 0.11 (s, 3H), 0.11 (s, 3H). ^{13}C NMR (101 MHz, $CDCl_3$) δ 174.1, 164.7, 126.2, 80.5, 72.7, 47.8, 32.0, 29.0, 26.0, 23.3, 21.8, 20.1, 18.2, –1.9, –2.0. IR (film) ṽ 2935, 2852, 1744, 1679, 1034 cm^{-1}. HRMS (ESI^+) for $C_{18}H_{32}O_3Si$ $[M+Na]^+$: calcd 347.2013, found 347.2008.

3-Allyl-3-(2-((*tert*-butyldimethylsilyl)oxy)-2-methylpropyl)-4,5,6,7-tetrahydroisobenzofuran-1(3*H*)-one (4).

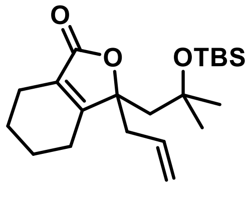

Prepared according to the Representative Procedure A from enyne **1** (32.7 mg, 0.10 mmol); the crude product was filtered through a pad of Florisil and the filtrate was evaporated. A thick-walled Schlenk tube was charged with the resulting crude furan **2,** NaI (2.9 mg, 0.02 mmol), allyl iodide (36 µL, 0.39 mmol) and THF (0.7 mL) The tube was sealed and the resulting mixture stirred at 100 °C for 16 h before it was cooled to ambient temperature. The solvent was removed under reduced pressure and the residue

was purified by flash chromatography (silica, hexanes/EtOAc 20:1 – 10:1) to give the title compound as a colorless oil (19.4 mg, 55%). ^{1}H NMR (400 MHz, $CDCl_3$) δ 5.43 (dddd, *J* = 16.8, 10.1, 7.9, 6.5 Hz, 1H), 5.07 – 4.98 (m, 2H), 2.92 (ddt, *J* = 14.7, 6.5, 1.3 Hz, 1H), 2.34 (ddt, *J* = 14.7, 7.9, 1.0 Hz, 1H), 2.30 – 2.10 (m, 4H), 2.07 (d, *J* = 14.9 Hz, 1H), 1.76 – 1.57 (m, 5H), 1.38 (s, 3H), 1.19 (s, 3H), 0.86 (s, 9H), 0.10 (s, 3H), 0.08 (s, 3H). ^{13}C NMR (101 MHz, $CDCl_3$) δ 173.2, 166.5, 131.2, 126.9, 119.0, 89.2, 74.1, 50.4, 41.0, 32.0, 30.2, 26.1, 23.1, 21.7, 21.7, 20.0, 18.2, -1.6, -1.7. IR (film) ṽ 2930, 2856, 1749, 1462, 1253, 1026 cm^{-1}. HRMS (ESI$^+$) for $C_{21}H_{36}O_3Si$ $[M+Na]^+$: calcd 387.2326, found 387.2321.

***tert*-Butyl((1-(5-methoxyfuran-2-yl)-2-methylpropan-2-yl)oxy)dimethylsilane (5).** Prepared according to the Representative Procedure A; the crude product was immediately subjected to chromatographic purification to avoid hydrolysis; colorless oil (27.5 mg, 83%). ^{1}H NMR (400 MHz, $CDCl_3$) δ 5.89 (dt, *J* = 3.1, 0.7 Hz, 1H), 5.02 (d, *J* = 3.1 Hz, 1H), 3.80 (s, 3H), 2.62 (s, 2H), 1.22 (s, 6H), 0.85 (s, 9H), 0.05 (s, 6H). ^{13}C NMR (101 MHz, $CDCl_3$) δ 160.6, 143.7, 108.4, 79.8, 73.6, 57.8, 43.7, 29.7, 25.9, 18.2, -2.0. IR (film) ṽ 2930, 2856, 1616, 1586, 1259, 1042 cm^{-1}. HRMS (ESI$^+$) for $C_{15}H_{28}O_3Si$ $[M+Na]^+$: calcd 307.1700, found 307.1697.

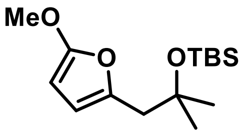

2-Methoxy-5-((1-(methoxymethoxy)cyclohexyl)methyl)furan (6). According to the Representative Procedure A as a colorless oil (25.5 mg, 71%). ^{1}H NMR (400 MHz, $CDCl_3$) δ 5.90 (dt, *J* = 3.1, 0.7 Hz, 1H), 5.02 (d, *J* = 3.1 Hz, 1H), 4.76 (s, 2H), 3.79 (s, 3H), 3.41 (s, 3H), 2.73 (d, *J* = 0.7 Hz, 2H), 1.81 – 1.71 (m, 2H), 1.67 – 1.36 (m, 7H), 1.29 – 1.17 (m, 1H). ^{13}C NMR (101 MHz, $CDCl_3$) δ 160.7, 142.4, 108.8, 91.0, 79.8, 77.3, 57.7, 55.8, 37.7, 34.8, 25.7, 22.2. IR (film) ṽ 2931, 2860, 1738, 1615, 1586, 1261, 1029 cm^{-1}. HRMS (ESI$^+$) for $C_{14}H_{22}O_4$ $[M+Na]^+$: calcd 277.1410, found 277.1408.

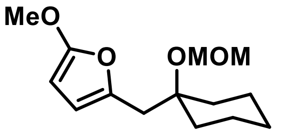

Methyl 2-methoxy-5-((1-methoxycyclohexyl)methyl)furan-3-carboxylate (7). Prepared according to the Representative Procedure A as a colorless oil (11.0 mg, 66 %). ^{1}H NMR (400 MHz, $CDCl_3$) δ 6.26 (t, *J* = 0.7 Hz, 1H), 4.07 (s, 3H), 3.77 (s, 3H), 3.24 (s, 3H), 2.64 (d, *J* = 0.8 Hz, 2H), 1.78 – 1.64 (m, 2H), 1.61 – 1.38 (m, 5H), 1.35 – 1.14 (m, 3H). ^{13}C NMR (101 MHz, $CDCl_3$) δ 163.7, 161.3, 142.2, 109.1, 91.7, 75.1, 58.0, 51.3, 48.6, 35.1, 34.0, 25.8, 21.9. IR (film) ṽ 2932, 2856, 1705, 1601, 1079 cm^{-1}. HRMS (ESI$^+$) for $C_{15}H_{22}O_5$ $[M+H]^+$: calcd 283.1540, found 283.1538.

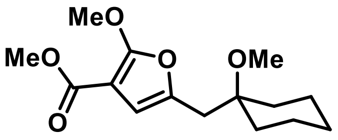

***tert*-Butyl 1-(2-((*tert*-butyldimethylsilyl)oxy)-2-methylpropyl)-3-oxo-1,4,6,7-tetrahydrofuro[3,4-c]-pyridine-5(3*H*)-carboxylate (8).** Prepared according to the Representative Procedure A as a pale yellow oil (29.2 mg, 65%). ^{1}H NMR (400 MHz, $CDCl_3$) δ 5.17 (d, *J* = 9.3 Hz, 1H), 4.19 (br, 1H), 4.06 (dq, *J* = 17.9, 2.8 Hz, 1H), 3.76 (dt, *J* = 13.4, 5.3 Hz, 1H), 3.53 (br, 1H), 2.49 – 2.36 (br, 1H), 2.34 – 2.25 (br, 1H), 1.90 (d, *J* = 14.4 Hz, 1H), 1.45 (dd, *J* = 14.6, 9.5 Hz, 1H), 1.47 (s, 9H), 1.39 (s, 3H), 1.30 (s, 3H), 0.85 (s, 9H), 0.11 (s, 3H), 0.10 (s, 3H). ^{13}C NMR (101 MHz, $CDCl_3$) δ 171.5, 163.6 (br), 154.8, 124.4, 80.7, 80.6, 72.5, 47.6, 40.2 (br), 39.2 (br), 31.9, 28.9, 28.5, 26.0, 23.7, 18.2, −2.0, −2.0. IR (film) ṽ 2930, 2857, 1754, 1698, 1414, 1162, 1036 cm^{-1}. HRMS (ESI$^+$) for $C_{22}H_{39}NO_5Si$ $[M+Na]^+$: calcd 448.2490, found 448.2489.

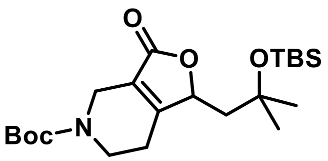

3-Neopentyl-4,5,6,7-tetrahydroisobenzofuran-1(3*H*)-one (10). Prepared according to the Representative Procedure A as a pale yellow oil (19.4 mg, 66 %). ^{1}H NMR (400 MHz, $CDCl_3$) δ 4.86 – 4.79 (m, 1H), 2.31 – 2.11 (m, 4H), 1.85 – 1.65 (m, 4H), 1.62 (dd, *J* = 14.7, 1.8 Hz, 1H), 1.28 (dd, *J* = 14.7, 10.4 Hz, 1H), 1.02 (s, 9H). ^{13}C NMR (101 MHz, $CDCl_3$) δ 174.0, 164.6, 126.2, 81.2, 46.5, 30.5, 30.0, 23.3, 21.8, 21.8, 20.0. IR (film) ṽ 2939, 2865, 1743, 1679, 1028 cm^{-1}. HRMS (EI$^+$) for $C_{13}H_{20}O_2$ [M]$^+$: calcd 208.1458, found 208.1455.

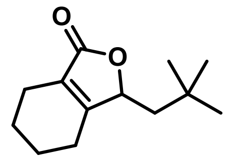

3-((Trimethylsilyl)methyl)-4,5,6,7-tetrahydroisobenzofuran-1(3*H*)-one (11). Prepared according to the Representative Procedure A as a colorless solid (17.1 mg, 76%). ^{1}H NMR (400 MHz, $CDCl_3$) δ 4.90 – 4.82 (m, 1H), 2.33 – 2.11 (m, 4H), 1.80 – 1.60 (m, 4H), 1.10 (dd, *J* = 14.7, 3.9 Hz, 1H), 0.71 (dd, *J* = 14.8, 10.7 Hz, 1H), 0.10 (s, 9H). ^{13}C NMR (101 MHz, $CDCl_3$) δ 173.7, 166.2, 125.3, 81.6, 23.0, 21.8, 21.8, 20.8, 20.0, –0.7. IR (film) ṽ 2946, 1737, 1674, 1316, 1242, 1030 cm^{-1}. HRMS (EI$^+$) for $C_{12}H_{20}O_2Si$ [M]$^+$: calcd 224.1227, found 224.1223.

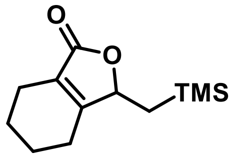

Methyl (*E*)-2-styrylcyclohex-1-ene-1-carboxylate (12). Prepared according to the Representative Procedure A from enyne **S4** (28.4 mg, 0.12 mmol) as a colorless oil (16.2 mg, 57 %). ^{1}H NMR (400 MHz, $CDCl_3$) δ 7.78 (d, *J* = 16.2 Hz, 1H), 7.50 – 7.43 (m, 2H), 7.36 – 7.30 (m, 2H), 7.30 – 7.20 (m, 1H), 6.76 (d, *J* = 16.3 Hz, 1H), 3.81 (s, 3H), 2.57 – 2.39 (m, 4H), 1.78 – 1.64 (m, 4H). ^{13}C NMR (101 MHz, $CDCl_3$) δ 169.6, 141.9, 137.6, 130.4, 128.7 (2C), 128.0, 127.9, 127.0, 51.7, 27.7, 26.5, 22.3, 22.1. IR (film) ṽ 3022, 2932, 1703, 1432, 1234, 1218, 1177, 1048 cm^{-1}. HRMS (EI$^+$) for $C_{16}H_{18}O_2$ [M]$^+$: calcd 242.1301, found 242.1302.

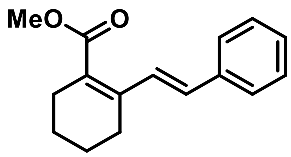

Representative Procedure B. Preparation of 1-(2-Methyl-5-neopentylfuran-3-yl)ethan-1-one (23a). [Cp*RuCl]$_4$ (3.7 mg, 2 mol%) was added to a stirred solution of enyne **20a** (32.9 mg, 0.17 mmol) in 1,2-dichloroethane (1.7 mL) in a flame dried Schlenk tube under argon. H_2 was bubbled through the solution for 2 min before the mixture was stirrred for 3 h at 70 °C under ambient pressure of H_2 (balloon). The solvent was removed under reduced pressure and the residue was purified by flash chromatography (silica, hexanes/EtOAc 20:1 – 10:1) to afford the title product as a colorless oil (24.0 mg, 72%). ^{1}H NMR (400 MHz, $CDCl_3$) δ 6.21 (s, 1H), 2.54 (s, 3H), 2.43 (s, 2H), 2.38 (s, 3H), 0.93 (s, 9H). ^{13}C NMR (101 MHz, $CDCl_3$) δ 194.6, 157.0, 152.8, 122.2, 107.6, 42.0, 31.6, 29.5, 29.3, 14.6. IR (film) ṽ 2954, 2869, 1675, 1568, 1232 cm^{-1}. HRMS (EI$^+$) for $C_{12}H_{18}O_2$ [M]$^+$: calcd 194.1301, found 194.1301.

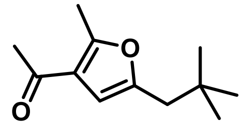

[D$_2$]-22a. Prepared as described above by running the reaction under H_2/D_2 (1:1) atmosphere (prepared by manually mixing H_2 and D_2 in a 10-mL-Hamilton syringe) The resulting product (19.8 mg, 59 %) contained **23a** (58.6%) and **[D$_2$]-23a** (40.7%) Analytical data of **[D$_2$]-23a**: ^{1}H NMR (500 MHz, $CDCl_3$) δ 6.21 (s, 1H), 2.54 (s, 3H), 2.38 (s, 3H), 0.93 (s, 9H). ^{2}H NMR (77 MHz, $CDCl_3$) δ 2.37. ^{13}C NMR (126 MHz, $CDCl_3$) δ 194.4, 156.9, 152.6, 122.0, 107.5, 41.1 (quint., *J* = 19.5 Hz), 31.27, 29.28, 29.12, 14.41. HRMS (EI$^+$) for $C_{12}H_{16}D_2O_2$ [M]$^+$: calcd 196.1427, found 196.1427.

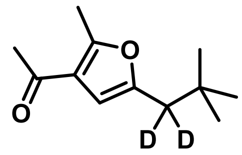

1-(5-((1-Methoxycyclohexyl)methyl)-2-methylfuran-3-yl)ethan-1-one (16). Prepared according to the Representative Procedure B as a pale yellow oil (16.4 mg, 57 %). ^{1}H NMR (400 MHz, $CDCl_3$) δ 6.28 (s, 1H), 3.25 (s, 3H), 2.72 – 2.69 (s, 2H), 2.54 (s, 3H), 2.37 (s, 3H), 1.78 – 1.70 (m, 2H), 1.61 – 1.40 (m, 5H), 1.35 – 1.16 (m, 3H). ^{13}C NMR (101 MHz, $CDCl_3$) δ 194.4, 157.2, 150.4, 122.4, 108.2, 75.1, 48.6, 35.1, 34.0, 29.3, 25.8, 21.9, 14.6. IR (film) ṽ 2931, 2857, 1674, 1567, 1230, 1073 cm^{-1}. HRMS (CI$^+$) for $C_{15}H_{22}O_3$ [M+H]$^+$: calcd 251.1641, found 251.1643.

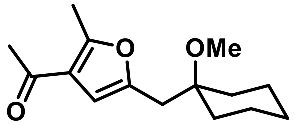

1-(5-((*tert*-Butyldimethylsilyl)methyl)-2-methylfuran-3-yl)ethan-1-one (23b). Prepared according to the Representative Procedure B as a colorless oil (18.2 mg, 64 %). ^{1}H NMR (400 MHz, $CDCl_3$) δ 6.02 (s, 1H), 2.52 (s, 3H), 2.35 (s, 3H), 2.01 (s, 2H), 0.88 (s, 9H), –0.01 (s, 6H). ^{13}C NMR (101 MHz, $CDCl_3$) δ 194.5, 156.1, 152.8, 122.5, 104.2, 29.3, 26.5, 16.8, 14.5, 14.2, –5.9. IR (film) ṽ 2953, 2928, 2857, 1676, 1569, 1233 cm^{-1}. HRMS (EI$^+$) for $C_{14}H_{24}O_2Si$ [M]$^+$: calcd 252.1540, found 252.1539.

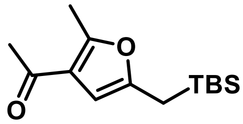

1-Methyl-3-neopentyl-4,5,6,7-tetrahydroisobenzofuran (24). Prepared according to the Representative Procedure B from enyne **S13** (25.7 mg, 0.13 mmol) as a colorless oil (17.0 mg, 66%). ^{1}H NMR (400 MHz, $CDCl_3$) δ 2.38 (dd, *J* = 7.5, 3.6 Hz, 4H), 2.34 (s, 2H), 2.14 (t, *J* = 1.1 Hz, 3H), 1.65 (m, 4H), 0.93 (s, 9H). ^{13}C NMR (101 MHz, $CDCl_3$) δ 146.3, 143.3, 118.0, 116.0, 40.6, 33.1, 29.8, 23.8, 23.7, 21.3, 20.6, 11.8. IR (film) ṽ 2930, 2861, 1444, 1364, 1242 cm^{-1}. HRMS (EI$^+$) for $C_{14}H_{22}O$ [M]$^+$: calcd 206.1665, found 206.1667.

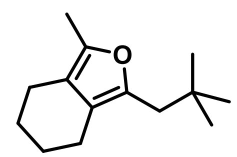

1-(5-Benzyl-2-methylfuran-3-yl)ethan-1-one (23c) and 1-(5-Benzyl-2-methylfuran-3-yl)ethan-1-one (23c, R = Ph). H_2 was bubbled through a stirred mixture of [Cp*RuCl]$_4$ (2.0 mg, 2 mol%) in 1,2-dichloroethane (5 mL) at room temperature for 2 min before immersing the Schlenk tube in a pre-heated oil bath (70 °C). A solution of enyne **21c** (19.5 mg, 0.09 mmol) in 1,2-dichloroethane (0.8 mL) was added over 4 h via syring pump to the catalyst solution which was stirred under an atmosphere of H_2 (balloon). After reaction times of 80 min and 160 min, additional [Cp*RuCl]$_4$ (2.0 mg, 2 mol% in 0.5 mL 1,2-dichloroethane each) was added via syringe. The solvent was removed under reduced pressure and the residue was purified by flash chromatography (silica, hexanes/EtOAc 20:1 – 10:1) to give the product as a colorless oil (9.2 mg, 47%). ^{1}H NMR (400 MHz, $CDCl_3$) δ 7.36 – 7.29 (m, 2H), 7.29 – 7.21 (m, 3H), 6.20 (s, 1H), 3.91 (s, 2H), 2.54 (s, 3H), 2.35 (s, 3H). ^{13}C NMR (101 MHz, $CDCl_3$) δ 194.4, 157.7, 152.7, 137.5, 128.9, 128.8, 126.9, 122.3, 107.0, 34.4, 29.3, 14.6. IR (film) ṽ 3029, 2919, 1673, 1566, 1229 cm^{-1}. HRMS (EI$^+$) for $C_{14}H_{14}O_2$ [M]$^+$: calcd 214.0988, found 214.0988.

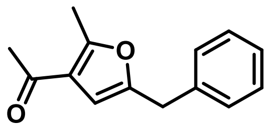

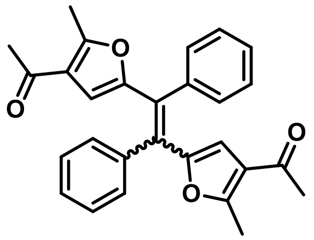

When the reaction is carried out at higher concentration, alkene **26** (R = Ph) becomes the major product, see Table S5. Purification by flash chromatography gave pure isomers, which analyzed as follows: (*E*)-**26** (R = Ph): yellow solid (4.4 mg, 19%,); ^{1}H NMR (400 MHz, $CDCl_3$) δ 7.39 – 7.34 (m, 6H), 7.34 – 7.28 (m, 4H), 5.91 (s, 2H), 2.20 (s, 12H). ^{13}C NMR (101 MHz, $CDCl_3$) δ 194.2, 158.1, 152.6, 140.2, 130.3, 128.6, 128.3, 127.8, 122.8, 113.0, 29.1, 14.3. The spectral data is consistent with those reported in the literature.[12]

(*Z*)-**26** (R = Ph): pale yellow oil (4.3 mg, 19%); ^{1}H NMR (400 MHz, $CDCl_3$) δ 7.18 – 7.11 (m, 10H), 6.29 (s, 2H), 2.48 (s, 6H), 2.34 (s, 6H). ^{13}C NMR (101 MHz, $CDCl_3$) δ 194.3, 157.9, 153.4, 139.8, 131.5, 129.6, 128.0, 127.7, 122.9, 112.6, 29.3, 14.6. The spectral data is consistent with those reported in the literature.[12]

Table S5. Concentration-dependence of the product distribution in the hydrogenative cyclization of enyne **21c** with formation of products **23c** and **26** (R = Ph)

#	[cat.]	c (initial)	substrate solution	addition over:	temp.	conversion	23c/26-ratio (crude NMR)	isolated yield (23c)
1	2 %	0.1 M	neat	instant	70 °C	full	0.04	
2	2 %	0.1 M	0.1 M	2 h	70 °C	full	0.7	25%
3	2 %	0.03 M	0.1 M	4 h	70 °C	45 %	1.3	19%
4	3 %	0.03 M	0.1 M	14 h	rt	10 %	1.0	
5	4 %	0.03 M	0.1 M	2 h	70 °C	90 %	0.9	
6	2+2 %	0.03 M	0.1 M	4 h	70 °C	90 %	1.9	38%
7	2+2+2 %	0.02 M	0.13 M	4 h	70 °C	full	10.0	47%

(*E*)-1,1'-(Dec-5-ene-5,6-diylbis(2-methylfuran-5,3-diyl))bis(ethan-1-one) (26, R = *n*-Bu)). Prepared according to the Representative Procedure A but under Ar atmosphere from enyne **21d** (26.1 mg, 0.14 mmol); the crude product was immediately purified by flash chromatography to give the title compound as a colorless oil (15.5 mg, 59 %). Singe crystals suitable for X-ray analysis were obtained by slow evaporation of a dilute solution in Et_2O/pentane (1:1, *v/v*). m.p.: 92-93 °C. ^{1}H NMR (400 MHz, $CDCl_3$) δ 6.53 (s, 2H), 2.62 (s, 6H), 2.58 – 2.49 (m, 4H), 2.43 (s, 6H), 1.50 – 1.40 (m, 4H), 1.40 – 1.29 (m, 4H), 0.91 (t, *J* = 7.2 Hz, 6H). ^{13}C NMR (101 MHz, $CDCl_3$) δ 194.2, 157.5, 152.5, 129.3, 122.8, 109.3, 33.0, 31.9, 29.3, 22.9, 14.7, 14.1. IR (film) ṽ 2919, 2854, 1680, 1578, 1398, 1232 cm^{-1}. HRMS (ESI$^+$) for $C_{24}H_{32}O_4$ $[M+H]^+$: calcd 385.2373, found 385.2271.

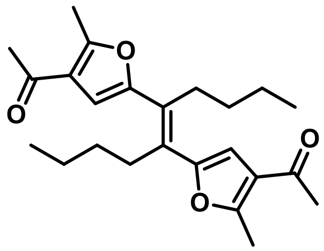

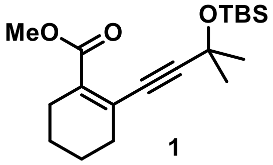
MeO
O
OTBS

1

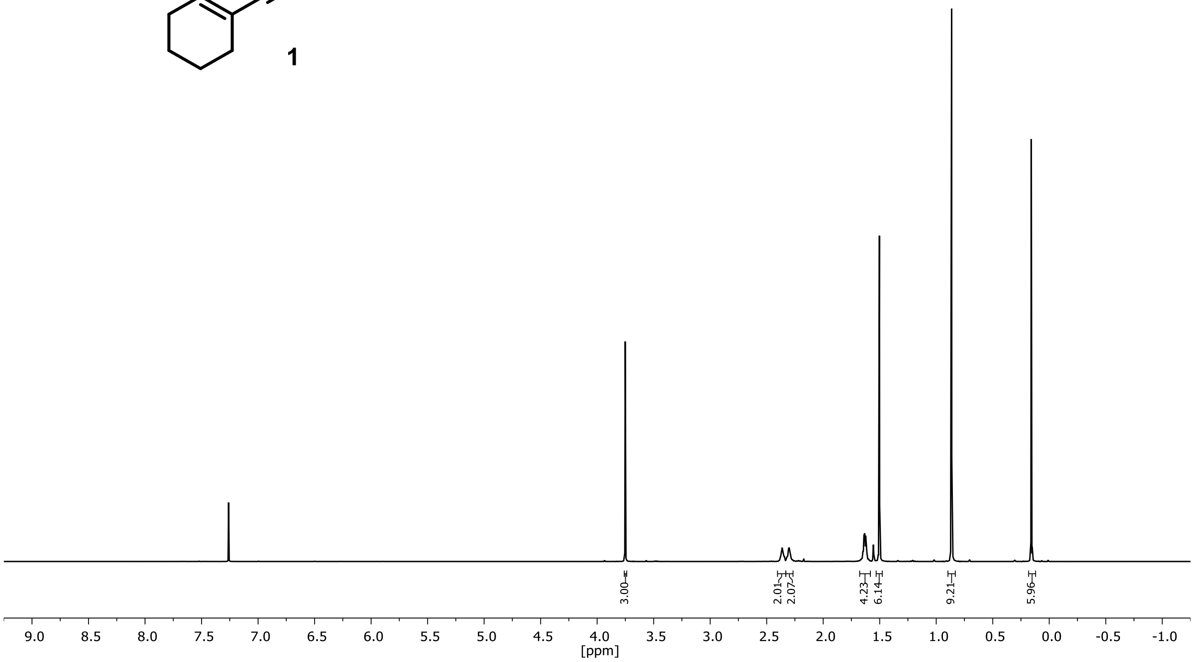
3.00
2.01
2.07
4.23
6.14
9.21
5.96
9.0 8.5 8.0 7.5 7.0 6.5 6.0 5.5 5.0 4.5 4.0 3.5 3.0 2.5 2.0 1.5 1.0 0.5 0.0 -0.5 -1.0
[ppm]

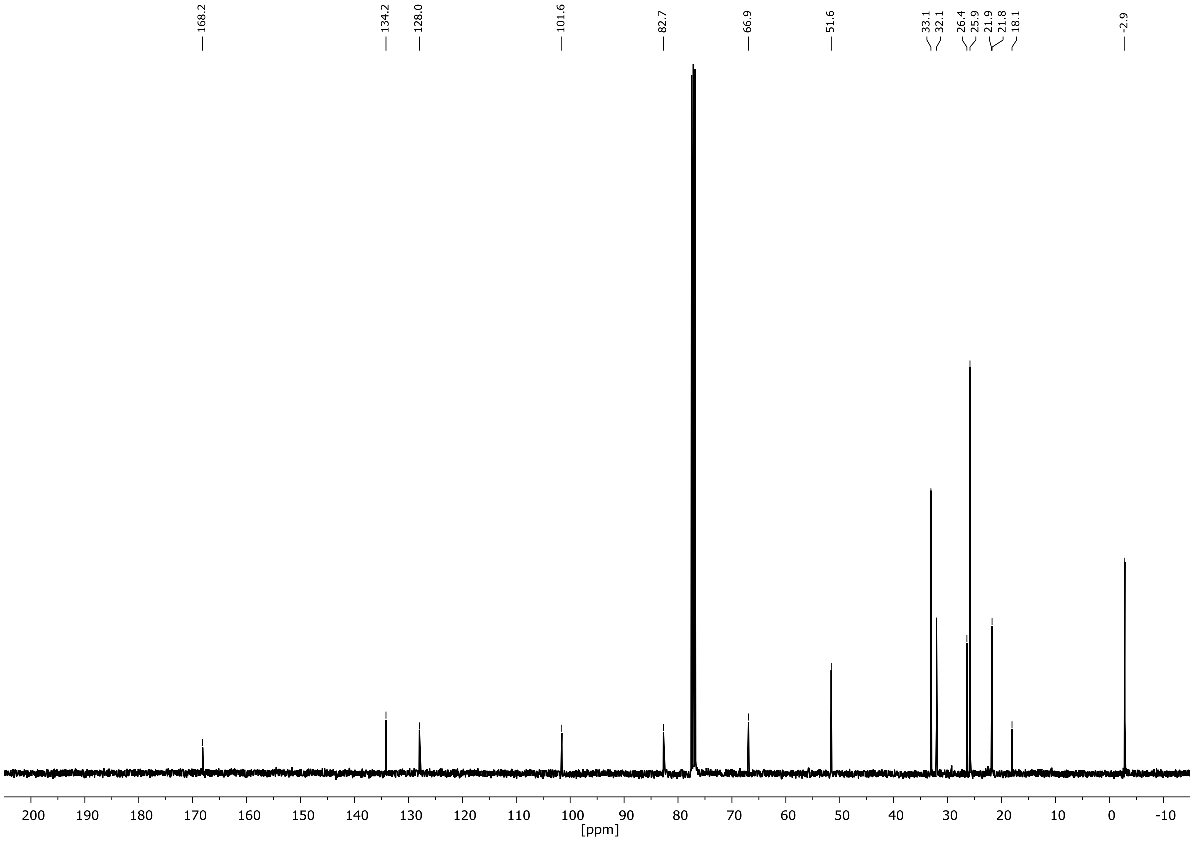
168.2
134.2
128.0
101.6
82.7
66.9
51.6
33.1
32.1
26.4
25.9
21.9
21.8
18.1
-2.9
200 190 180 170 160 150 140 130 120 110 100 90 80 70 60 50 40 30 20 10 0 -10
[ppm]

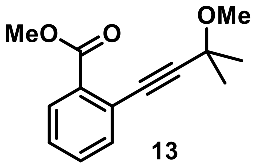
MeO
O
OMe
13

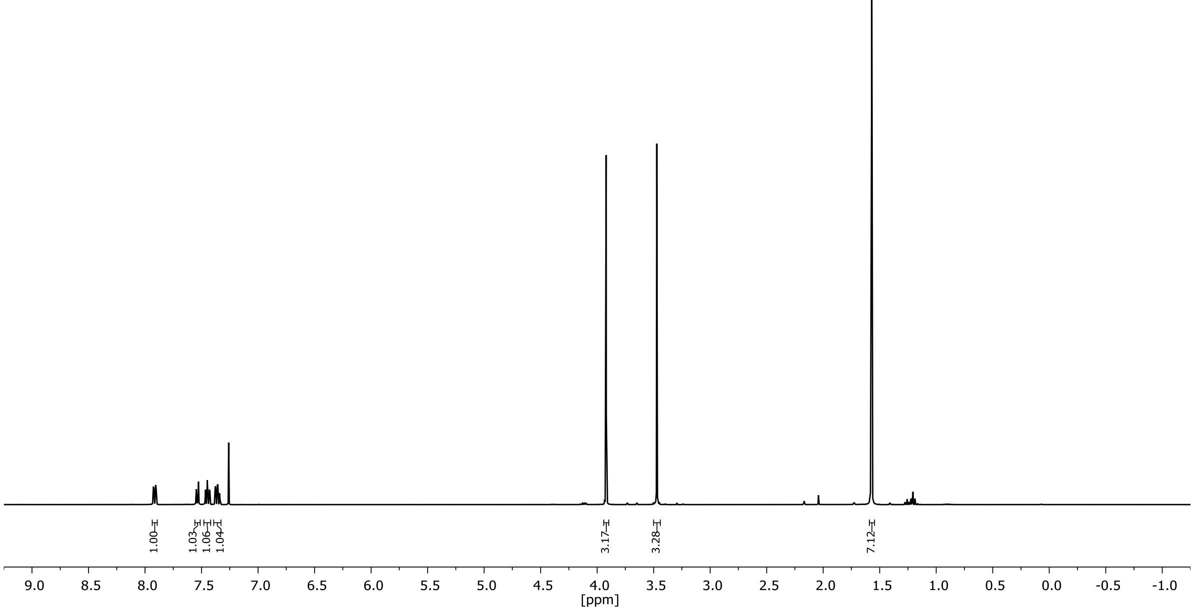
1.00
1.03
1.06
1.04
3.17
3.28
7.12
9.0 8.5 8.0 7.5 7.0 6.5 6.0 5.5 5.0 4.5 4.0 3.5 3.0 2.5 2.0 1.5 1.0 0.5 0.0 -0.5 -1.0
[ppm]

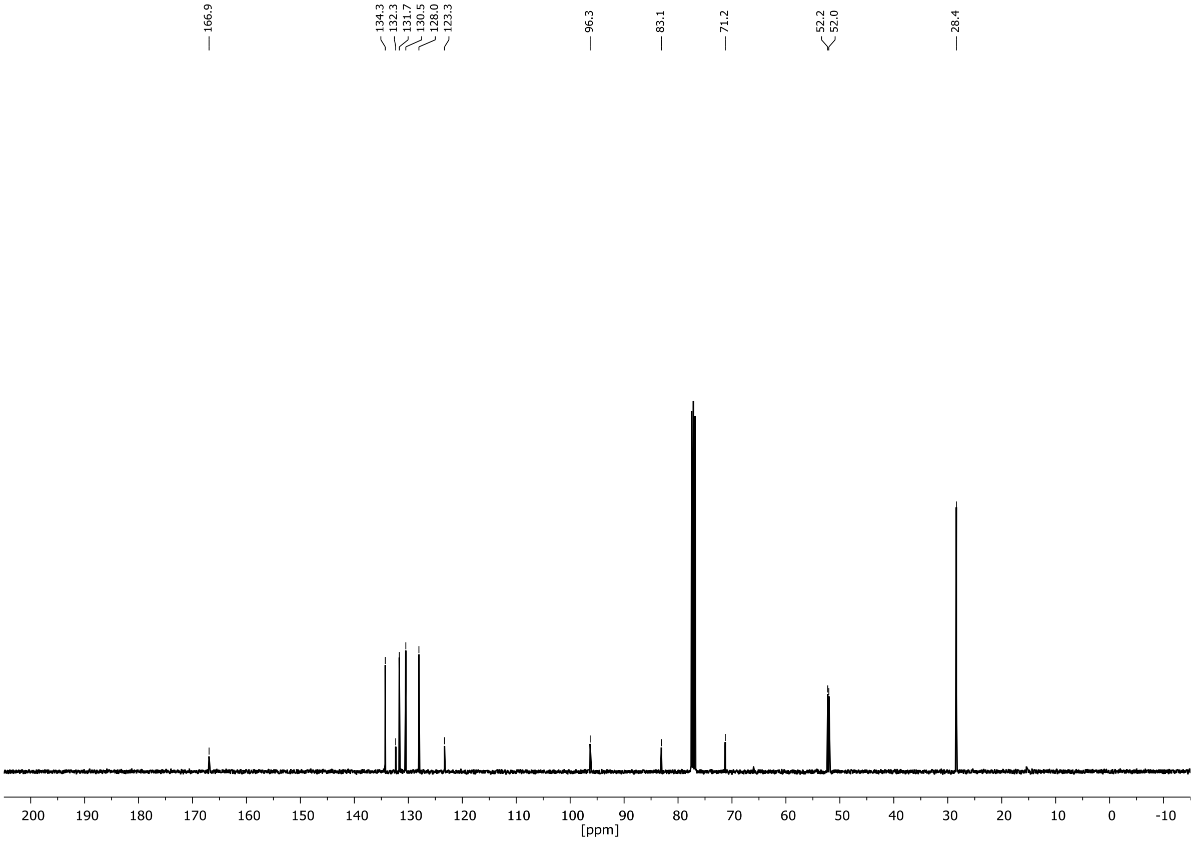
166.9
134.3
132.3
131.7
130.5
128.0
123.3
96.3
83.1
71.2
52.2
52.0
28.4
200 190 180 170 160 150 140 130 120 110 100 90 80 70 60 50 40 30 20 10 0 -10
[ppm]

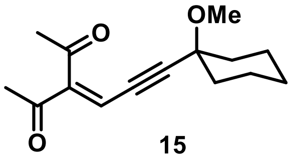
OMe
O
O

15

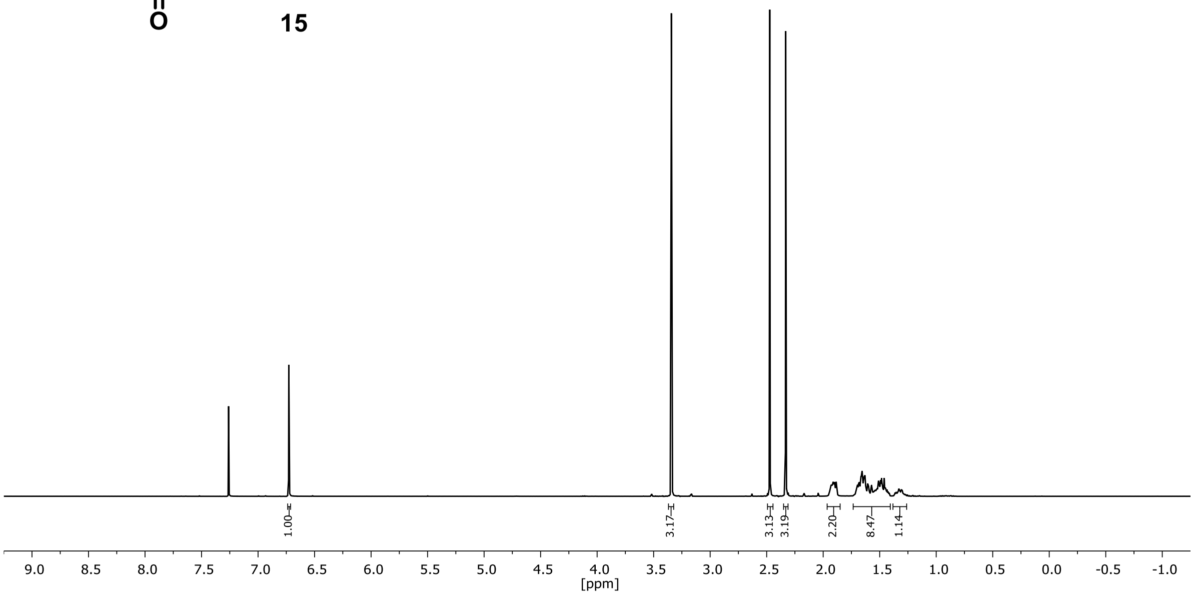
1.00
3.17
3.13
3.19
2.20
8.47
1.14
9.0 8.5 8.0 7.5 7.0 6.5 6.0 5.5 5.0 4.5 4.0 3.5 3.0 2.5 2.0 1.5 1.0 0.5 0.0 -0.5 -1.0
[ppm]

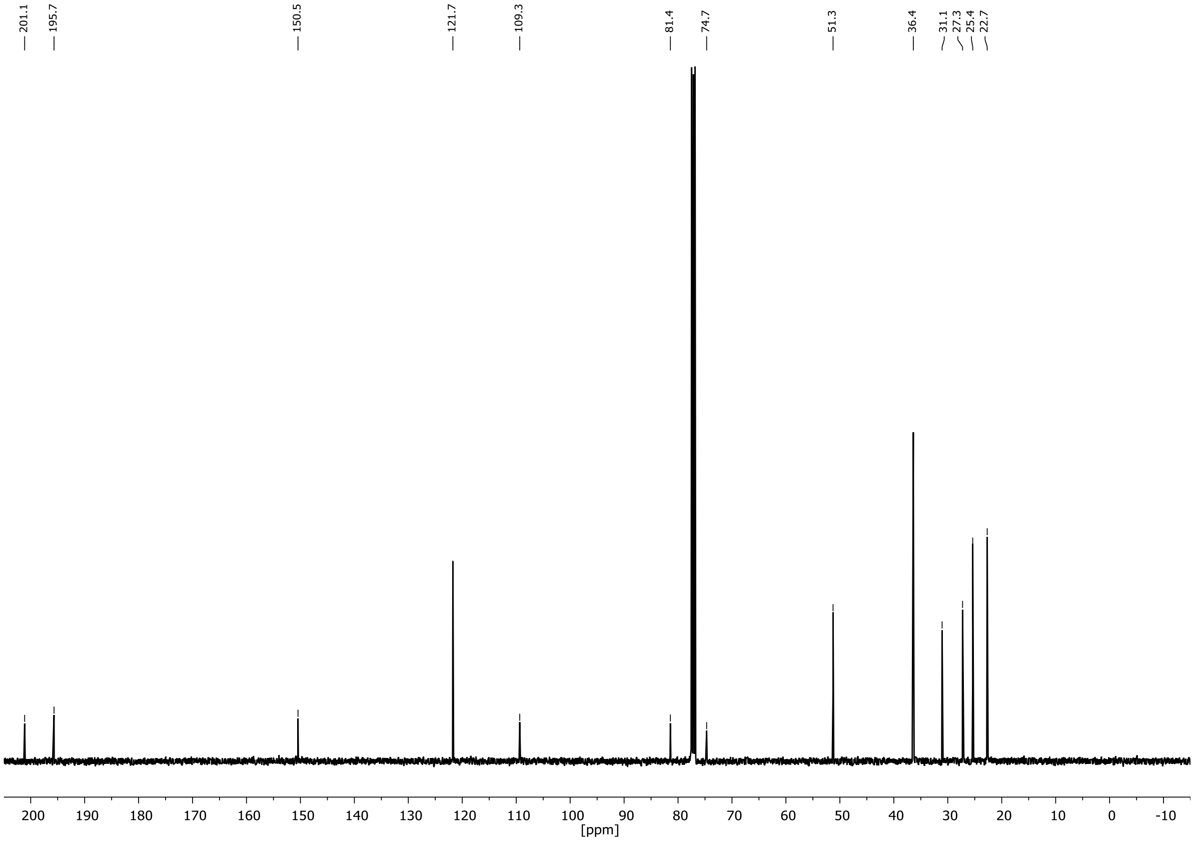
201.1
195.7
150.5
121.7
109.3
81.4
74.7
51.3
36.4
31.1
27.3
25.4
22.7
200 190 180 170 160 150 140 130 120 110 100 90 80 70 60 50 40 30 20 10 0 -10
[ppm]

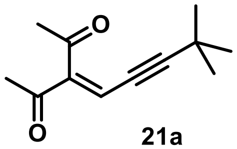

21a

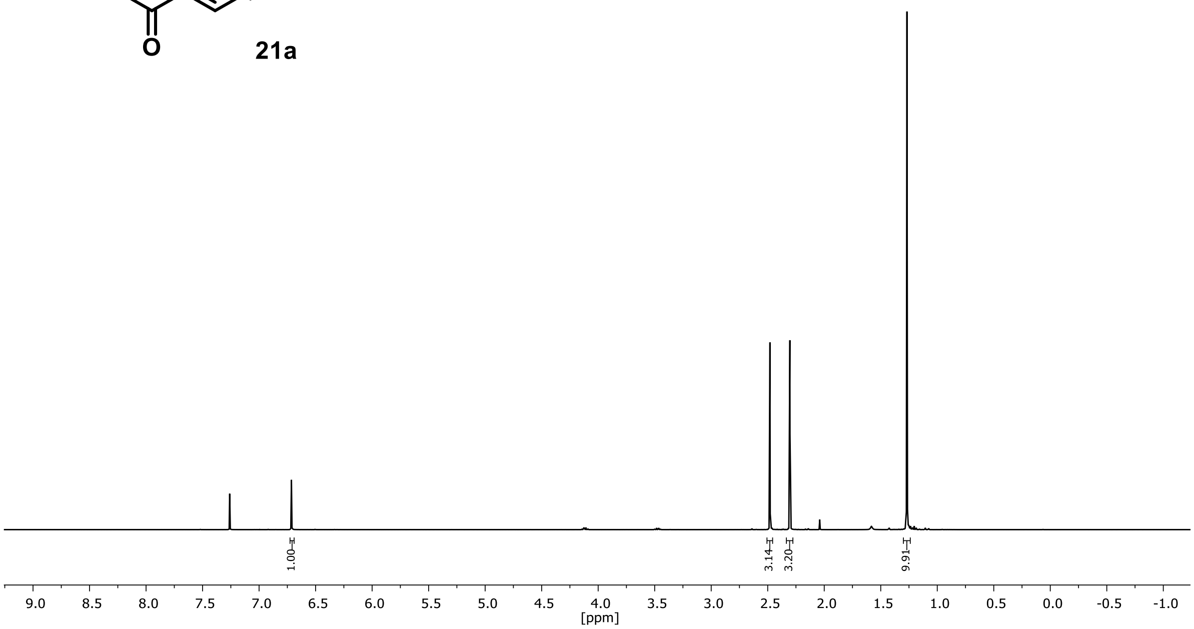
1.00
3.14
3.20
9.91
[ppm]

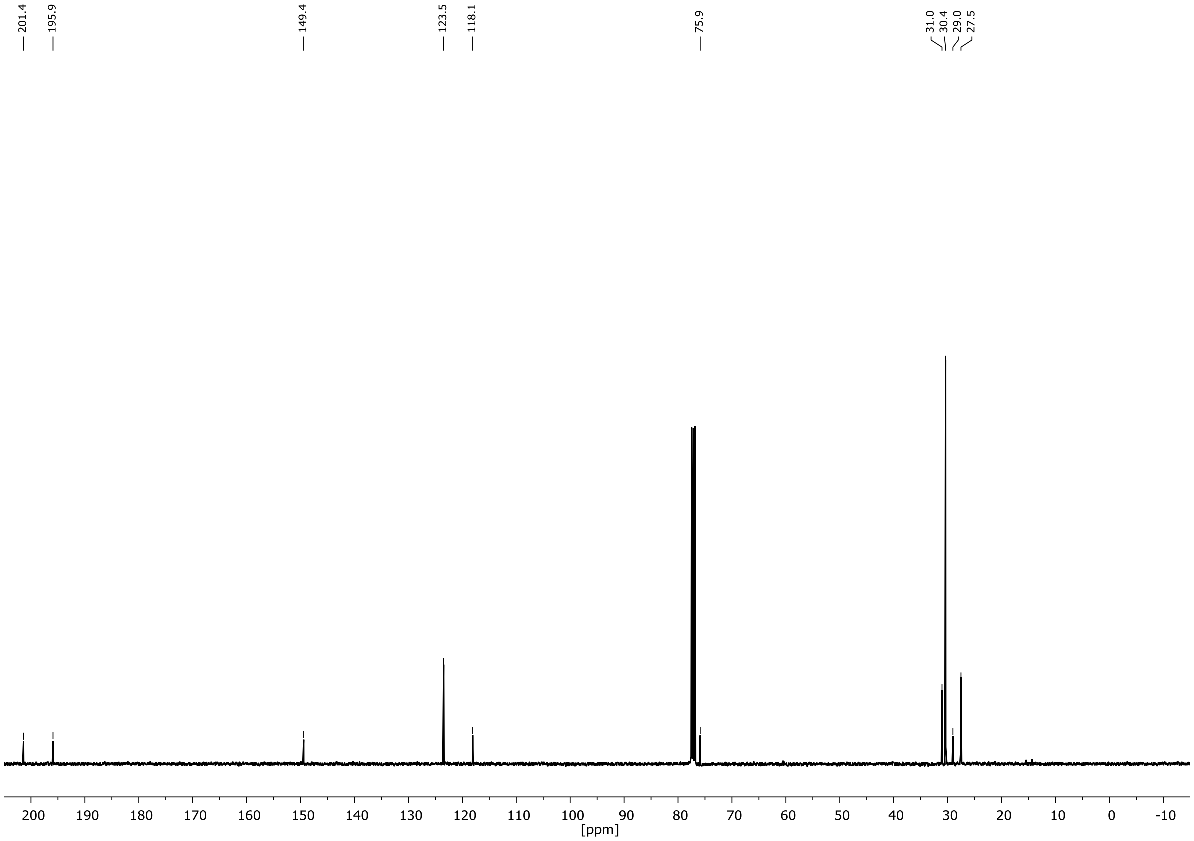
201.4
195.9
149.4
123.5
118.1
75.9
31.0
30.4
29.0
27.5
[ppm]

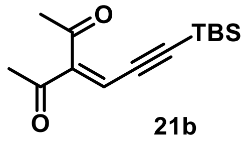
O
TBS
O

21b

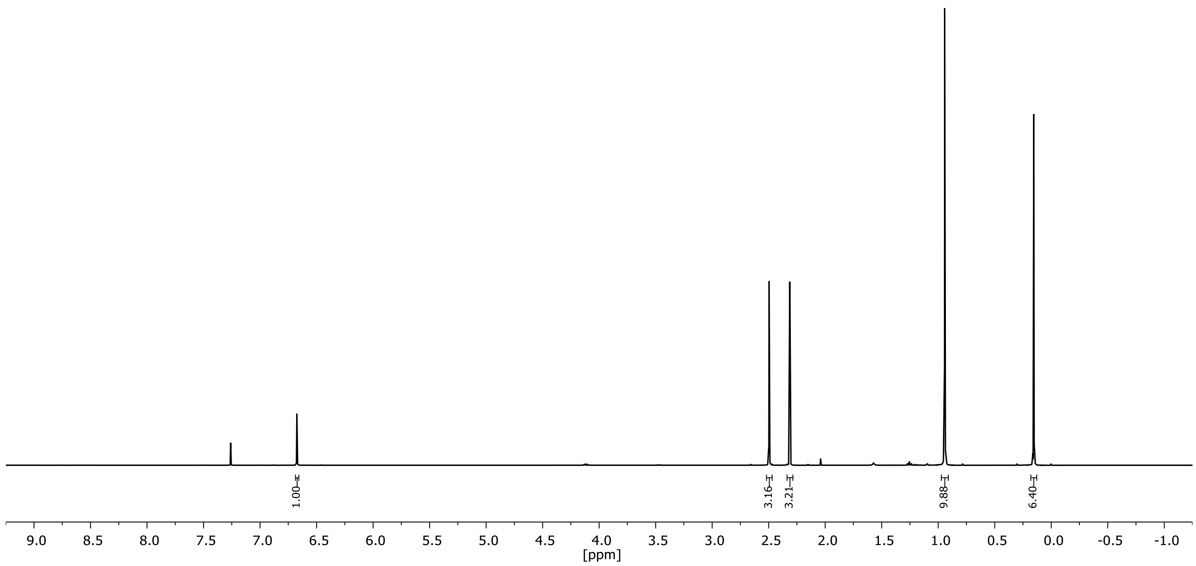
1.00
3.16
3.21
9.88
6.40
[ppm]

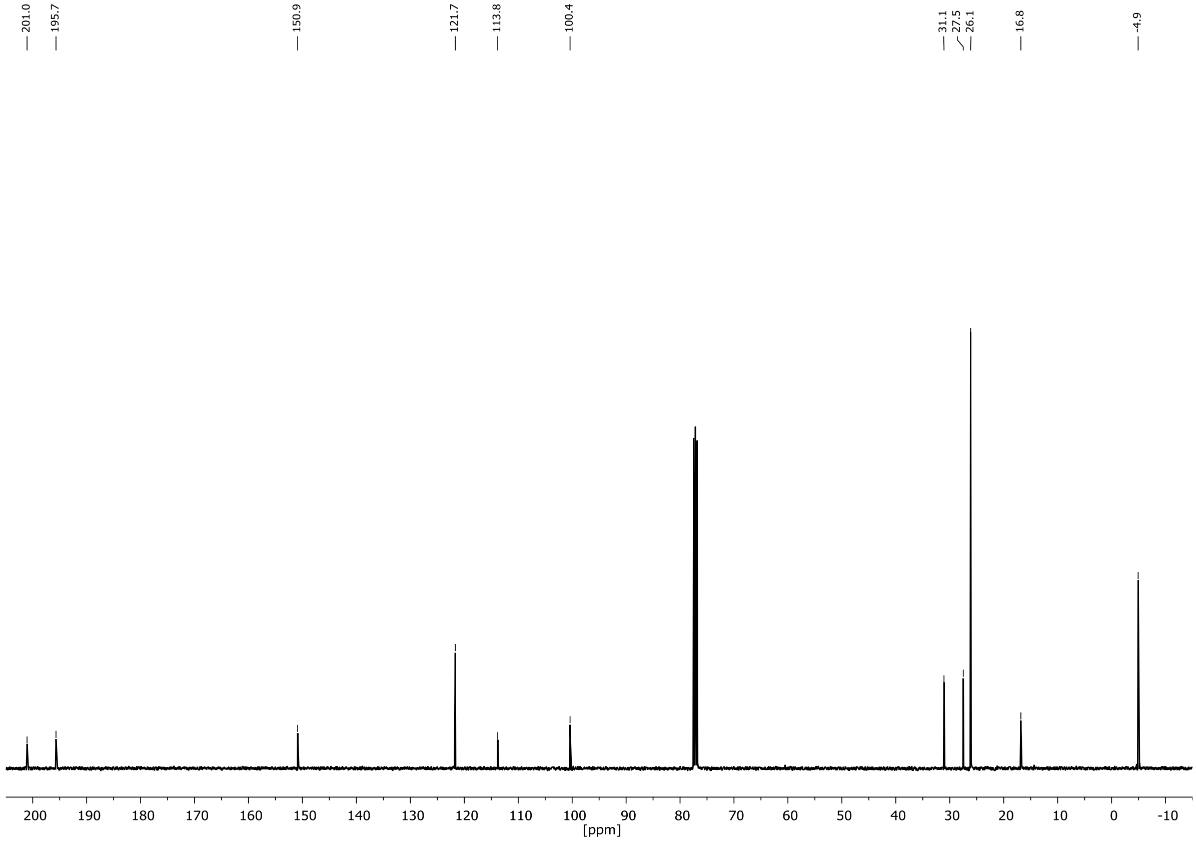
201.0
195.7
150.9
121.7
113.8
100.4
31.1
27.5
26.1
16.8
-4.9
[ppm]

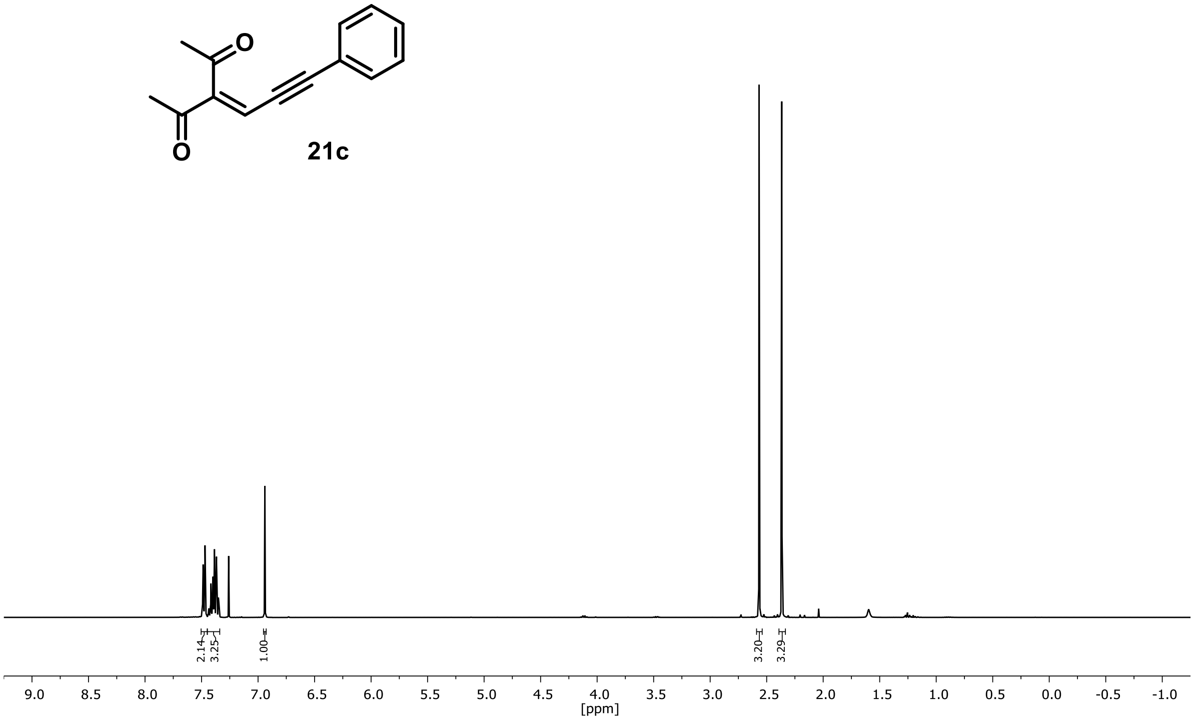

21c
2.14
3.25
1.00
3.20
3.29
9.0 8.5 8.0 7.5 7.0 6.5 6.0 5.5 5.0 4.5 4.0 3.5 3.0 2.5 2.0 1.5 1.0 0.5 0.0 -0.5 -1.0
[ppm]

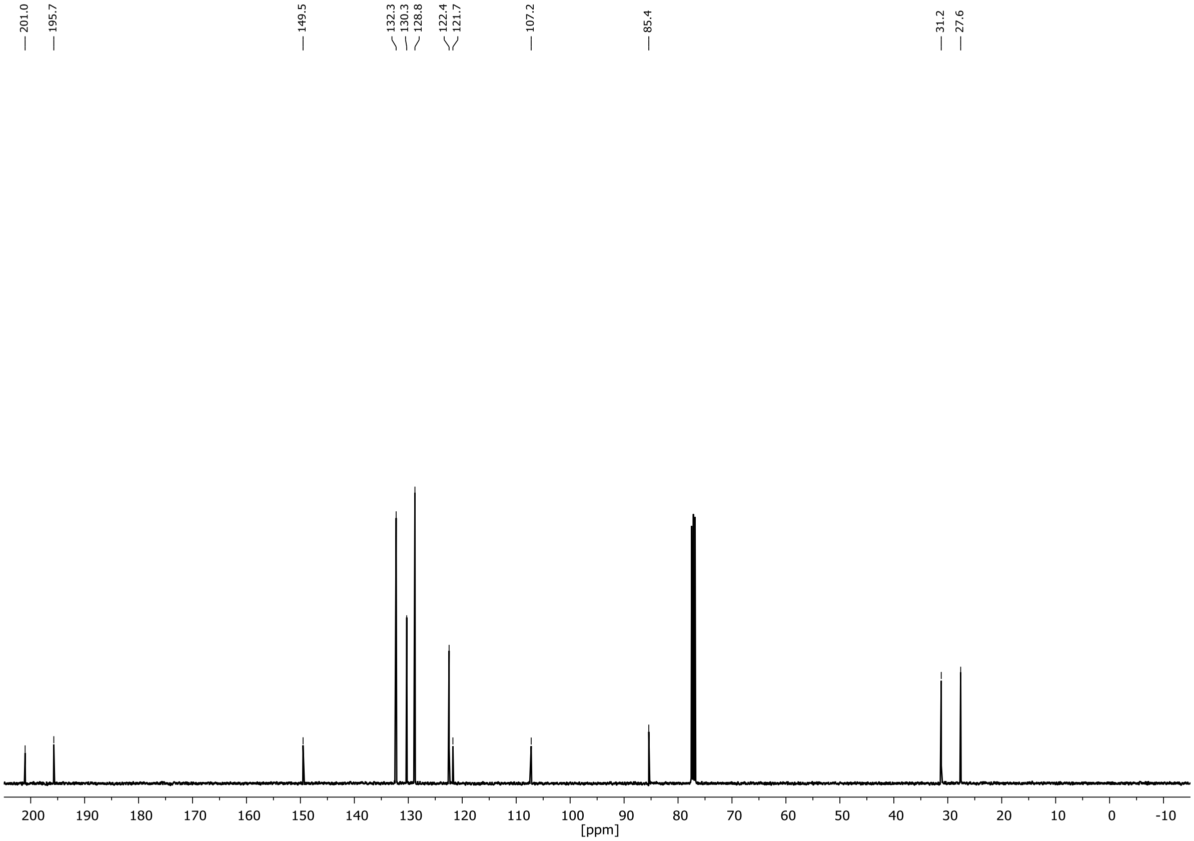
201.0
195.7
149.5
132.3
130.3
128.8
122.4
121.7
107.2
85.4
31.2
27.6
200 190 180 170 160 150 140 130 120 110 100 90 80 70 60 50 40 30 20 10 0 -10
[ppm]

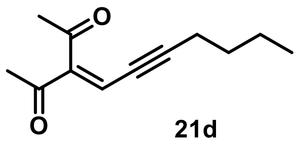

21d

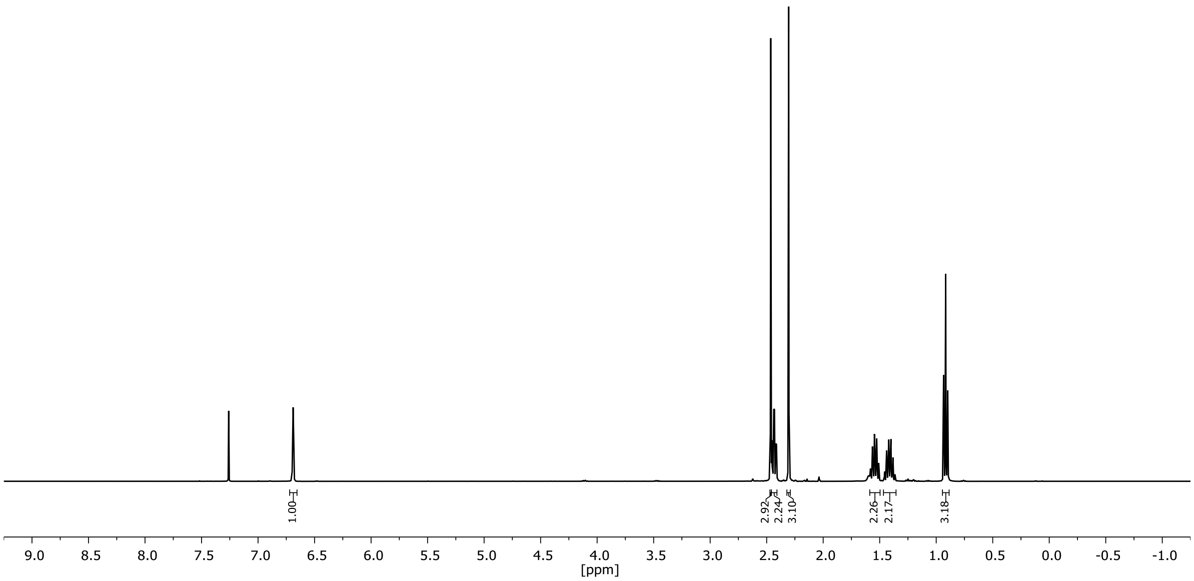

1.00
2.92
2.24
3.10
2.26
2.17
3.18
9.0 8.5 8.0 7.5 7.0 6.5 6.0 5.5 5.0 4.5 4.0 3.5 3.0 2.5 2.0 1.5 1.0 0.5 0.0 -0.5 -1.0
[ppm]

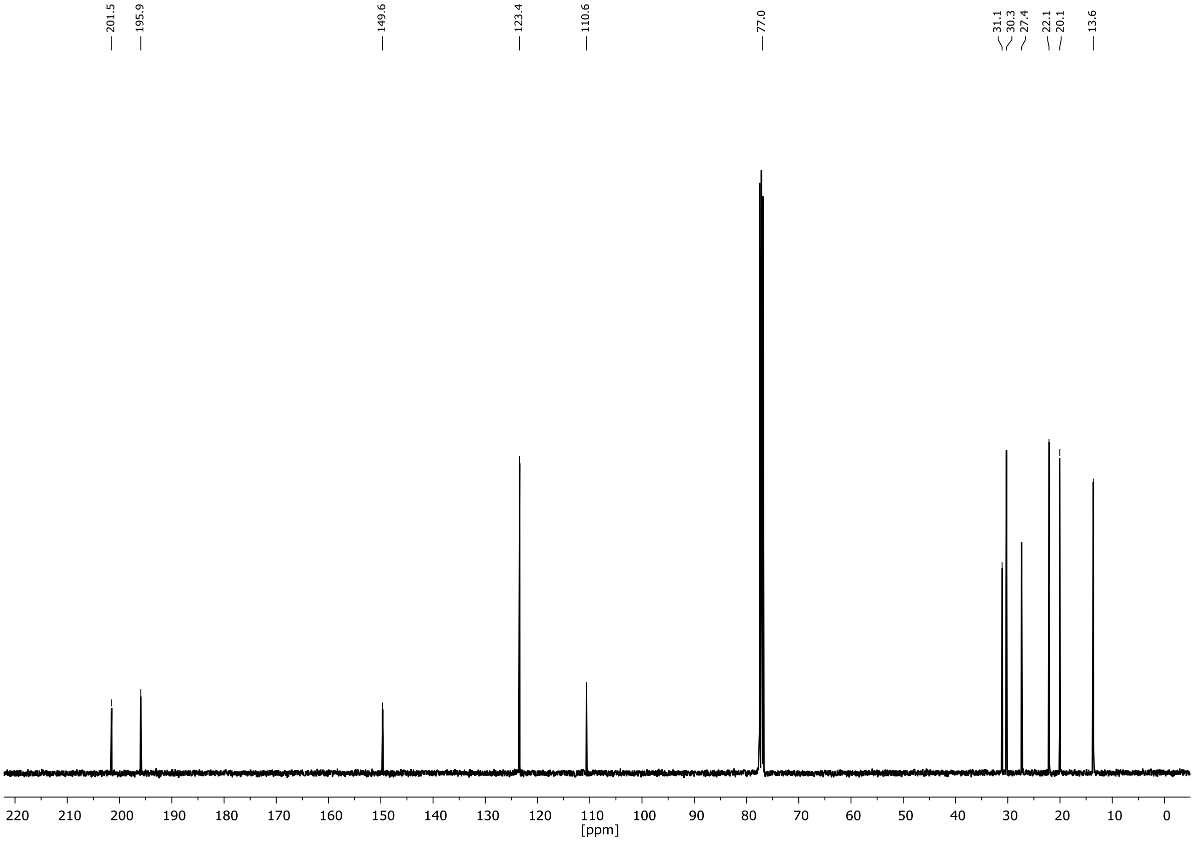

201.5
195.9
149.6
123.4
110.6
77.0
31.1
30.3
27.4
22.1
20.1
13.6
220 210 200 190 180 170 160 150 140 130 120 110 100 90 80 70 60 50 40 30 20 10 0
[ppm]

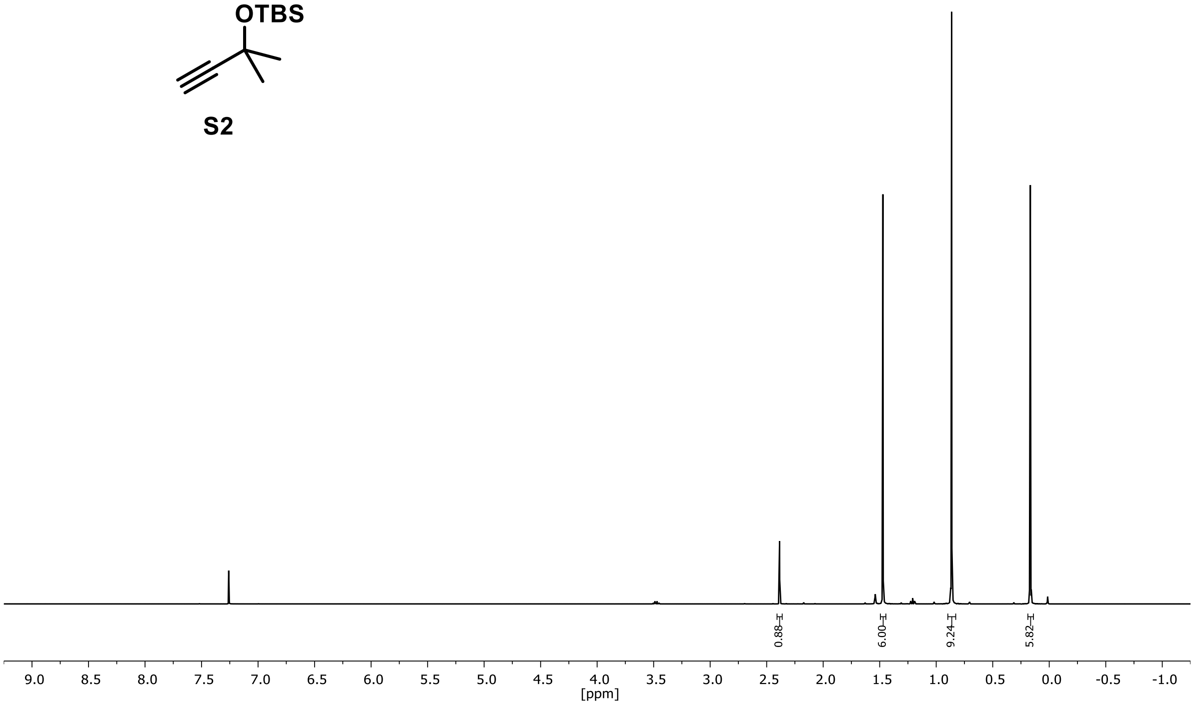
OTBS
S2
0.88
6.00
9.24
5.82
[ppm]

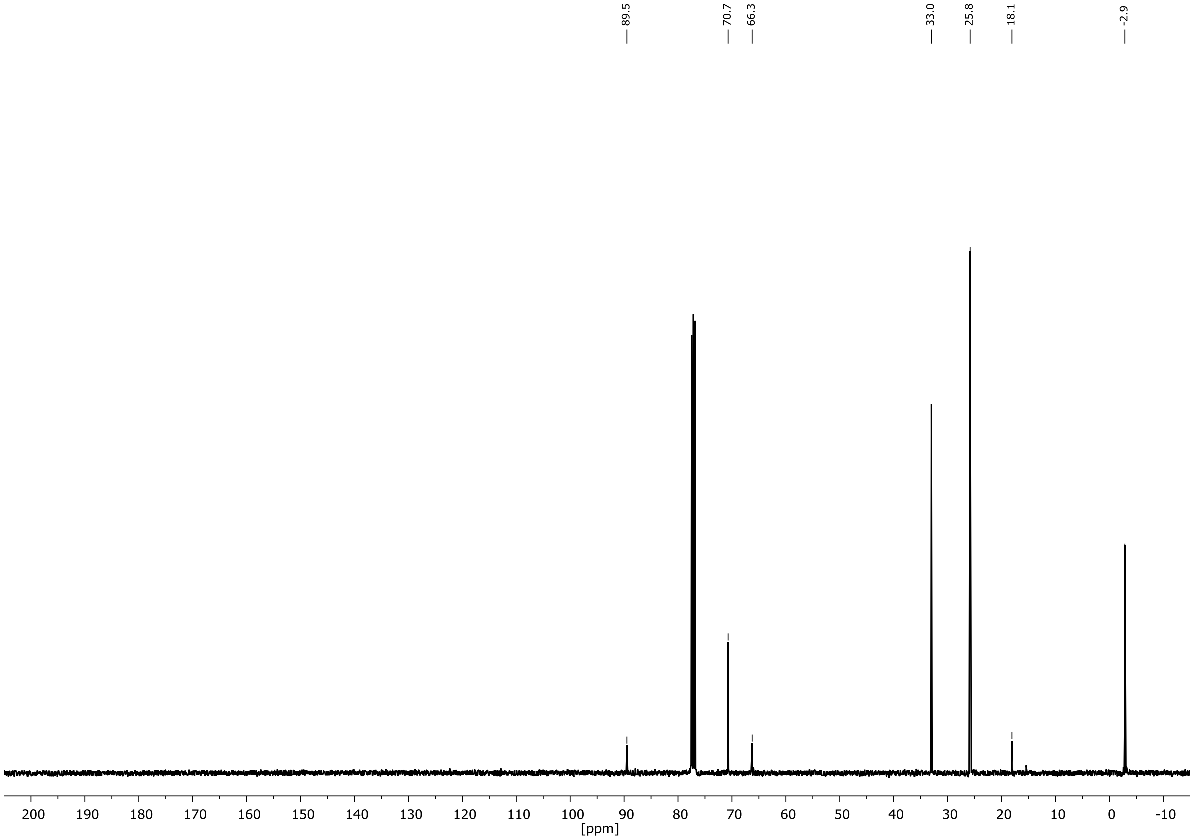
89.5
70.7
66.3
33.0
25.8
18.1
-2.9
[ppm]

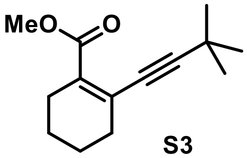
MeO
O

S3

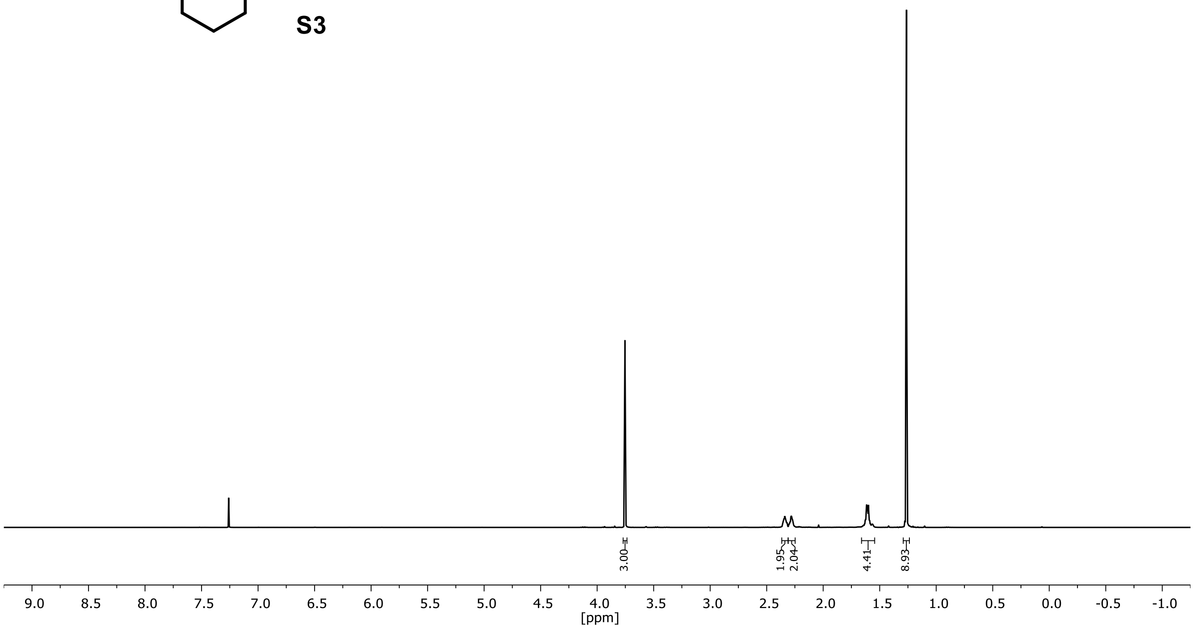
3.00
1.95
2.04
4.41
8.93
9.0 8.5 8.0 7.5 7.0 6.5 6.0 5.5 5.0 4.5 4.0 3.5 3.0 2.5 2.0 1.5 1.0 0.5 0.0 -0.5 -1.0
[ppm]

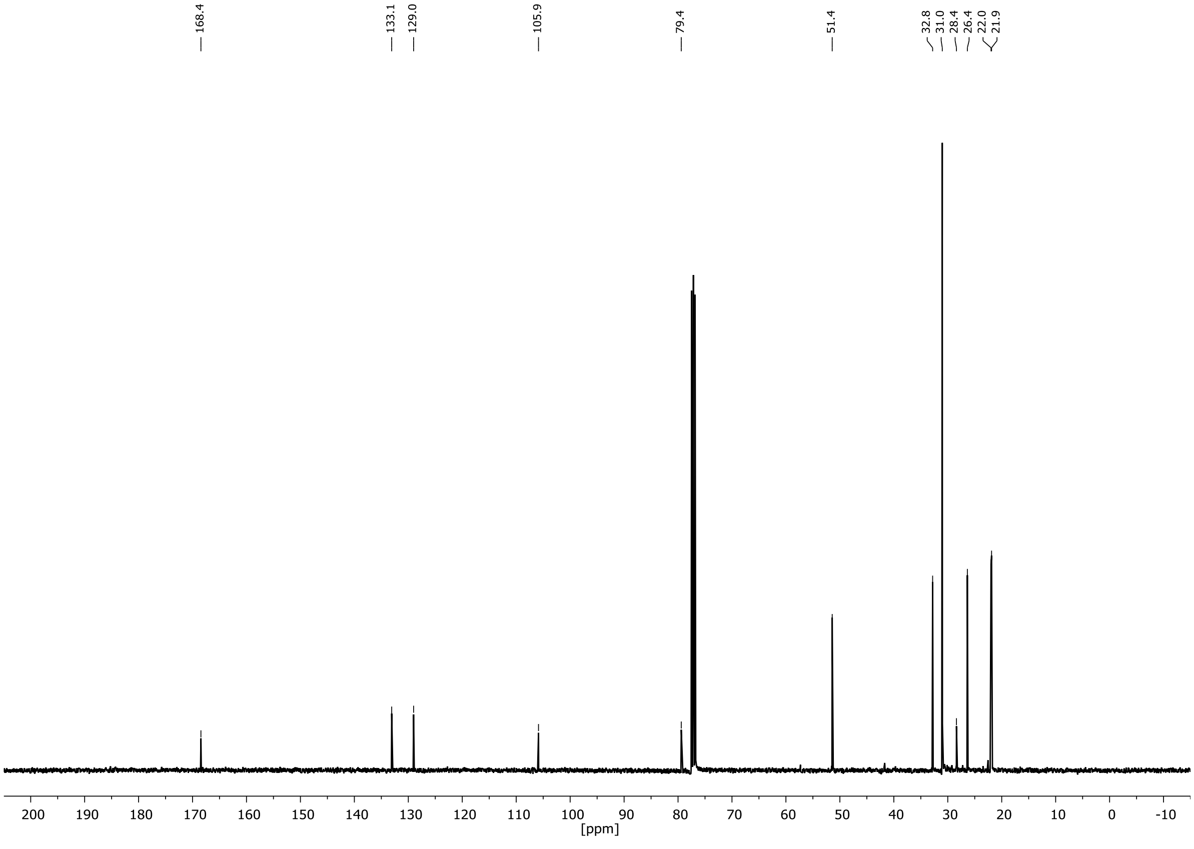
168.4
133.1
129.0
105.9
79.4
51.4
32.8
31.0
28.4
26.4
22.0
21.9
200 190 180 170 160 150 140 130 120 110 100 90 80 70 60 50 40 30 20 10 0 -10
[ppm]

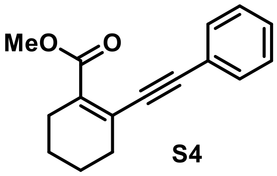

S4

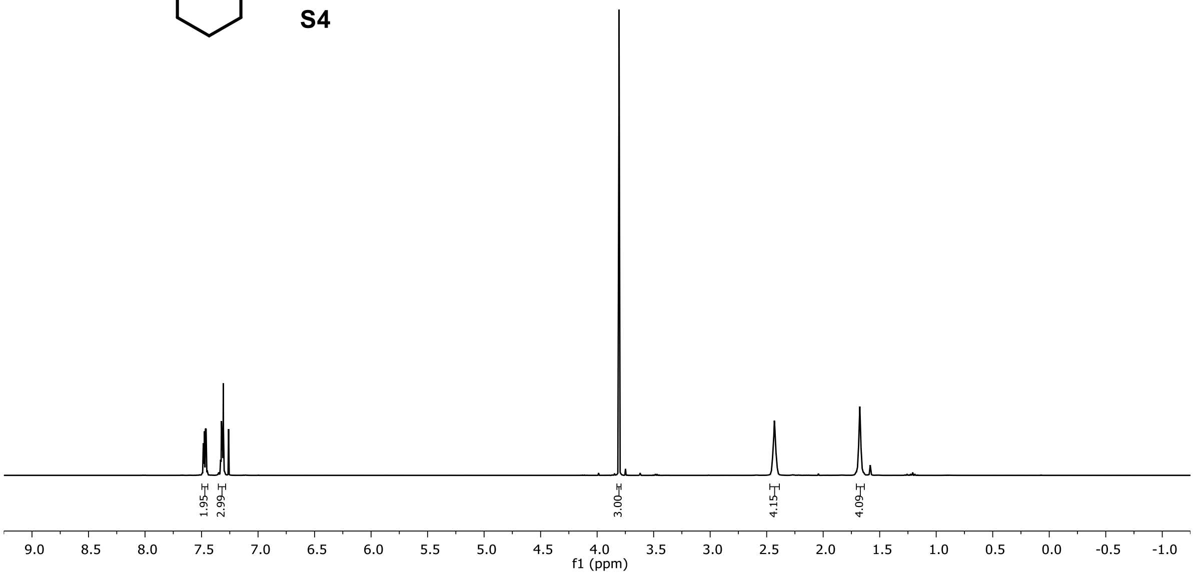
1.95
2.99
3.00
4.15
4.09
9.0 8.5 8.0 7.5 7.0 6.5 6.0 5.5 5.0 4.5 4.0 3.5 3.0 2.5 2.0 1.5 1.0 0.5 0.0 -0.5 -1.0
f1 (ppm)

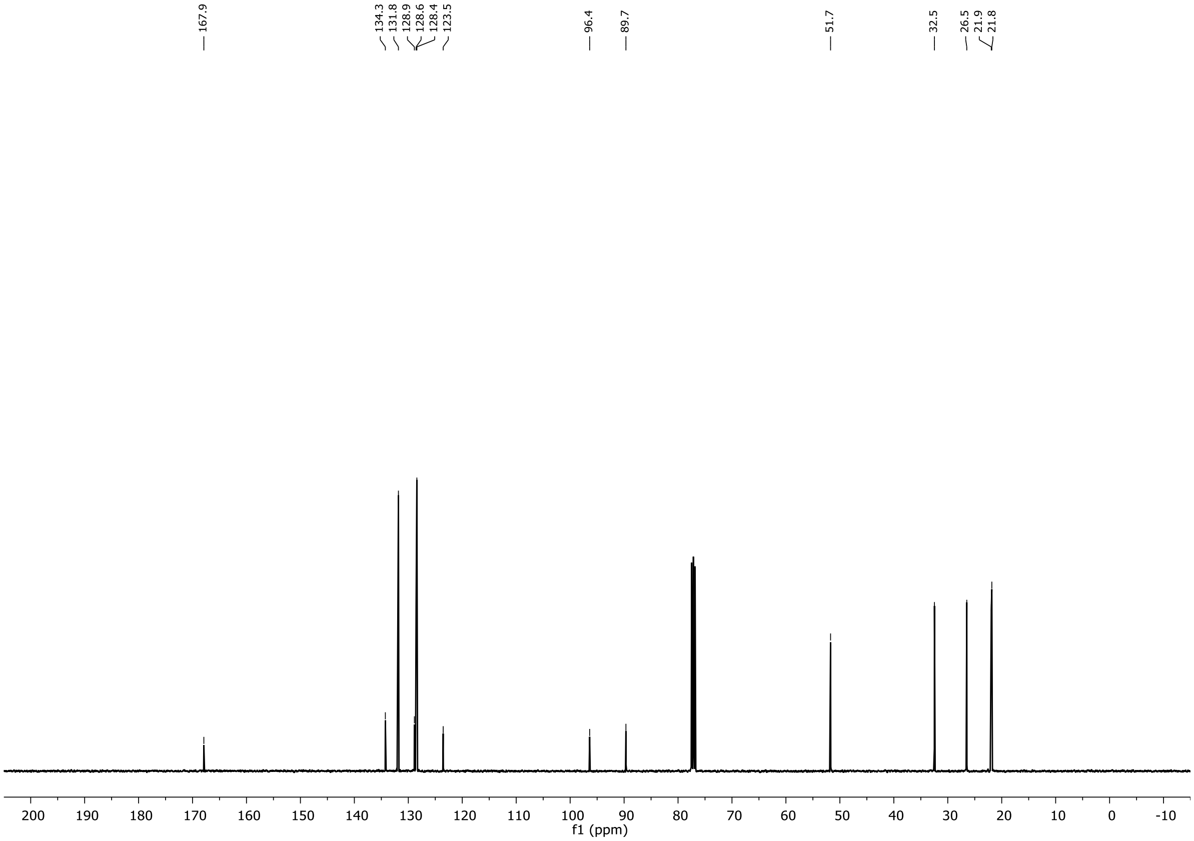
167.9
134.3
131.8
128.9
128.6
128.4
123.5
96.4
89.7
51.7
32.5
26.5
21.9
21.8
200 190 180 170 160 150 140 130 120 110 100 90 80 70 60 50 40 30 20 10 0 -10
f1 (ppm)

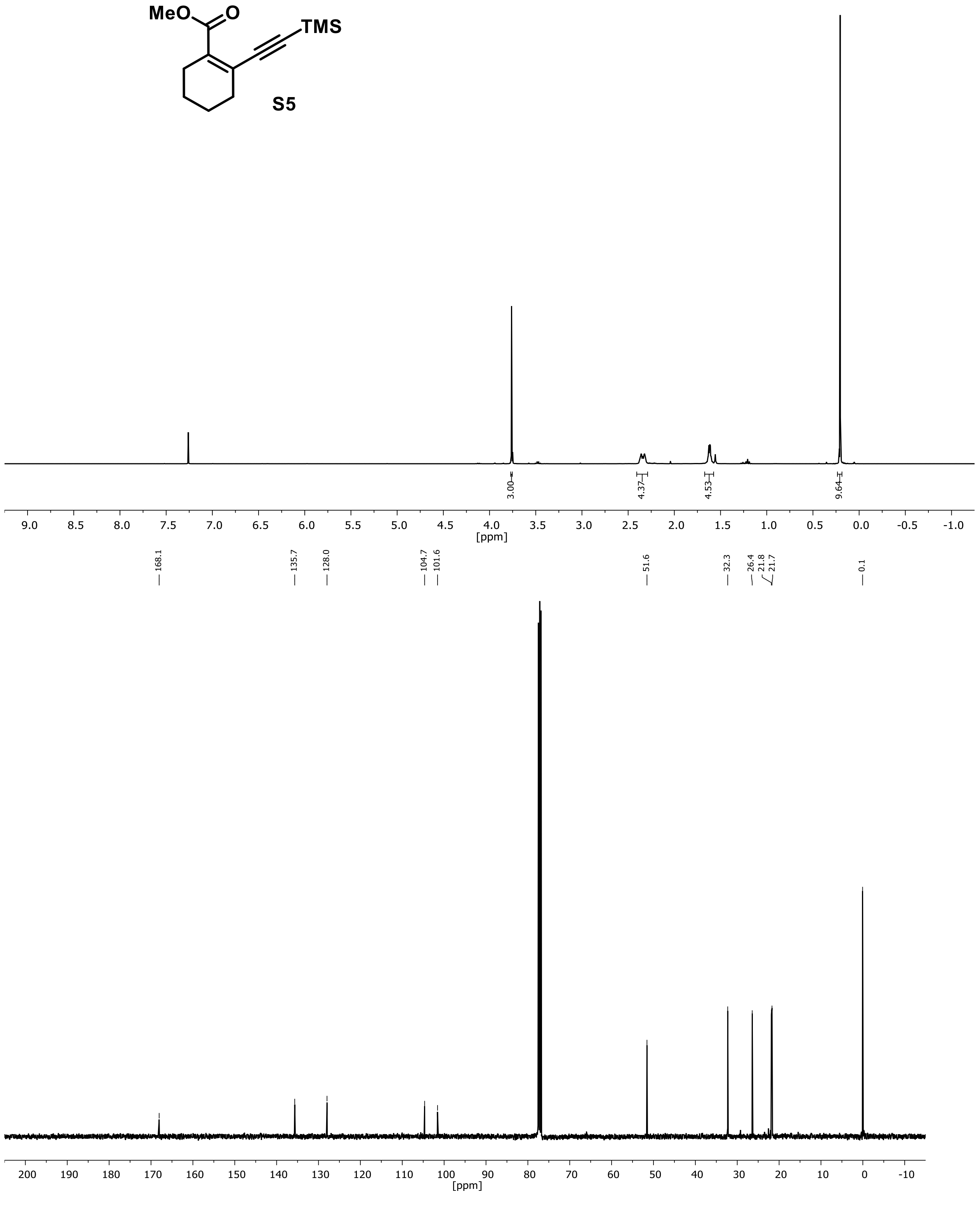
MeO
O
TMS
S5
3.00
4.37
4.53
9.64
9.0 8.5 8.0 7.5 7.0 6.5 6.0 5.5 5.0 4.5 4.0 3.5 3.0 2.5 2.0 1.5 1.0 0.5 0.0 -0.5 -1.0
[ppm]
168.1
135.7
128.0
104.7
101.6
51.6
32.3
26.4
21.8
21.7
0.1
200 190 180 170 160 150 140 130 120 110 100 90 80 70 60 50 40 30 20 10 0 -10
[ppm]

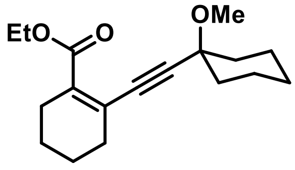
EtO
O
OMe

S7

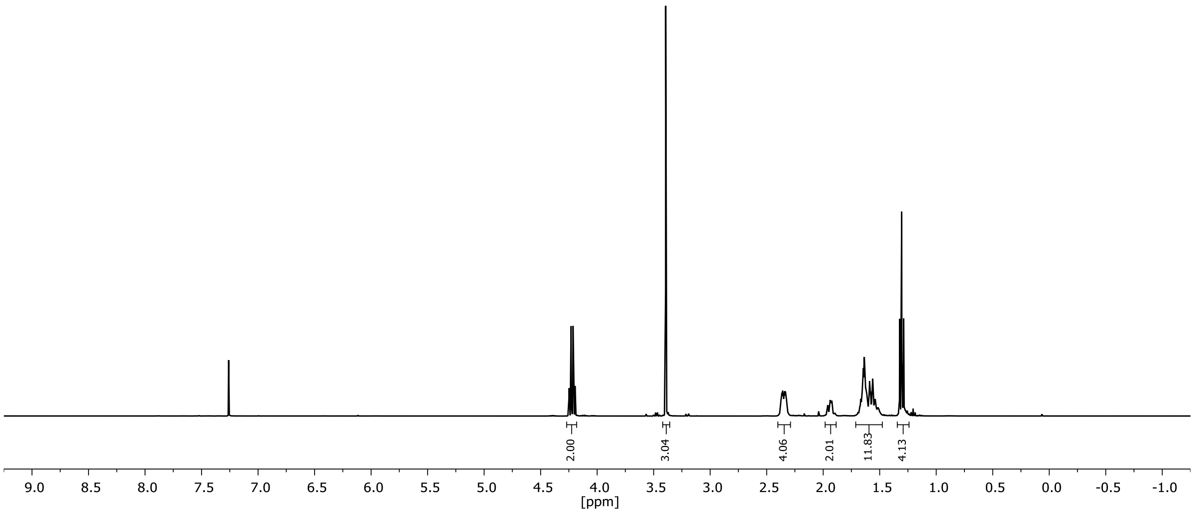
2.00
3.04
4.06
2.01
11.83
4.13
9.0 8.5 8.0 7.5 7.0 6.5 6.0 5.5 5.0 4.5 4.0 3.5 3.0 2.5 2.0 1.5 1.0 0.5 0.0 -0.5 -1.0
[ppm]

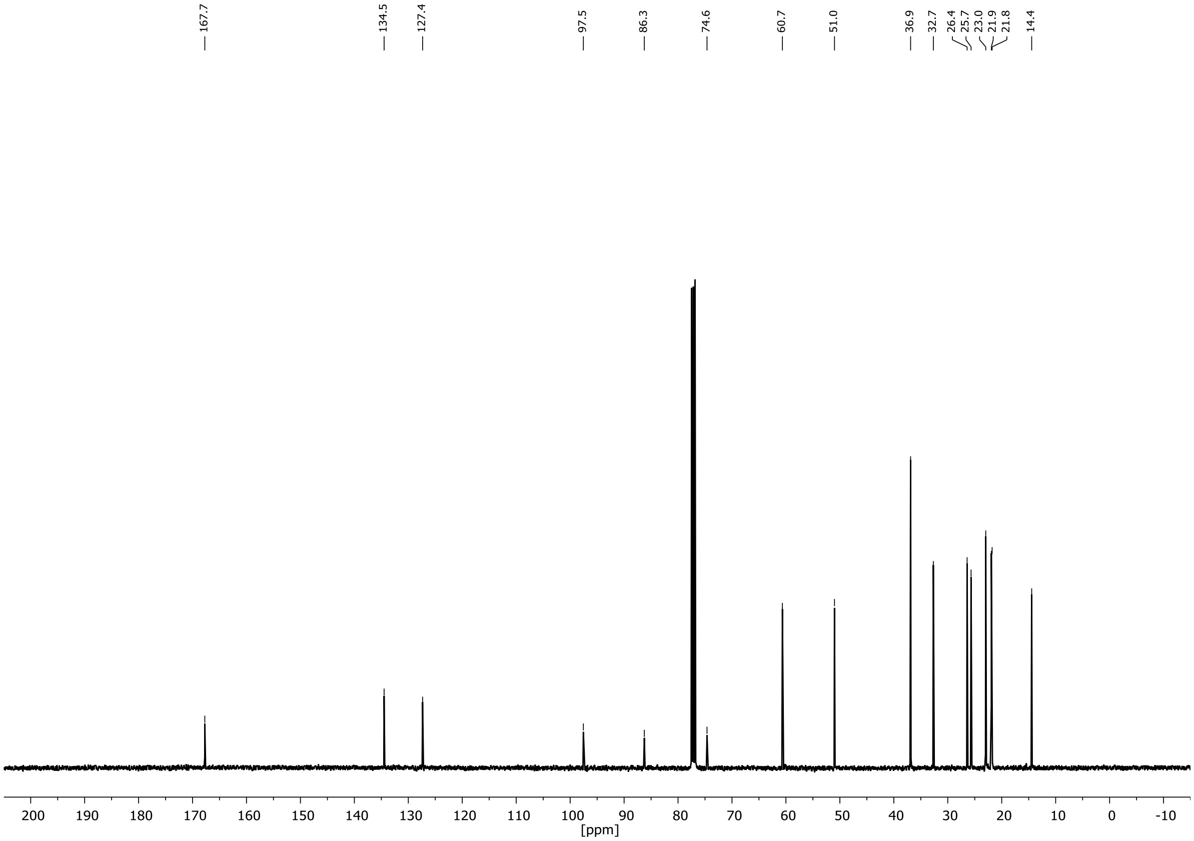
167.7
134.5
127.4
97.5
86.3
74.6
60.7
51.0
36.9
32.7
26.4
25.7
23.0
21.9
21.8
14.4
200 190 180 170 160 150 140 130 120 110 100 90 80 70 60 50 40 30 20 10 0 -10
[ppm]

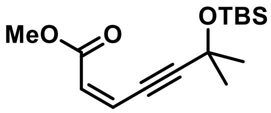
MeO
O
OTBS

S9

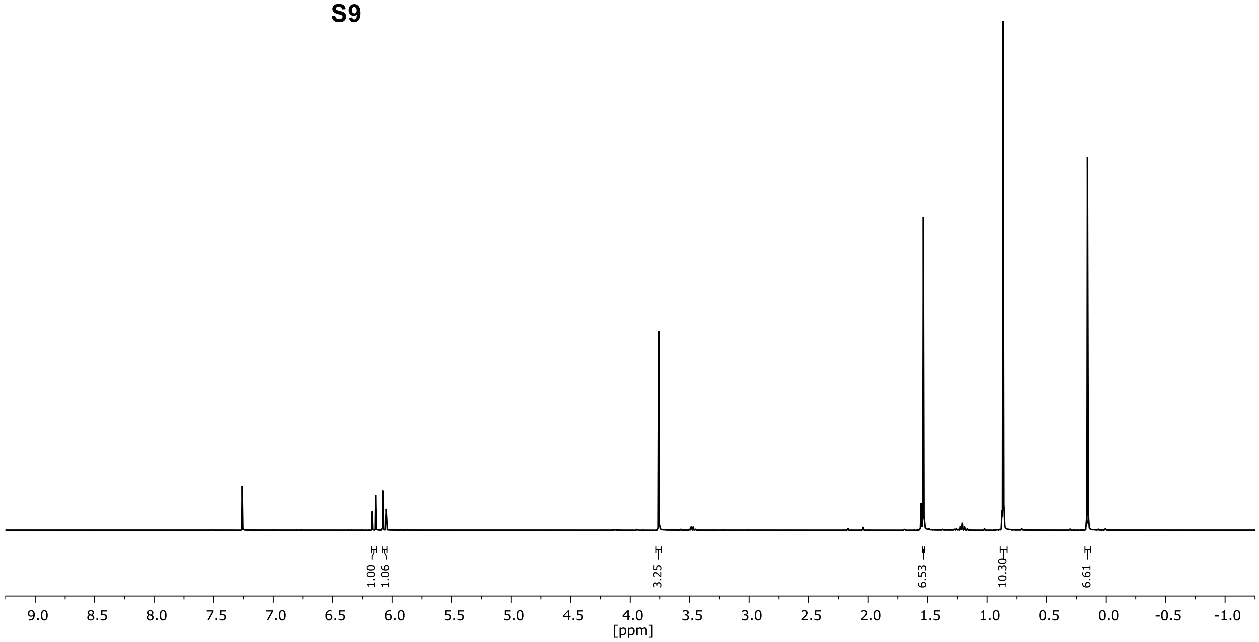
1.00
1.06
3.25
6.53
10.30
6.61
9.0 8.5 8.0 7.5 7.0 6.5 6.0 5.5 5.0 4.5 4.0 3.5 3.0 2.5 2.0 1.5 1.0 0.5 0.0 -0.5 -1.0
[ppm]

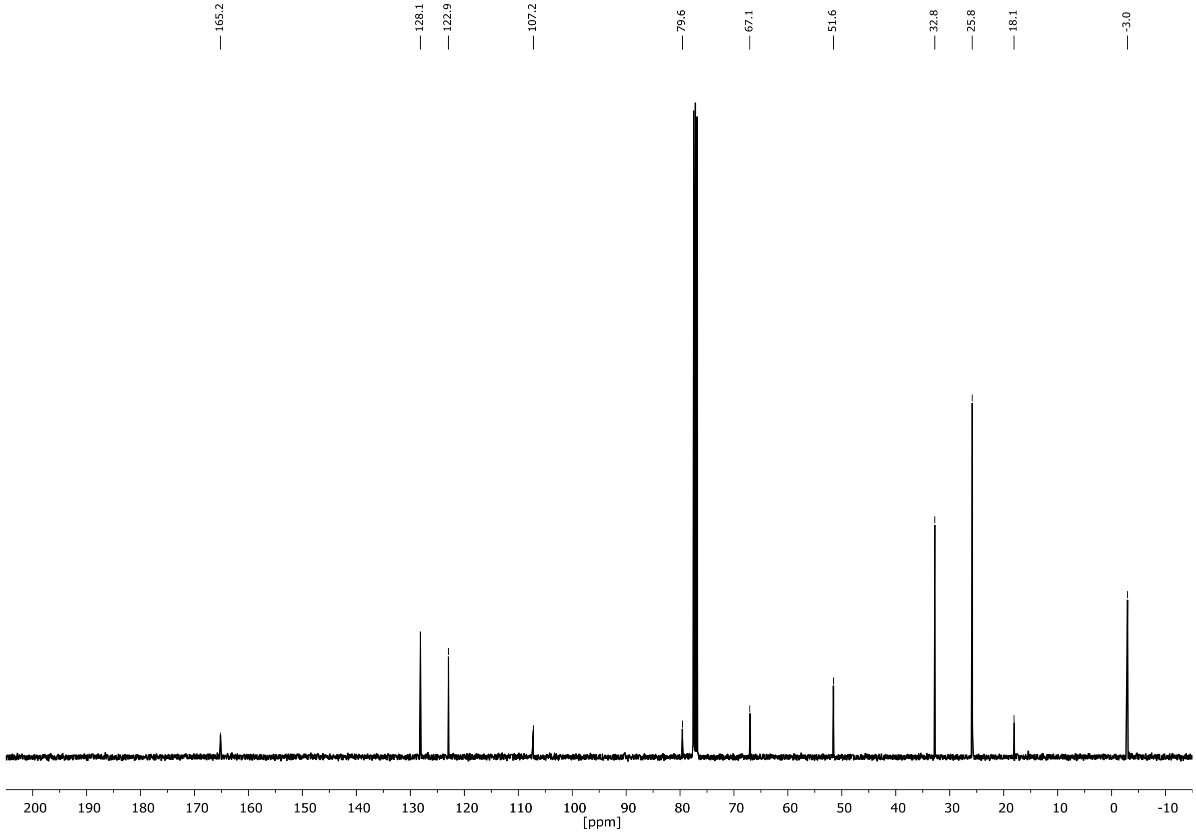
165.2
128.1
122.9
107.2
79.6
67.1
51.6
32.8
25.8
18.1
-3.0
200 190 180 170 160 150 140 130 120 110 100 90 80 70 60 50 40 30 20 10 0 -10
[ppm]

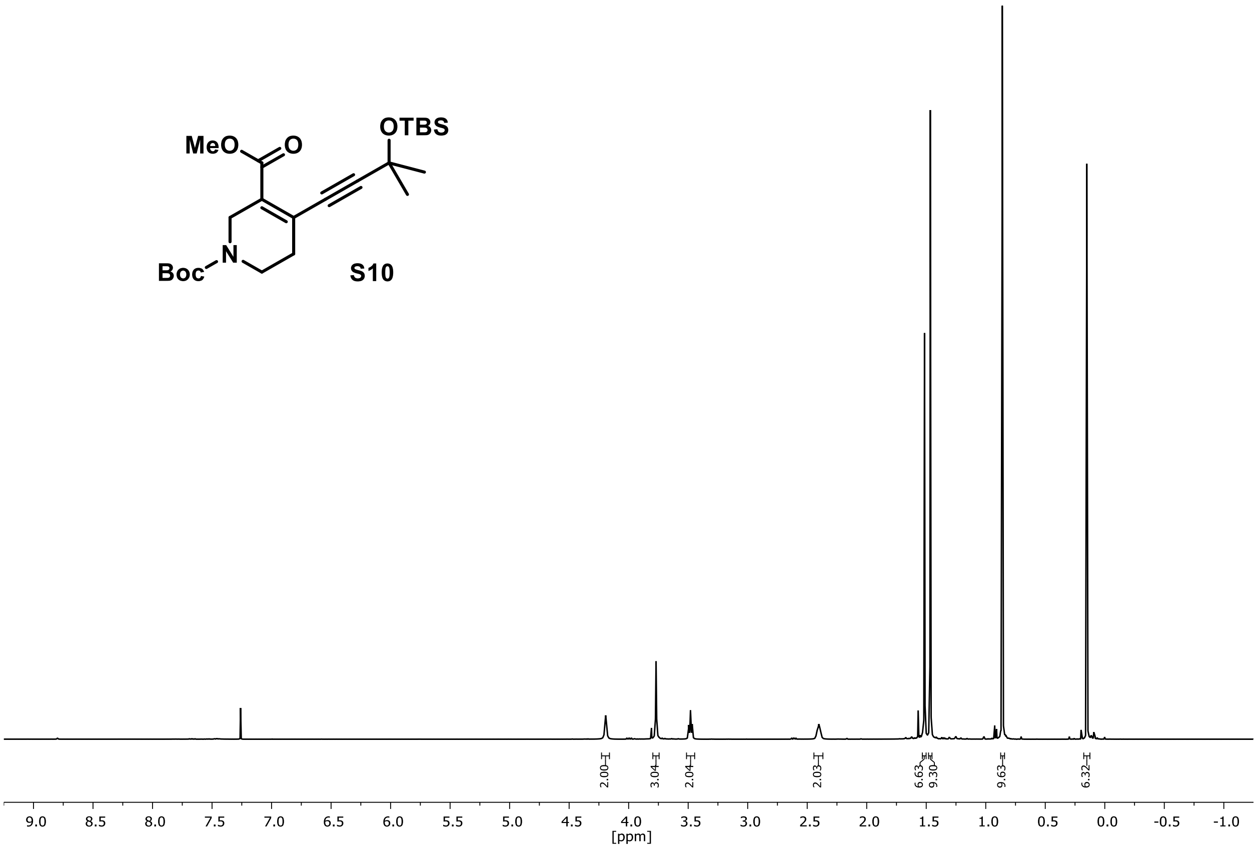
MeO
O
OTBS
N
Boc
S10
2.00
3.04
2.04
2.03
6.63
9.30
9.63
6.32
9.0 8.5 8.0 7.5 7.0 6.5 6.0 5.5 5.0 4.5 4.0 3.5 3.0 2.5 2.0 1.5 1.0 0.5 0.0 -0.5 -1.0
[ppm]

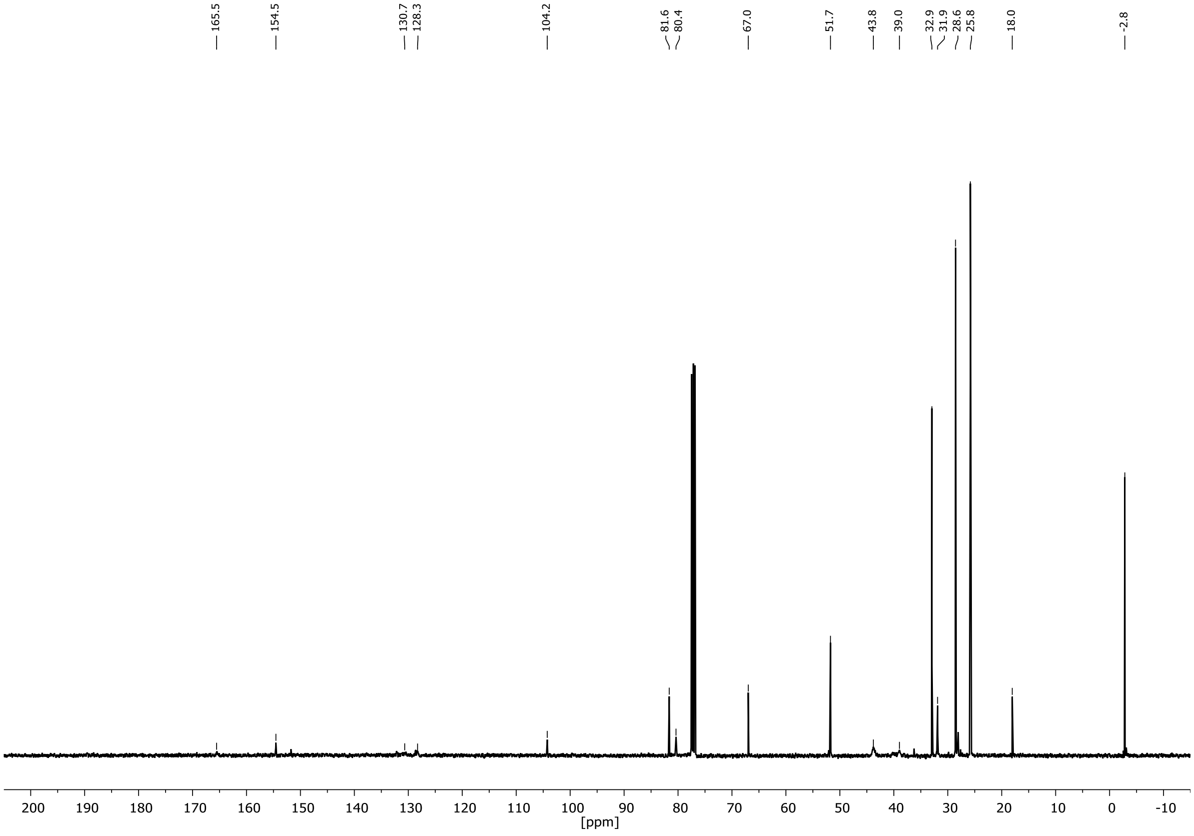
165.5
154.5
130.7
128.3
104.2
81.6
80.4
67.0
51.7
43.8
39.0
32.9
31.9
28.6
25.8
18.0
-2.8
200 190 180 170 160 150 140 130 120 110 100 90 80 70 60 50 40 30 20 10 0 -10
[ppm]

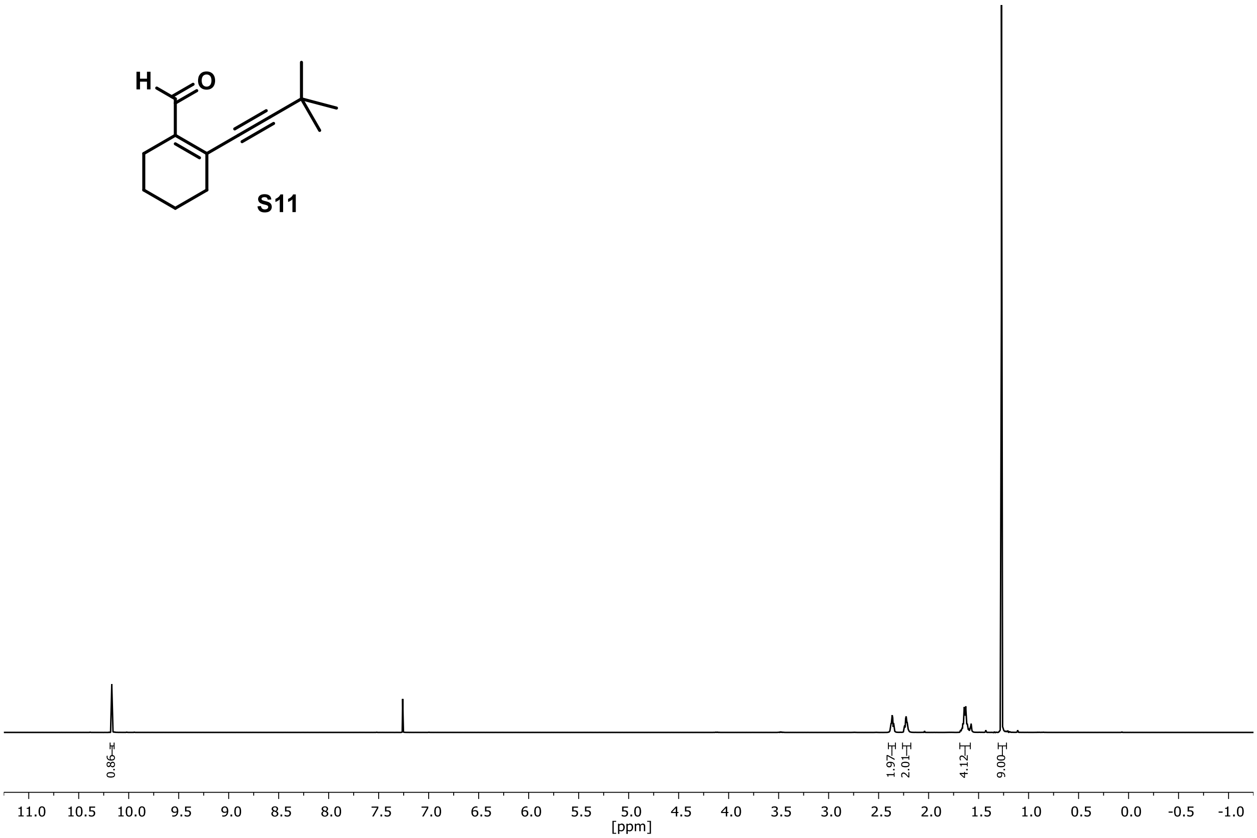
H
O
S11
0.86
1.97
2.01
4.12
9.00
11.0 10.5 10.0 9.5 9.0 8.5 8.0 7.5 7.0 6.5 6.0 5.5 5.0 4.5 4.0 3.5 3.0 2.5 2.0 1.5 1.0 0.5 0.0 -0.5 -1.0
[ppm]

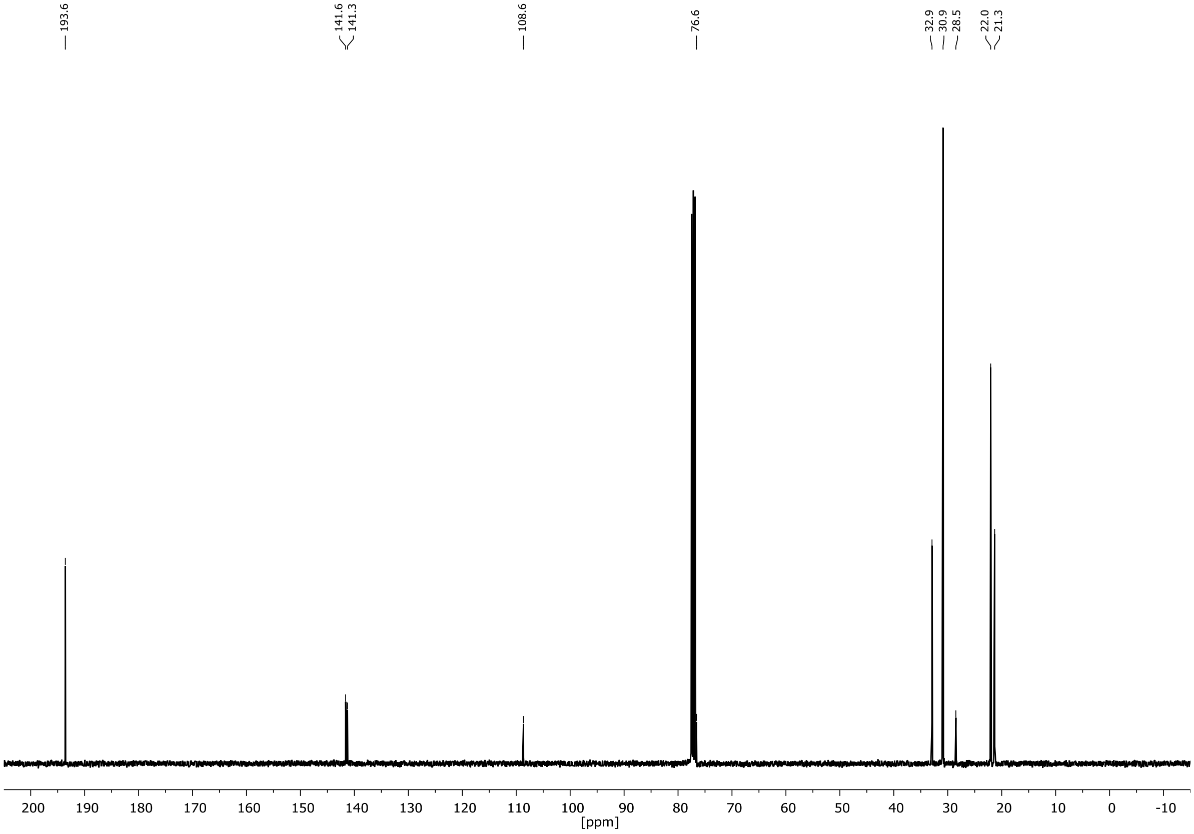
193.6
141.6
141.3
108.6
76.6
32.9
30.9
28.5
22.0
21.3
200 190 180 170 160 150 140 130 120 110 100 90 80 70 60 50 40 30 20 10 0 -10
[ppm]

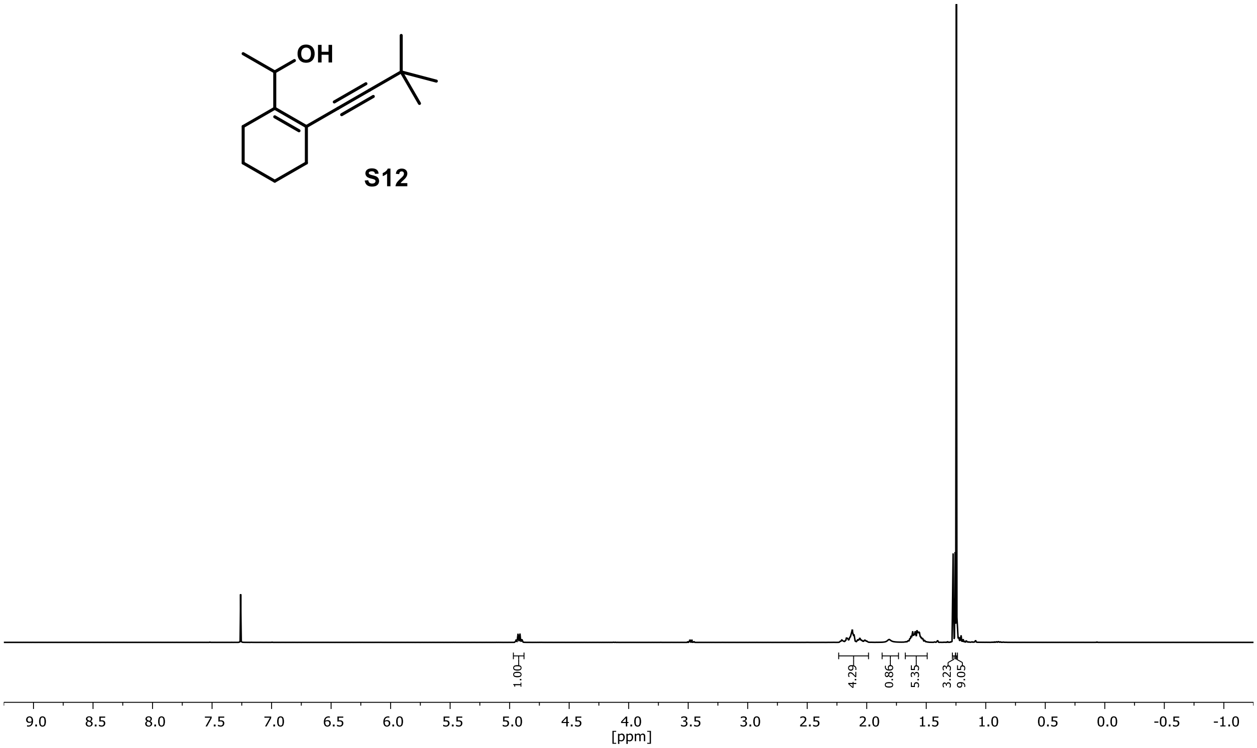
OH
S12
1.00
4.29
0.86
5.35
3.23
9.05
9.0 8.5 8.0 7.5 7.0 6.5 6.0 5.5 5.0 4.5 4.0 3.5 3.0 2.5 2.0 1.5 1.0 0.5 0.0 -0.5 -1.0
[ppm]

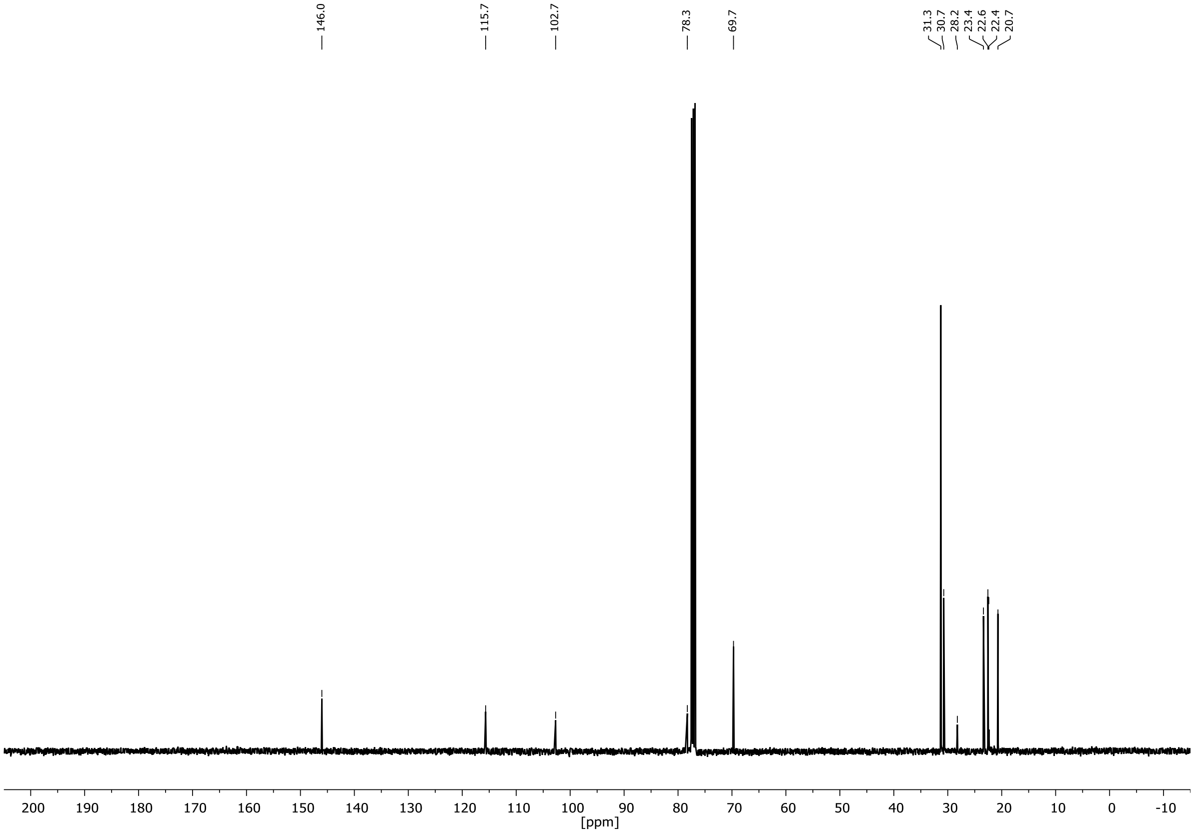
146.0
115.7
102.7
78.3
69.7
31.3
30.7
28.2
23.4
22.6
22.4
20.7
200 190 180 170 160 150 140 130 120 110 100 90 80 70 60 50 40 30 20 10 0 -10
[ppm]

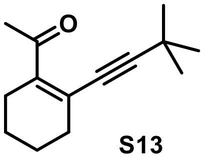
O

S13

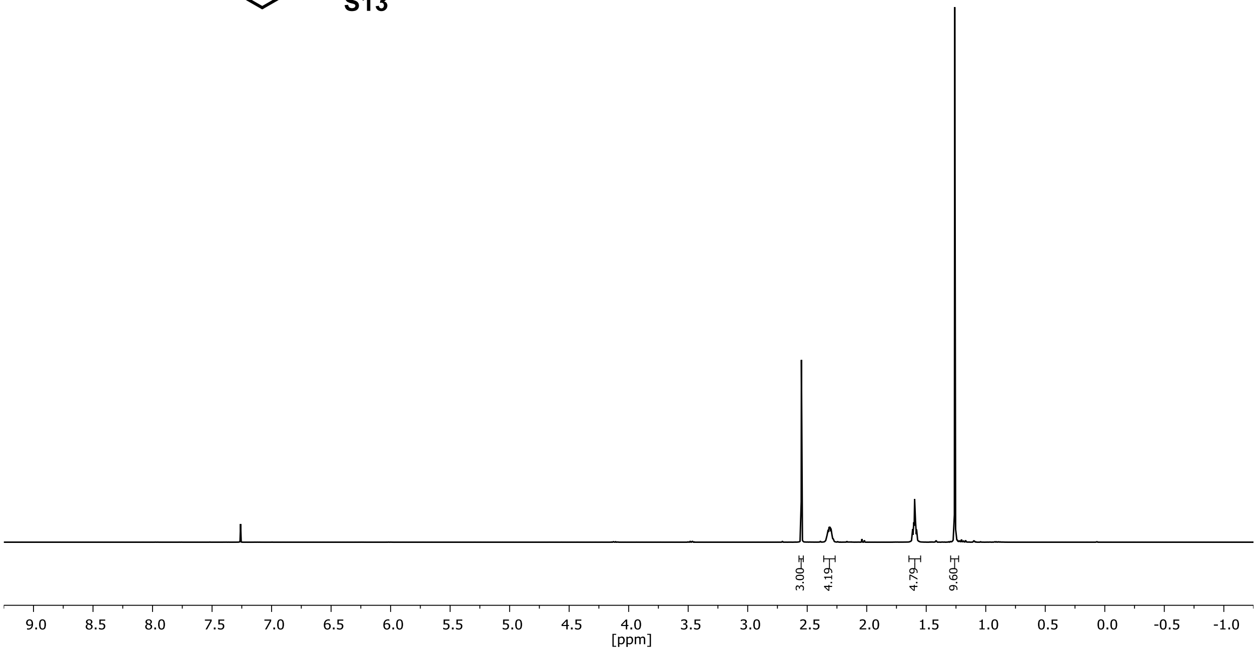
3.00
4.19
4.79
9.60
9.0 8.5 8.0 7.5 7.0 6.5 6.0 5.5 5.0 4.5 4.0 3.5 3.0 2.5 2.0 1.5 1.0 0.5 0.0 -0.5 -1.0
[ppm]

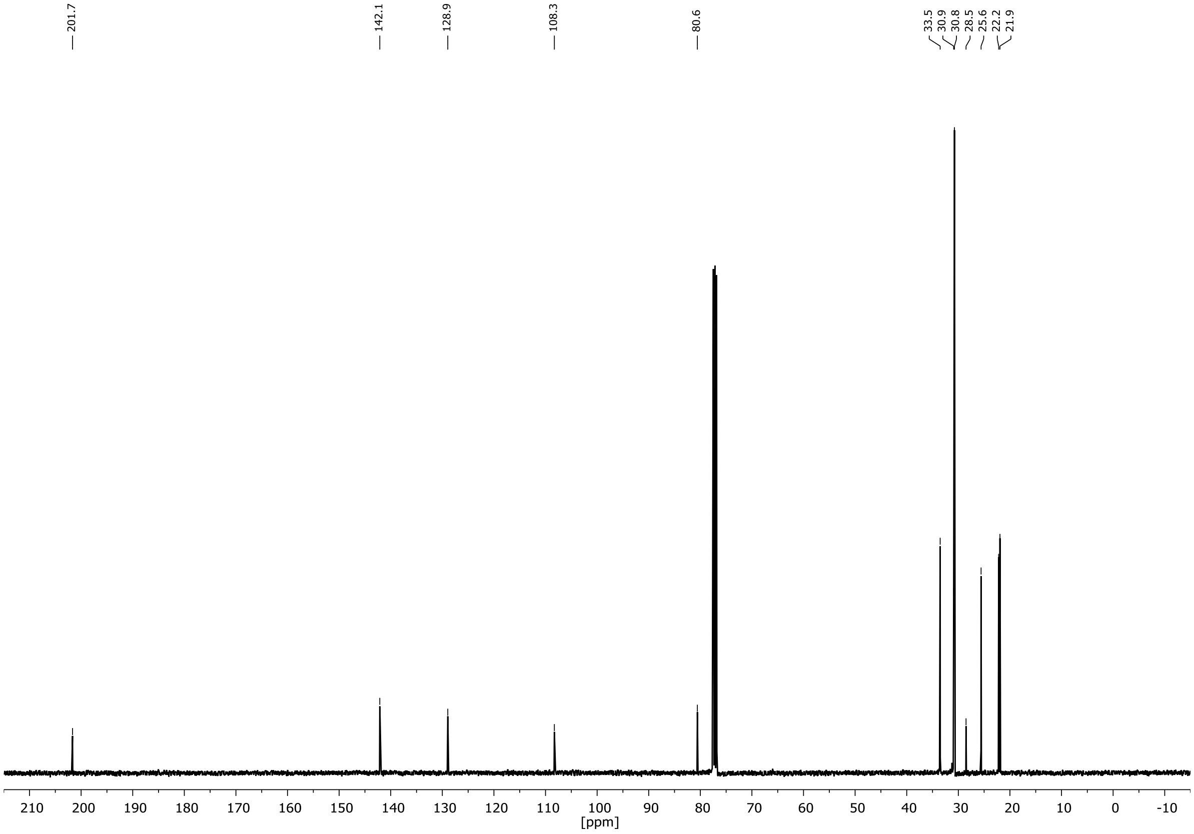
201.7
142.1
128.9
108.3
80.6
33.5
30.9
30.8
28.5
25.6
22.2
21.9
210 200 190 180 170 160 150 140 130 120 110 100 90 80 70 60 50 40 30 20 10 0 -10
[ppm]

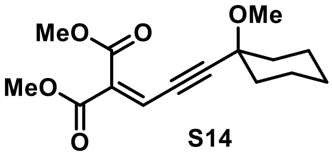
MeO
O
MeO
O
OMe

S14

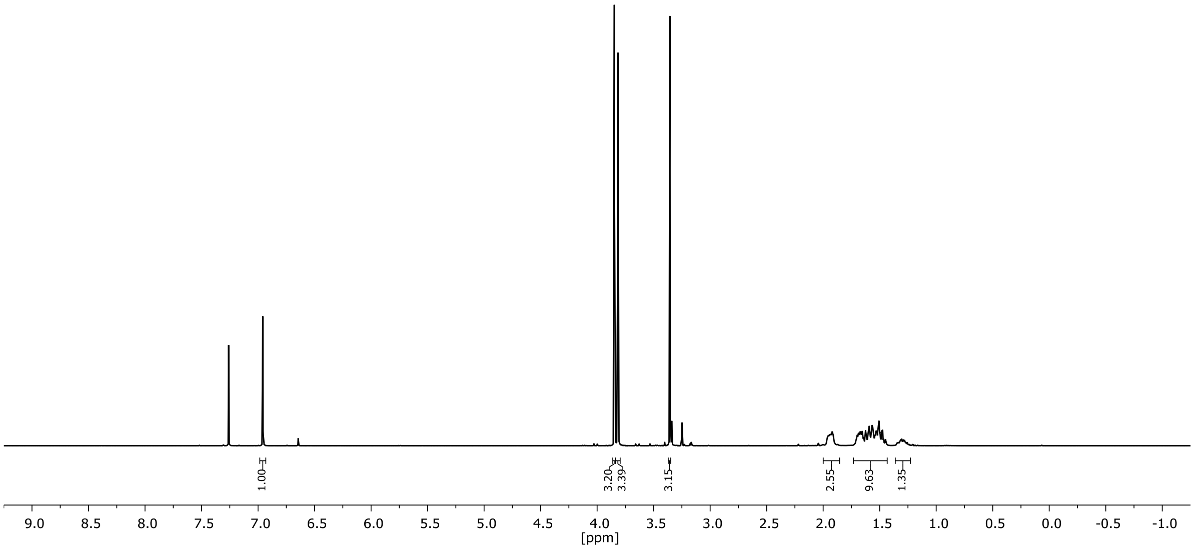
1.00
3.20
3.39
3.15
2.55
9.63
1.35
9.0 8.5 8.0 7.5 7.0 6.5 6.0 5.5 5.0 4.5 4.0 3.5 3.0 2.5 2.0 1.5 1.0 0.5 0.0 -0.5 -1.0
[ppm]

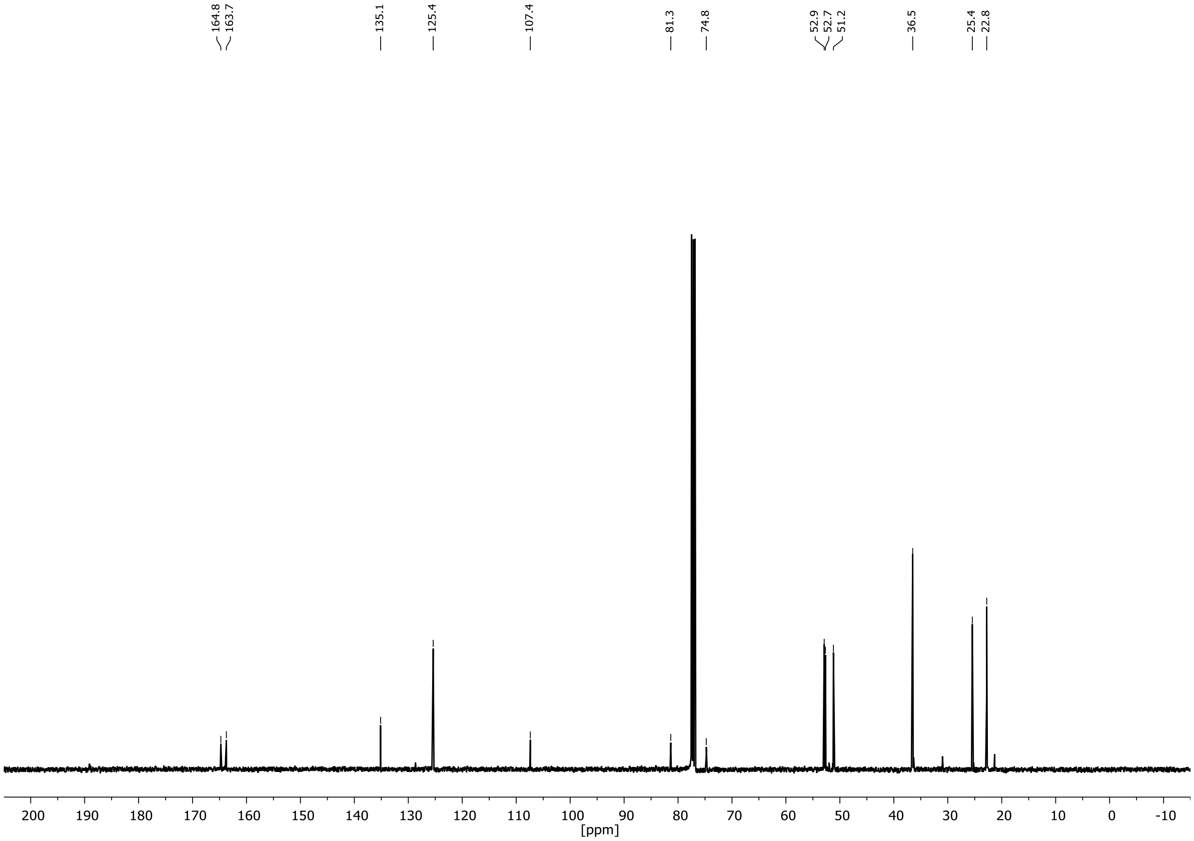
164.8
163.7
135.1
125.4
107.4
81.3
74.8
52.9
52.7
51.2
36.5
25.4
22.8
200 190 180 170 160 150 140 130 120 110 100 90 80 70 60 50 40 30 20 10 0 -10
[ppm]

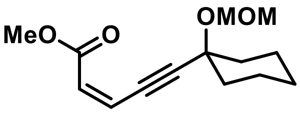
MeO
O
OMOM

S15

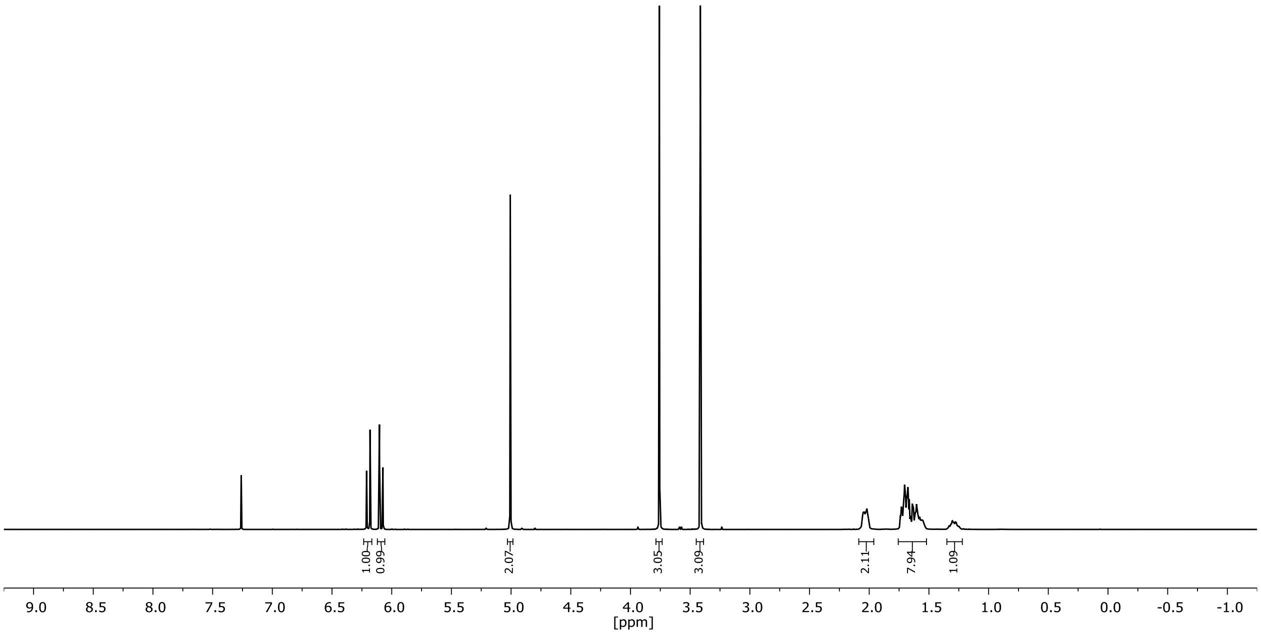
1.00
0.99
2.07
3.05
3.09
2.11
7.94
1.09
9.0 8.5 8.0 7.5 7.0 6.5 6.0 5.5 5.0 4.5 4.0 3.5 3.0 2.5 2.0 1.5 1.0 0.5 0.0 -0.5 -1.0
[ppm]

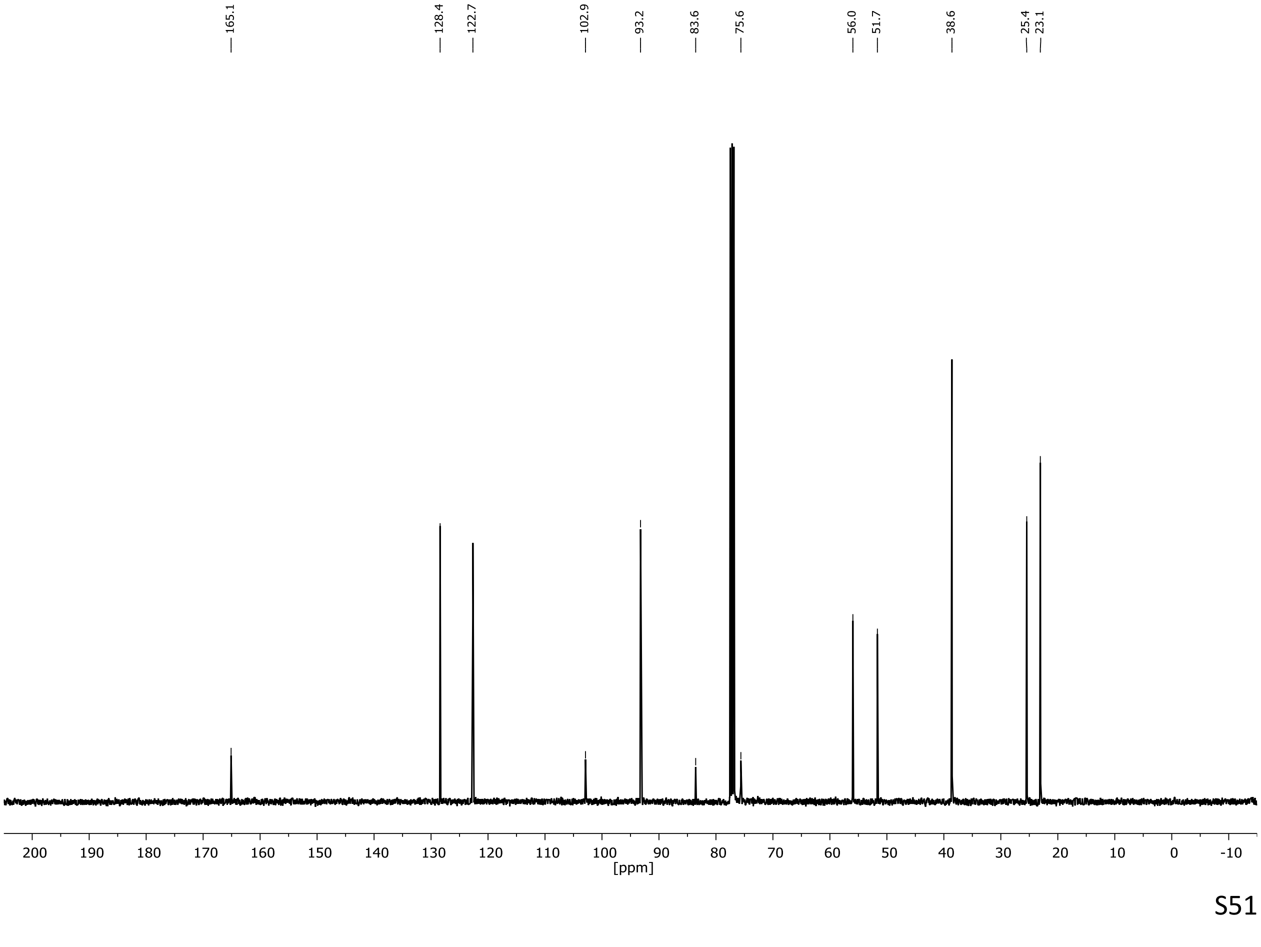
165.1
128.4
122.7
102.9
93.2
83.6
75.6
56.0
51.7
38.6
25.4
23.1
200 190 180 170 160 150 140 130 120 110 100 90 80 70 60 50 40 30 20 10 0 -10
[ppm]

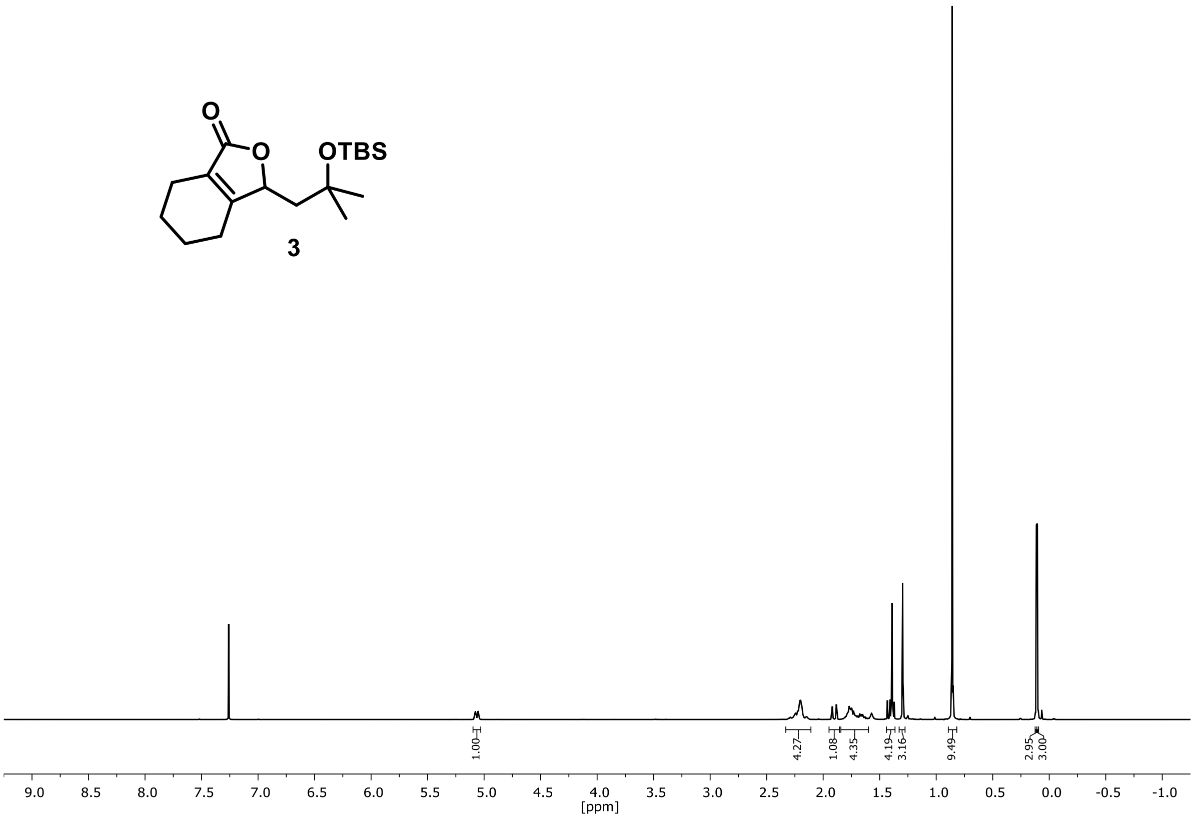
O
O
OTBS
3
1.00
4.27
1.08
4.35
4.19
3.16
9.49
2.95
3.00
9.0 8.5 8.0 7.5 7.0 6.5 6.0 5.5 5.0 4.5 4.0 3.5 3.0 2.5 2.0 1.5 1.0 0.5 0.0 -0.5 -1.0
[ppm]

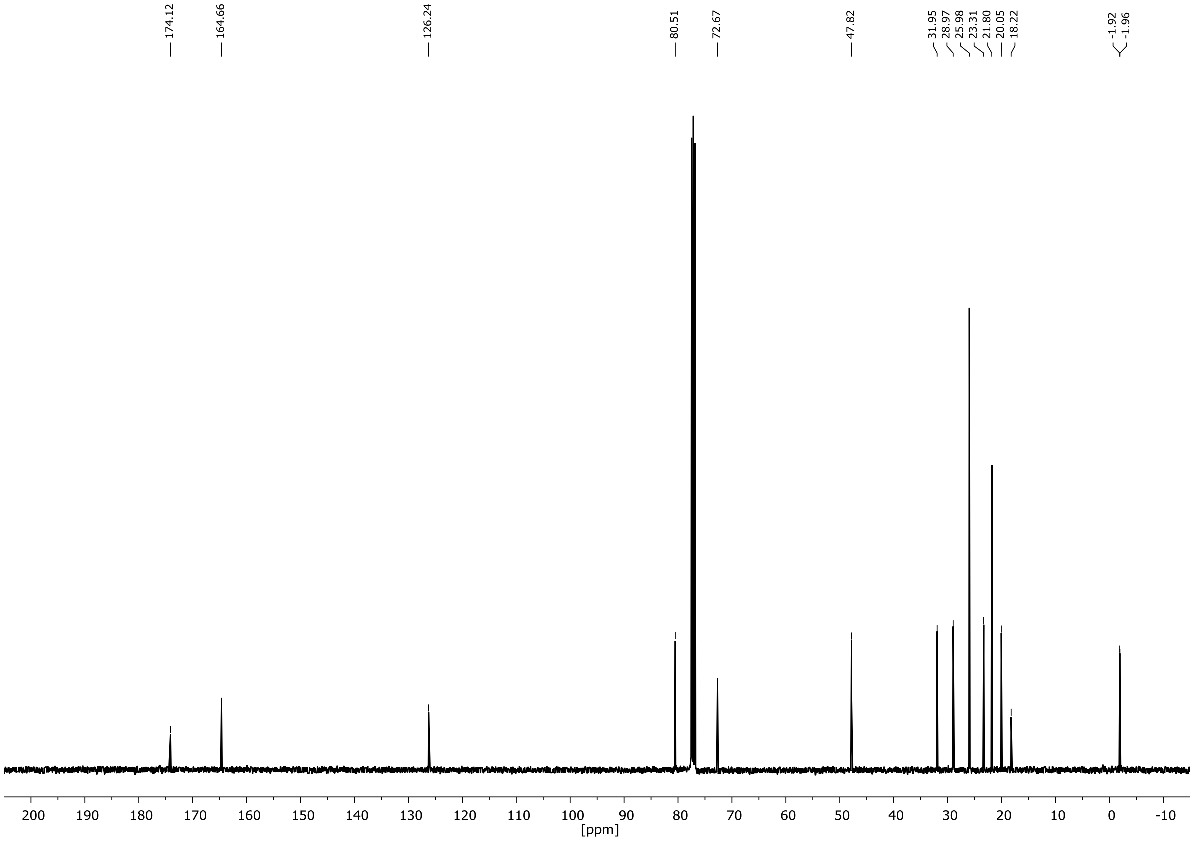
174.12
164.66
126.24
80.51
72.67
47.82
31.95
28.97
25.98
23.31
21.80
20.05
18.22
-1.92
-1.96
200 190 180 170 160 150 140 130 120 110 100 90 80 70 60 50 40 30 20 10 0 -10
[ppm]

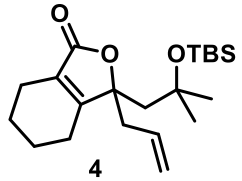

4

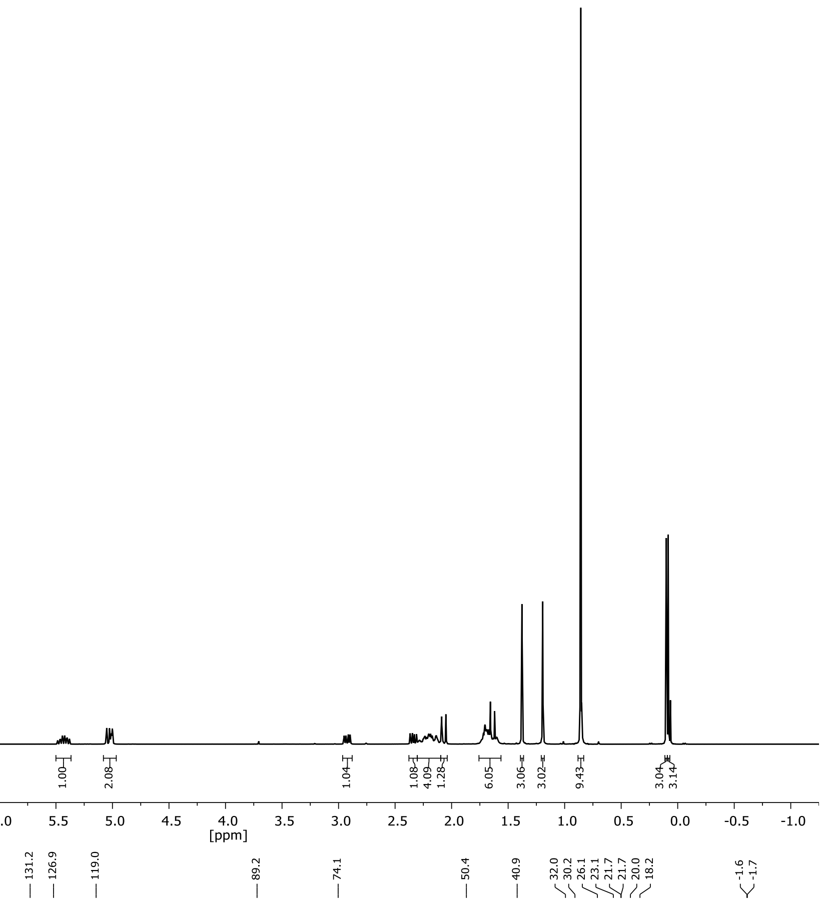

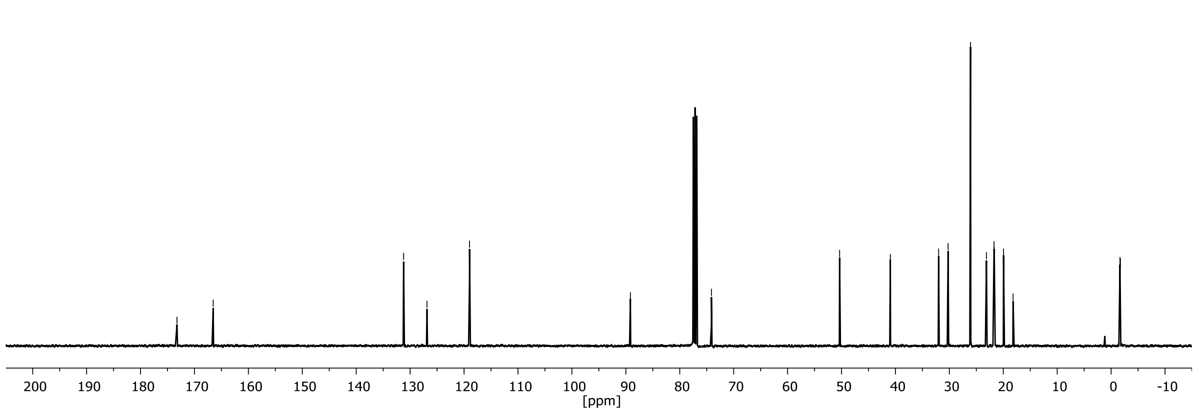

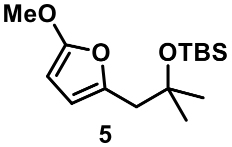
MeO
O
OTBS

5

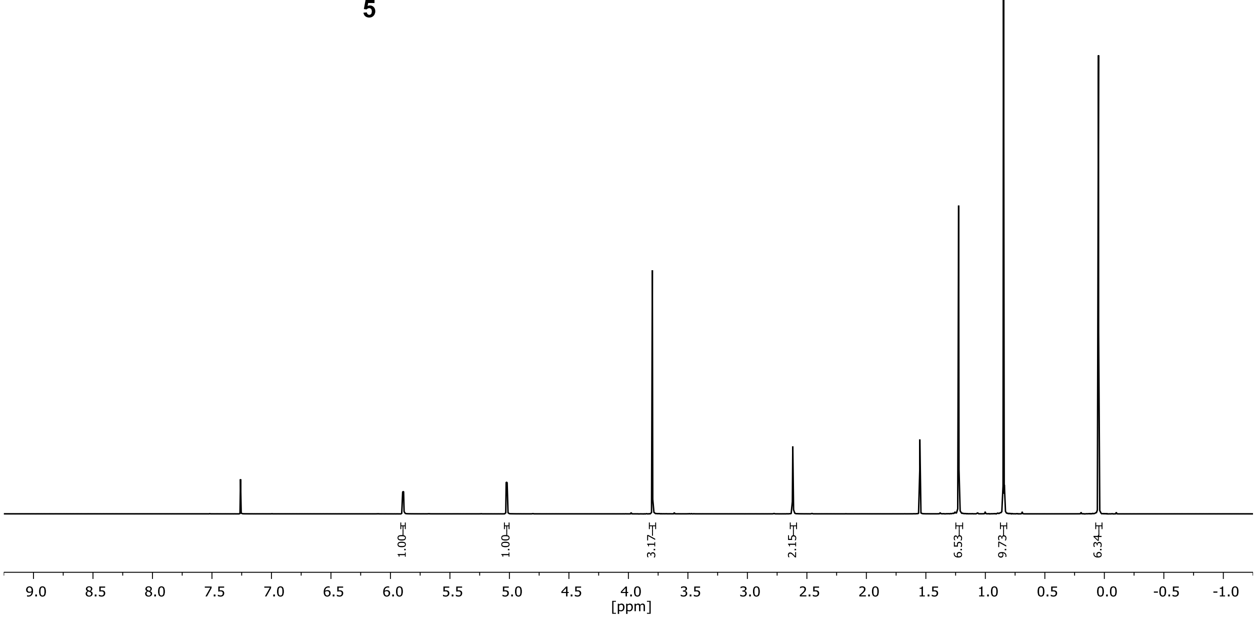
1.00
1.00
3.17
2.15
6.53
9.73
6.34
9.0 8.5 8.0 7.5 7.0 6.5 6.0 5.5 5.0 4.5 4.0 3.5 3.0 2.5 2.0 1.5 1.0 0.5 0.0 -0.5 -1.0
[ppm]

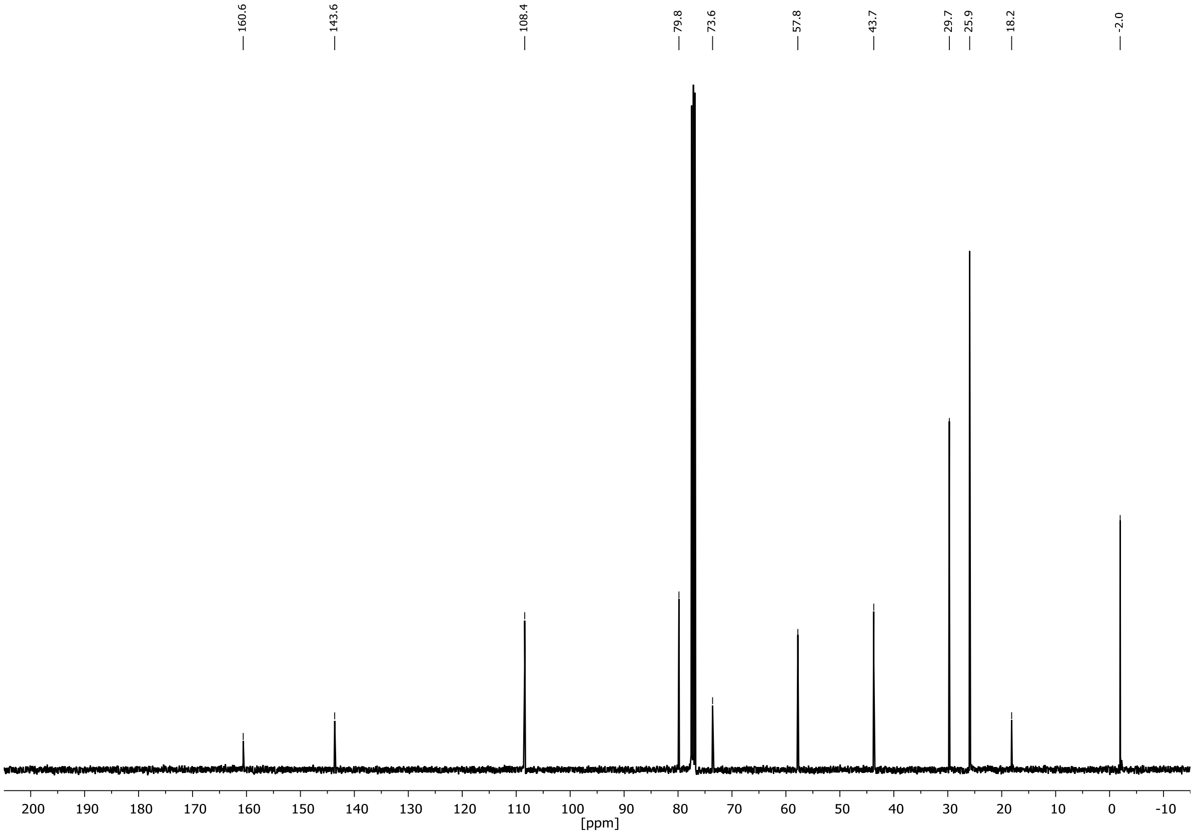
160.6
143.6
108.4
79.8
73.6
57.8
43.7
29.7
25.9
18.2
-2.0
200 190 180 170 160 150 140 130 120 110 100 90 80 70 60 50 40 30 20 10 0 -10
[ppm]

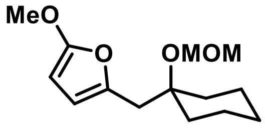
MeO
O
OMOM

6

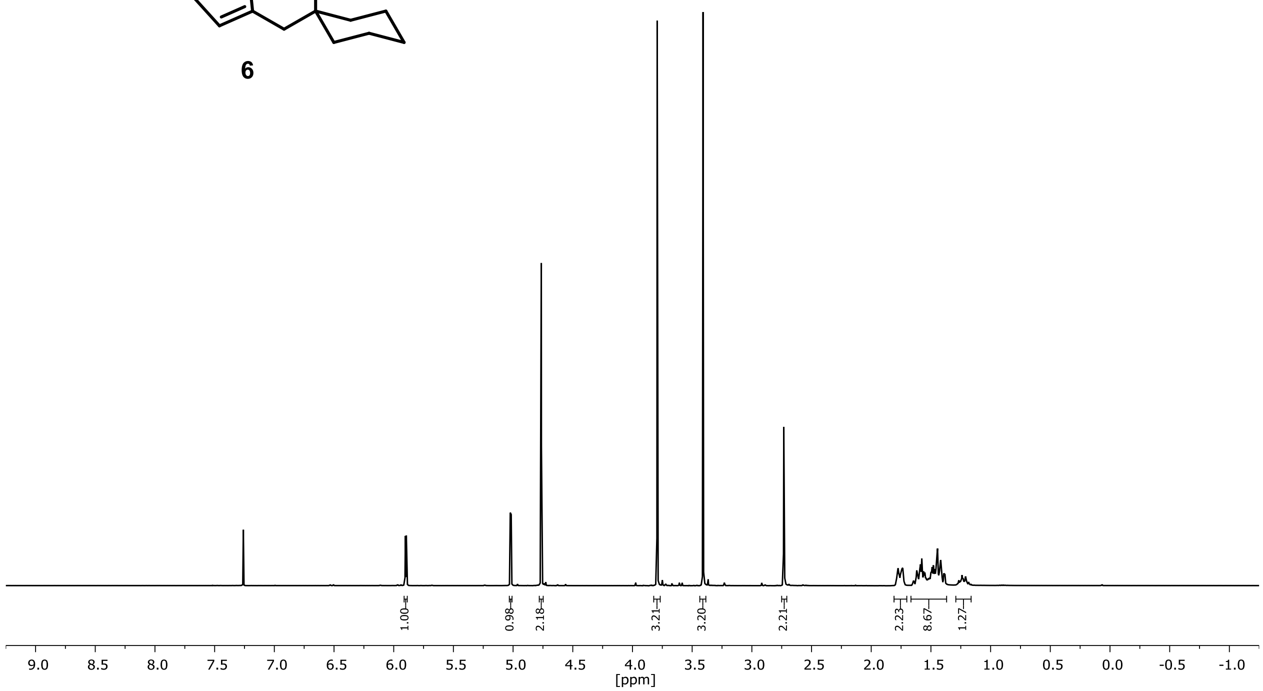
1.00
0.98
2.18
3.21
3.20
2.21
2.23
8.67
1.27
9.0 8.5 8.0 7.5 7.0 6.5 6.0 5.5 5.0 4.5 4.0 3.5 3.0 2.5 2.0 1.5 1.0 0.5 0.0 -0.5 -1.0
[ppm]

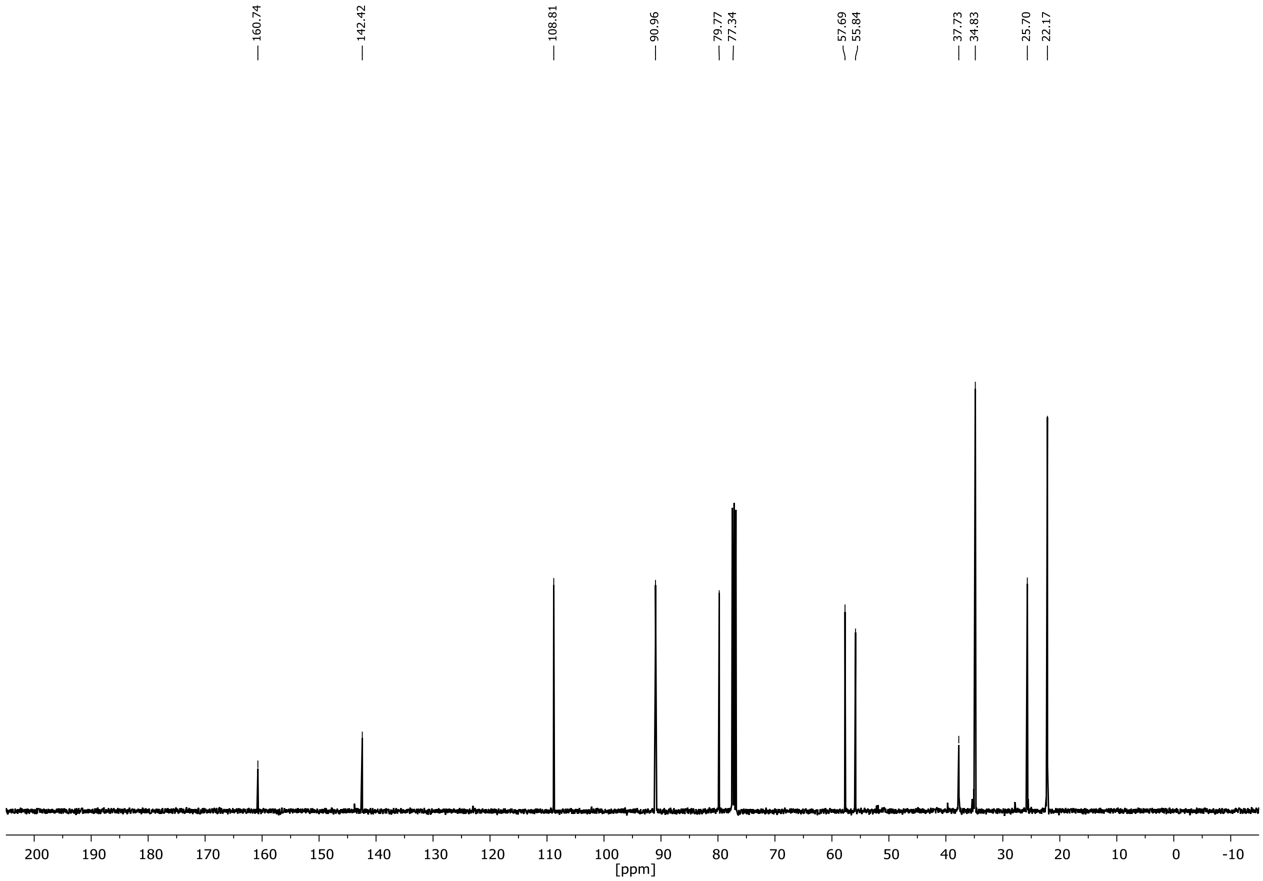
160.74
142.42
108.81
90.96
79.77
77.34
57.69
55.84
37.73
34.83
25.70
22.17
200 190 180 170 160 150 140 130 120 110 100 90 80 70 60 50 40 30 20 10 0 -10
[ppm]

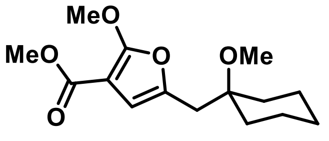
MeO
MeO
O
OMe
O

7

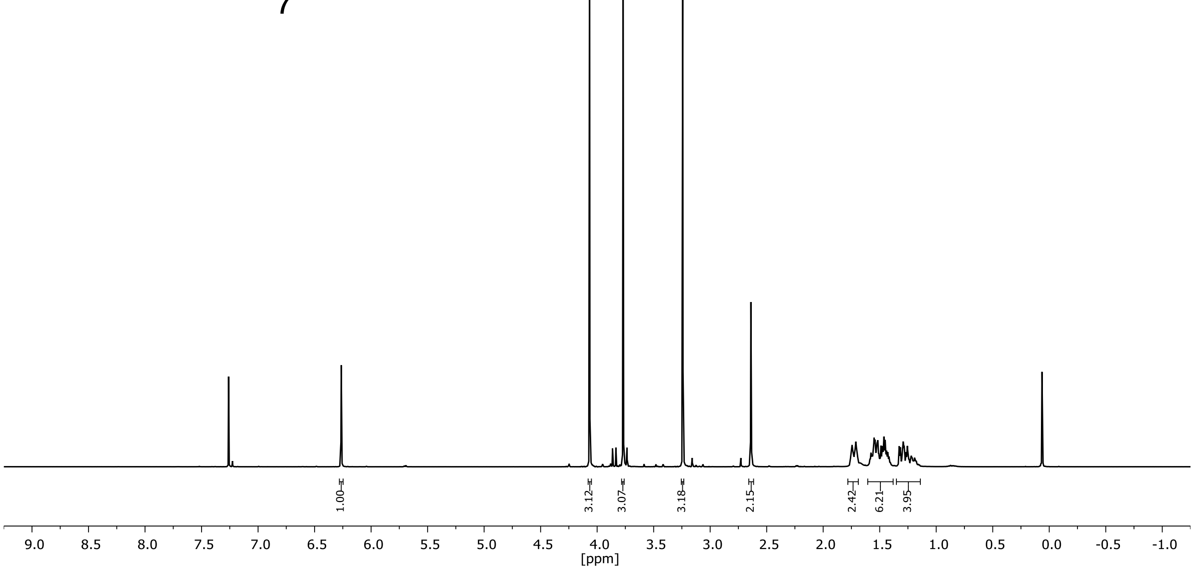
1.00
3.12
3.07
3.18
2.15
2.42
6.21
3.95
9.0 8.5 8.0 7.5 7.0 6.5 6.0 5.5 5.0 4.5 4.0 3.5 3.0 2.5 2.0 1.5 1.0 0.5 0.0 -0.5 -1.0
[ppm]

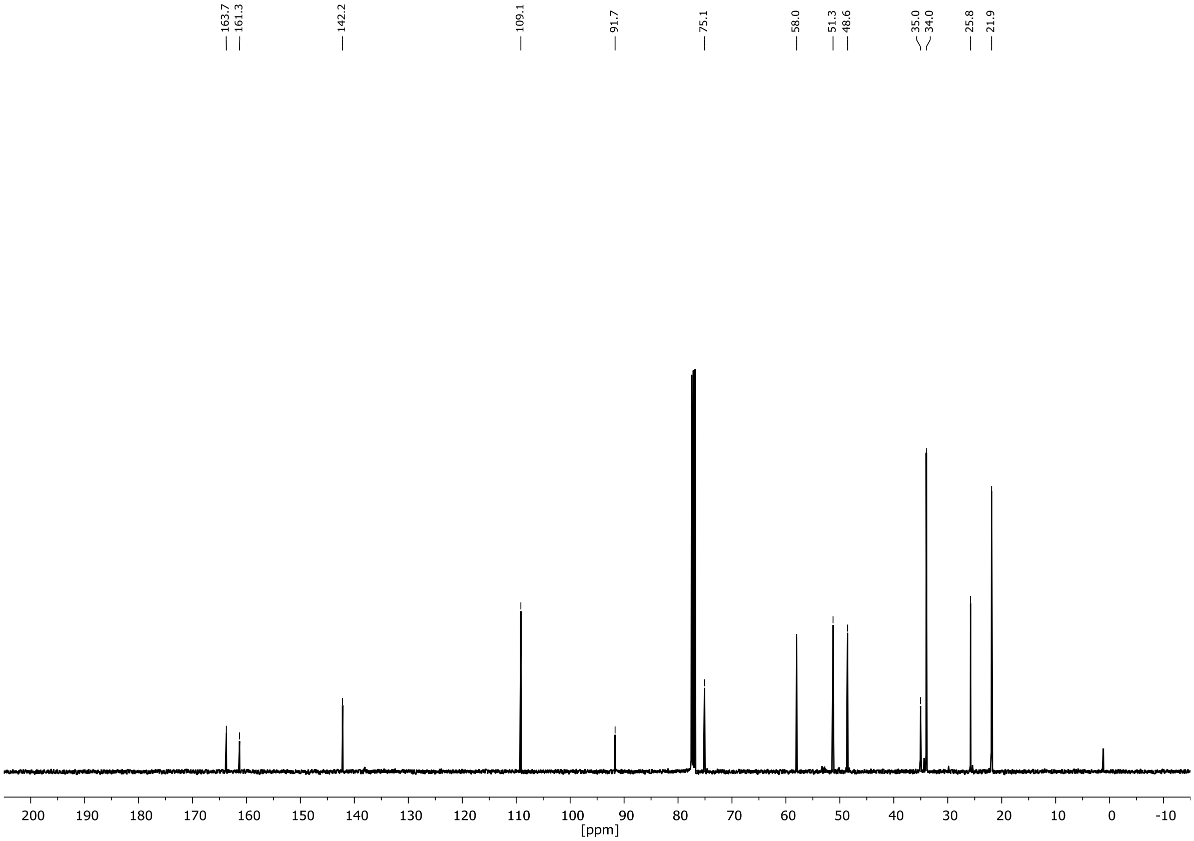
163.7
161.3
142.2
109.1
91.7
75.1
58.0
51.3
48.6
35.0
34.0
25.8
21.9
200 190 180 170 160 150 140 130 120 110 100 90 80 70 60 50 40 30 20 10 0 -10
[ppm]

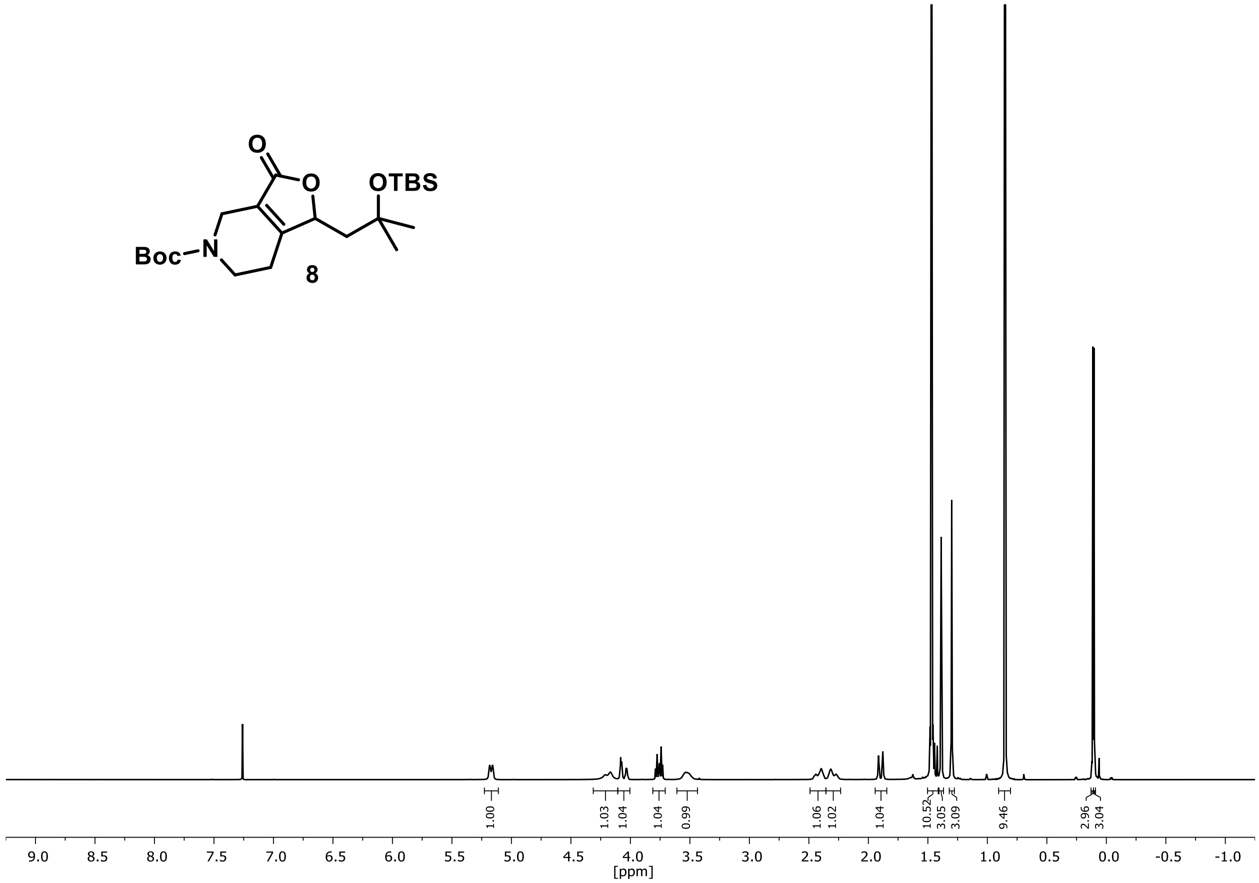
O
O
OTBS
Boc
N
8
1.00
1.03
1.04
1.04
0.99
1.06
1.02
1.04
10.52
3.05
3.09
9.46
2.96
3.04
9.0 8.5 8.0 7.5 7.0 6.5 6.0 5.5 5.0 4.5 4.0 3.5 3.0 2.5 2.0 1.5 1.0 0.5 0.0 -0.5 -1.0
[ppm]

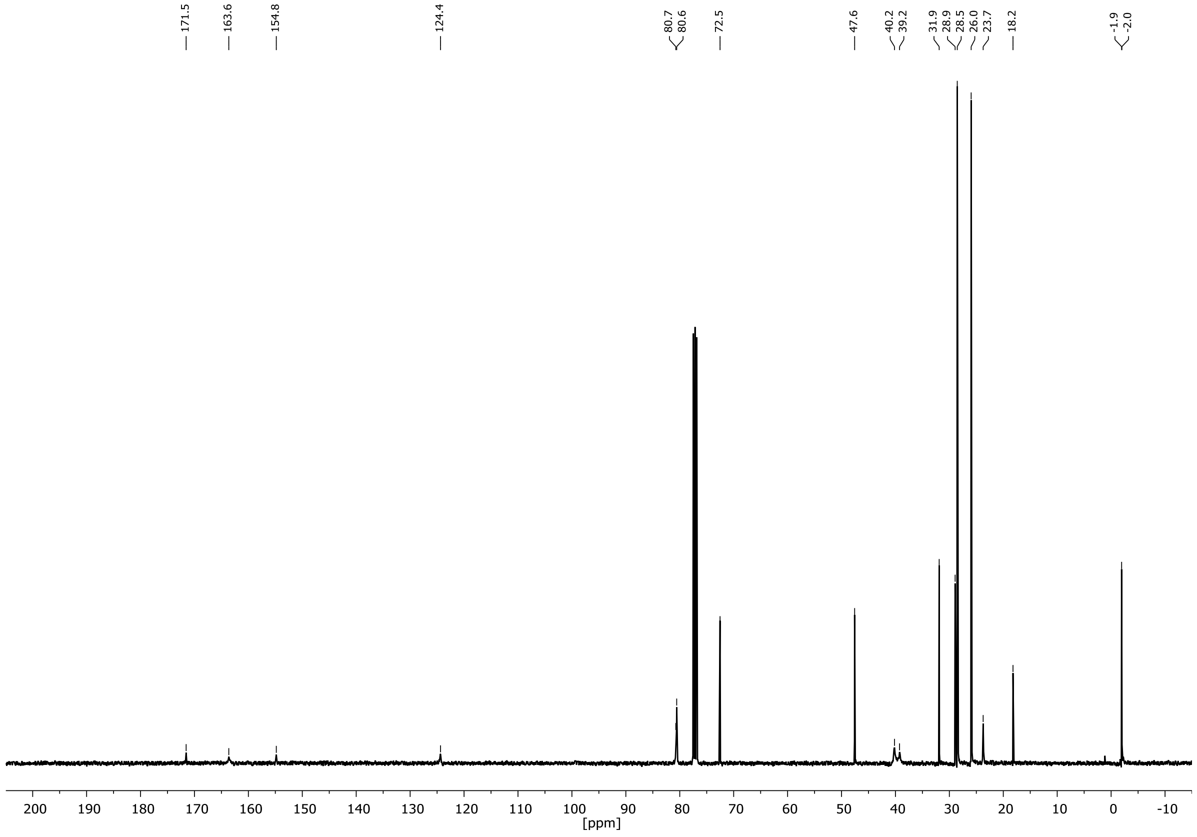
171.5
163.6
154.8
124.4
80.7
80.6
72.5
47.6
40.2
39.2
31.9
28.9
28.5
26.0
23.7
18.2
-1.9
-2.0
200 190 180 170 160 150 140 130 120 110 100 90 80 70 60 50 40 30 20 10 0 -10
[ppm]

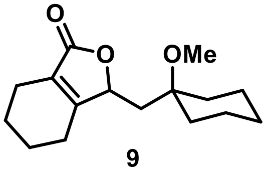
O
O
OMe

9

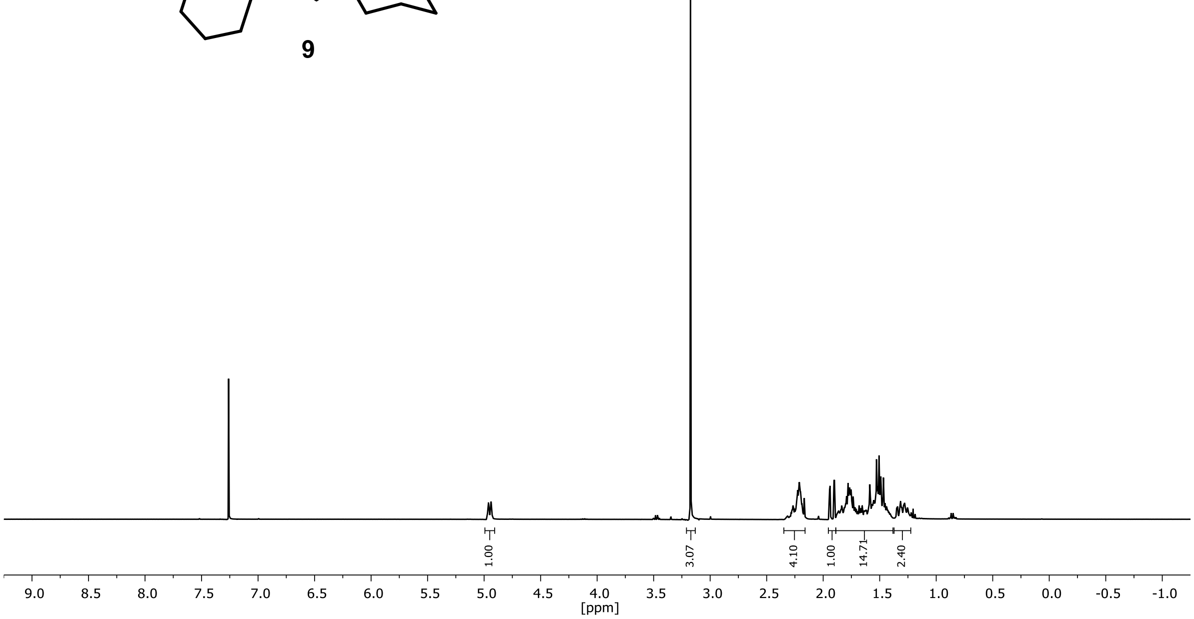
1.00
3.07
4.10
1.00
14.71
2.40
9.0 8.5 8.0 7.5 7.0 6.5 6.0 5.5 5.0 4.5 4.0 3.5 3.0 2.5 2.0 1.5 1.0 0.5 0.0 -0.5 -1.0
[ppm]

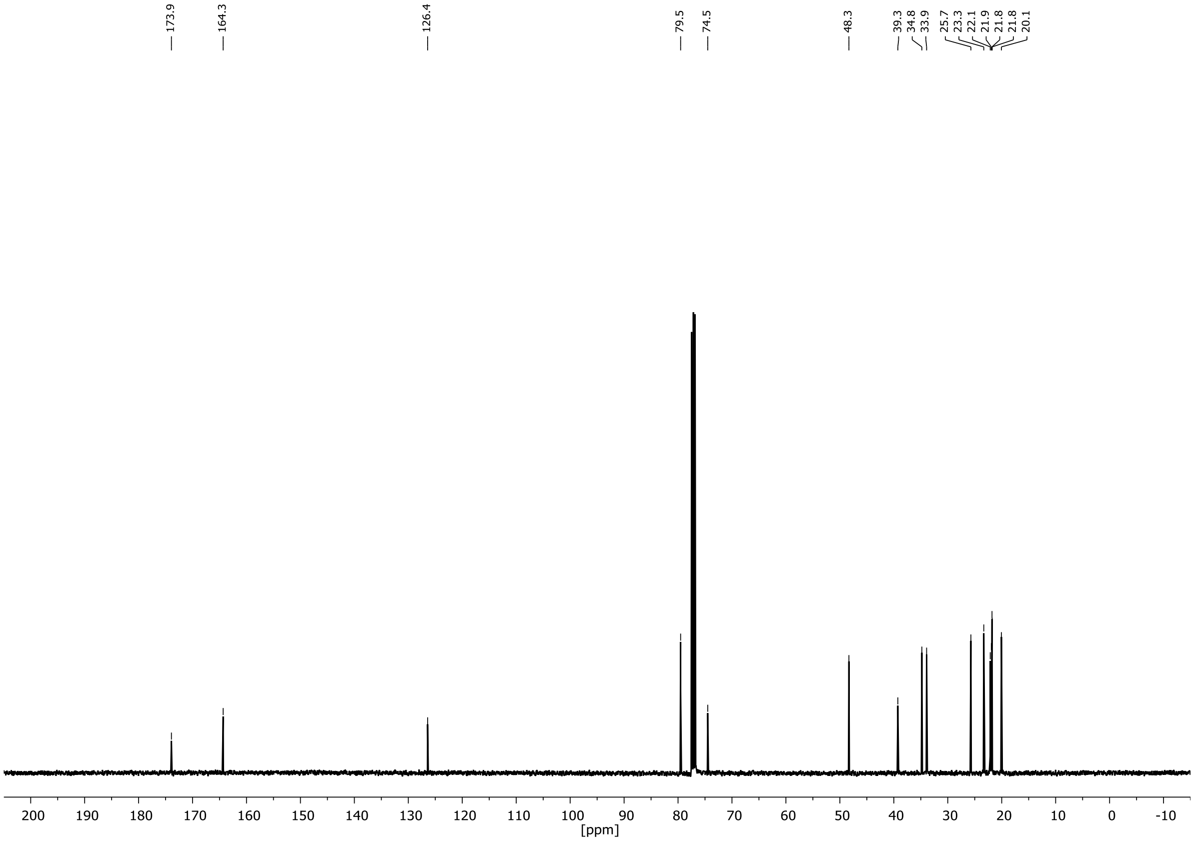
173.9
164.3
126.4
79.5
74.5
48.3
39.3
34.8
33.9
25.7
23.3
22.1
21.9
21.8
21.8
20.1
200 190 180 170 160 150 140 130 120 110 100 90 80 70 60 50 40 30 20 10 0 -10
[ppm]

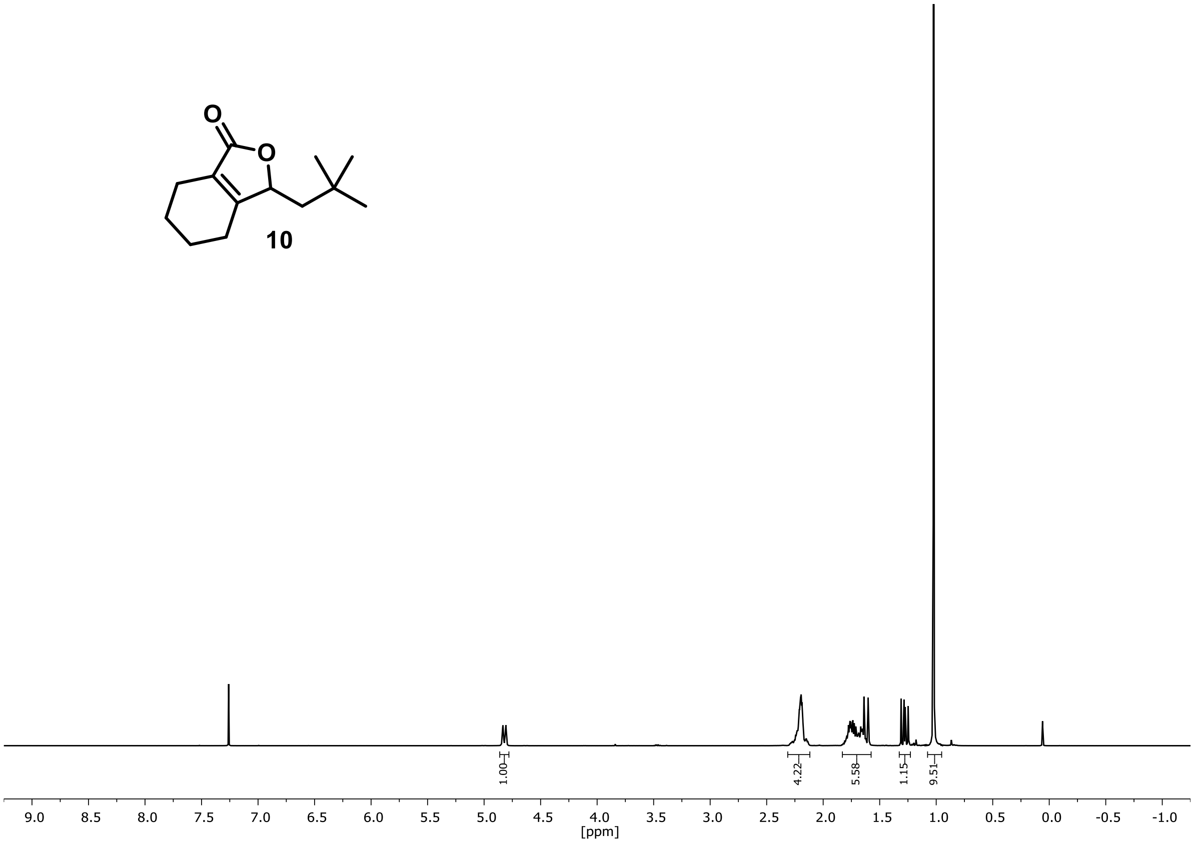
O
O
10
1.00
4.22
5.58
1.15
9.51
9.0 8.5 8.0 7.5 7.0 6.5 6.0 5.5 5.0 4.5 4.0 3.5 3.0 2.5 2.0 1.5 1.0 0.5 0.0 -0.5 -1.0
[ppm]

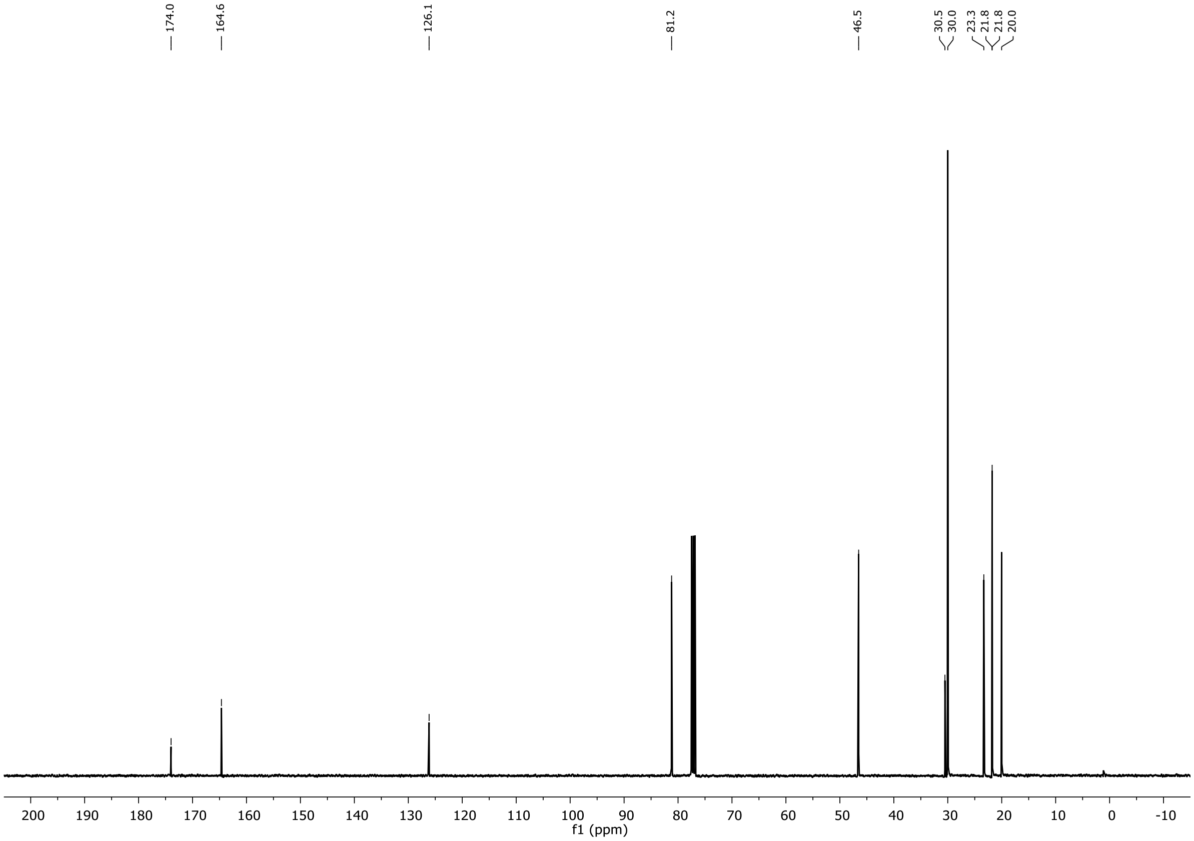
174.0
164.6
126.1
81.2
46.5
30.5
30.0
23.3
21.8
21.8
20.0
200 190 180 170 160 150 140 130 120 110 100 90 80 70 60 50 40 30 20 10 0 -10
f1 (ppm)

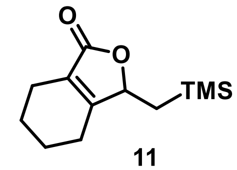
O
O
TMS

11

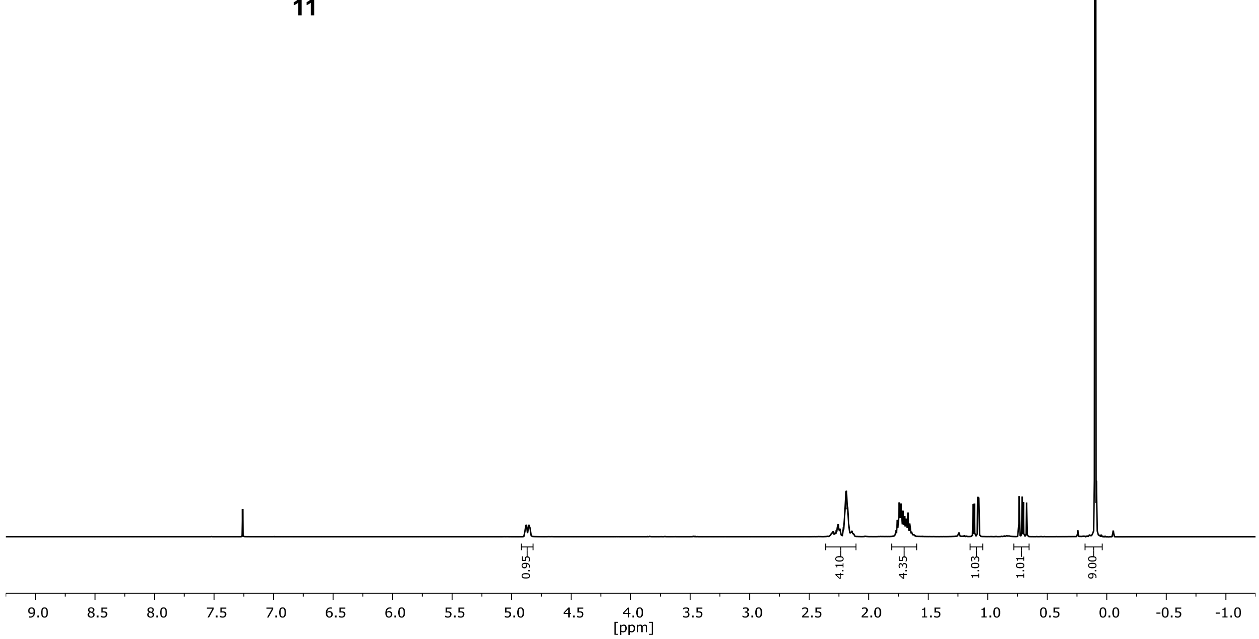
0.95
4.10
4.35
1.03
1.01
9.00
9.0 8.5 8.0 7.5 7.0 6.5 6.0 5.5 5.0 4.5 4.0 3.5 3.0 2.5 2.0 1.5 1.0 0.5 0.0 -0.5 -1.0
[ppm]

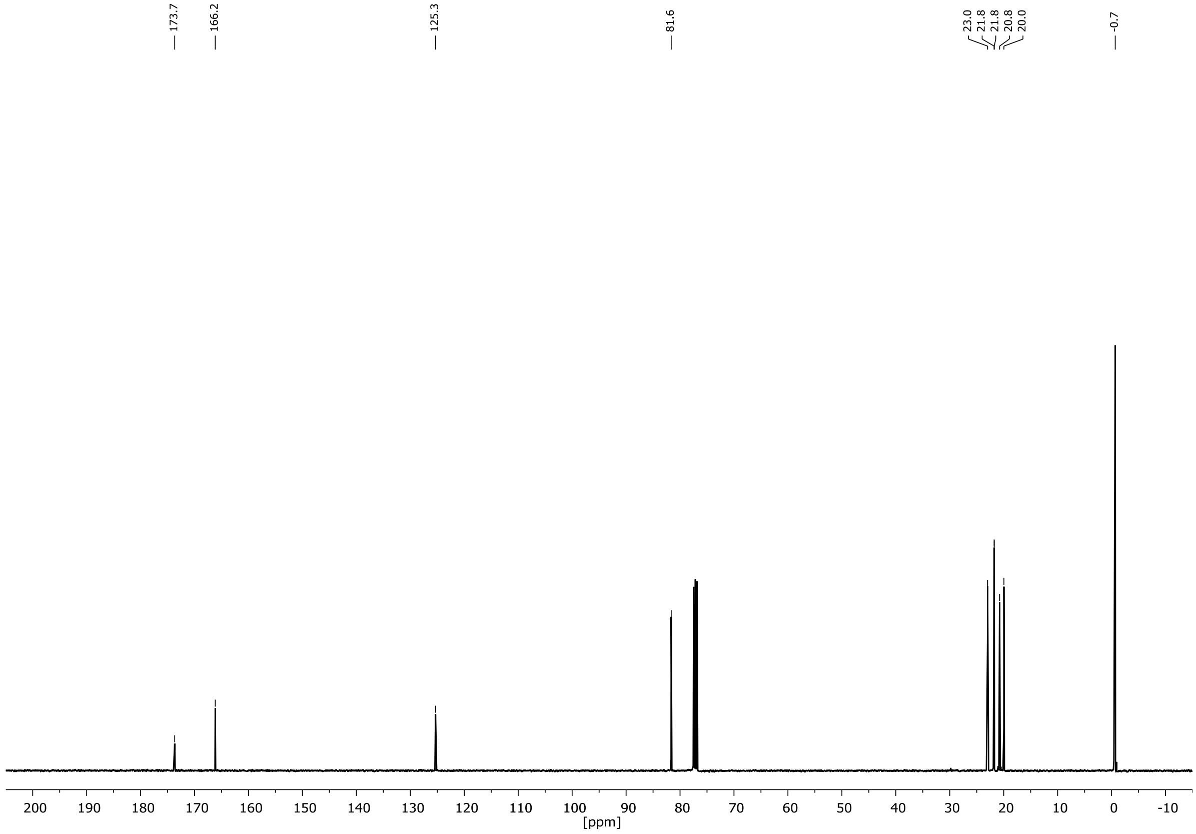
173.7
166.2
125.3
81.6
23.0
21.8
21.8
20.8
20.0
-0.7
200 190 180 170 160 150 140 130 120 110 100 90 80 70 60 50 40 30 20 10 0 -10
[ppm]

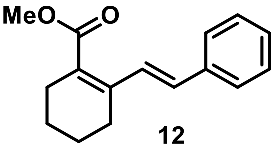

12

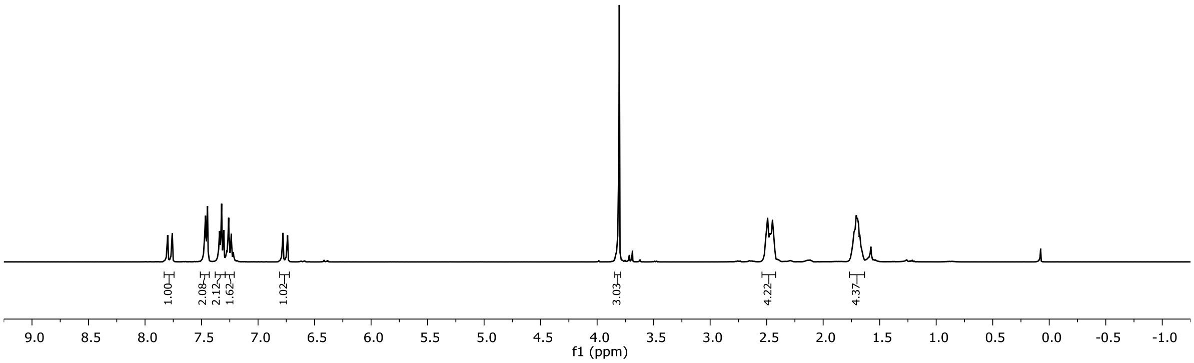
1.00
2.08
2.12
1.62
1.02
3.03
4.22
4.37
9.0 8.5 8.0 7.5 7.0 6.5 6.0 5.5 5.0 4.5 4.0 3.5 3.0 2.5 2.0 1.5 1.0 0.5 0.0 -0.5 -1.0
f1 (ppm)

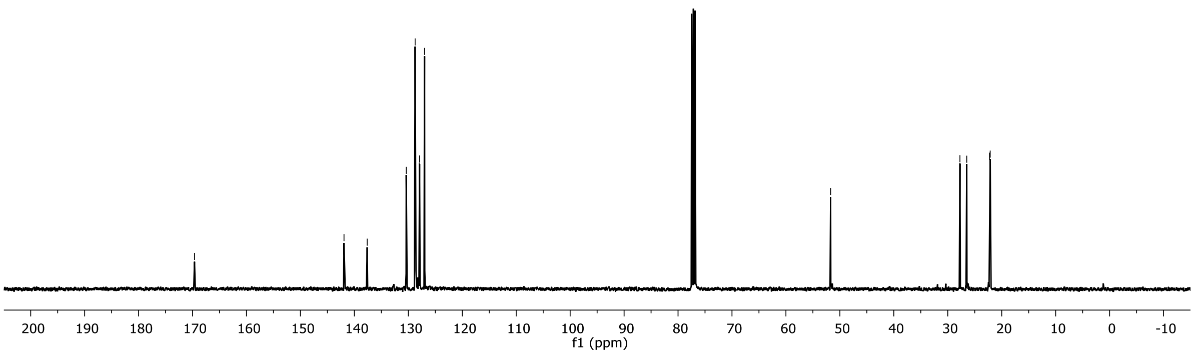
169.6
141.9
137.6
130.4
128.7
128.0
127.9
127.0
51.7
27.7
26.5
22.3
22.1
200 190 180 170 160 150 140 130 120 110 100 90 80 70 60 50 40 30 20 10 0 -10
f1 (ppm)

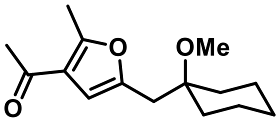
OMe
O
O

16

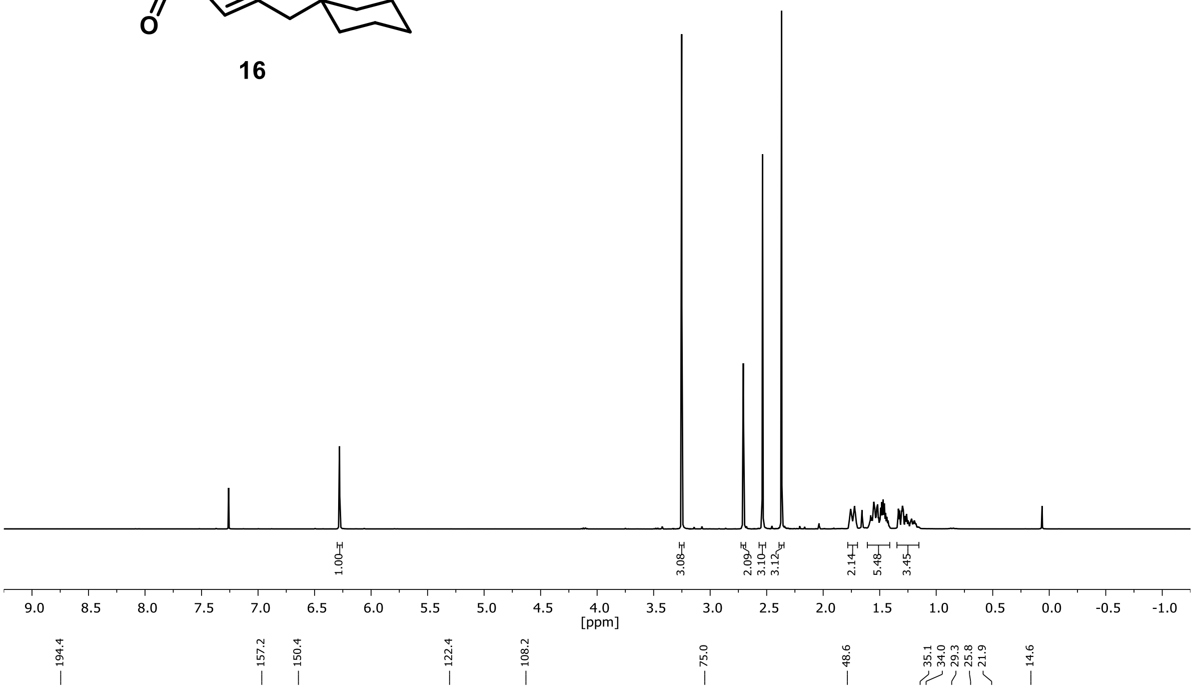
1.00
3.08
2.09
3.10
3.12
2.14
5.48
3.45
9.0 8.5 8.0 7.5 7.0 6.5 6.0 5.5 5.0 4.5 4.0 3.5 3.0 2.5 2.0 1.5 1.0 0.5 0.0 -0.5 -1.0
[ppm]

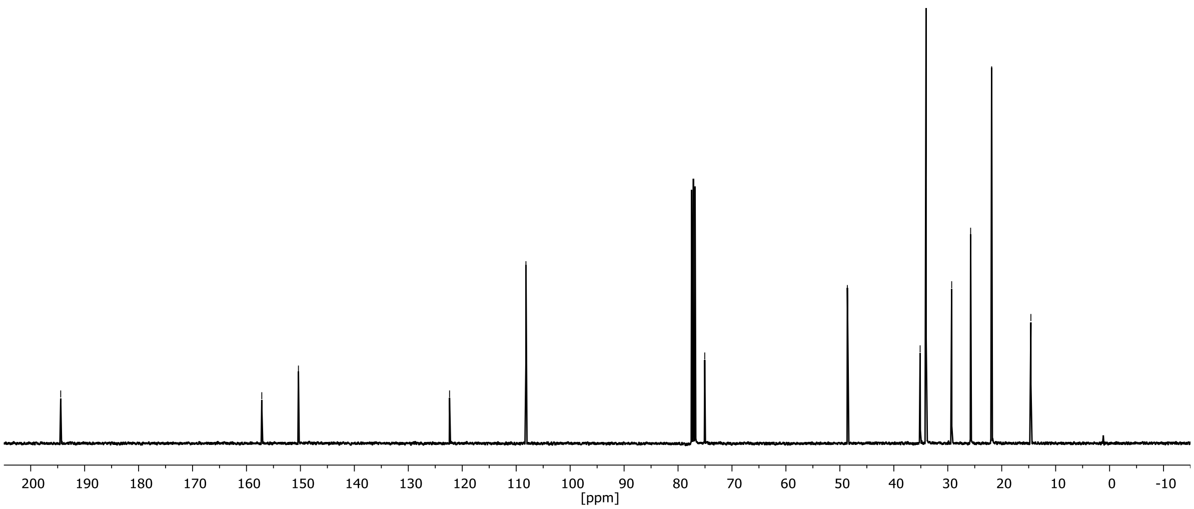
194.4
157.2
150.4
122.4
108.2
75.0
48.6
35.1
34.0
29.3
25.8
21.9
14.6
200 190 180 170 160 150 140 130 120 110 100 90 80 70 60 50 40 30 20 10 0 -10
[ppm]

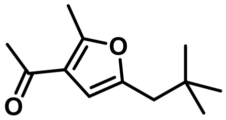
O
O

23a

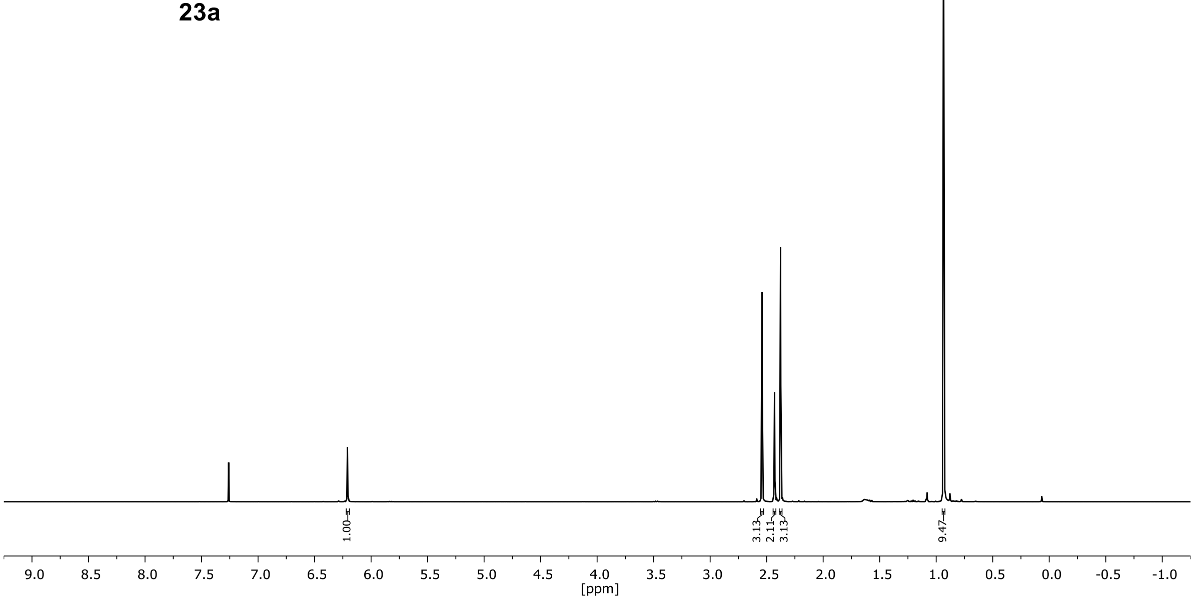
1.00
3.13
2.11
3.13
9.47
[ppm]

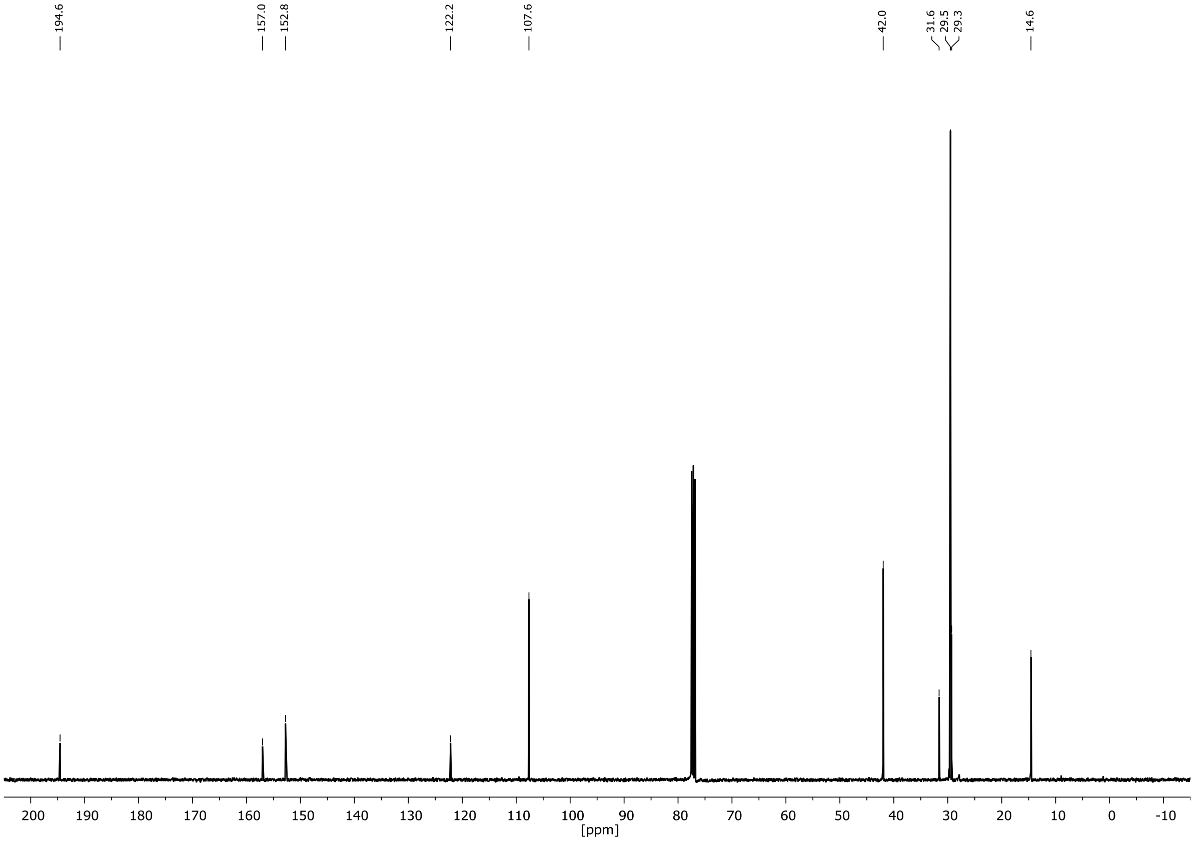
194.6
157.0
152.8
122.2
107.6
42.0
31.6
29.5
29.3
14.6
[ppm]

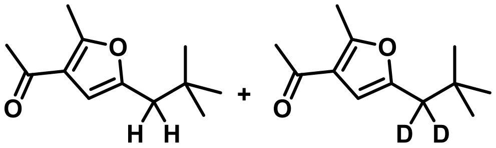

23a (58.6 %) **[D_2-23a] (40.7 %)**

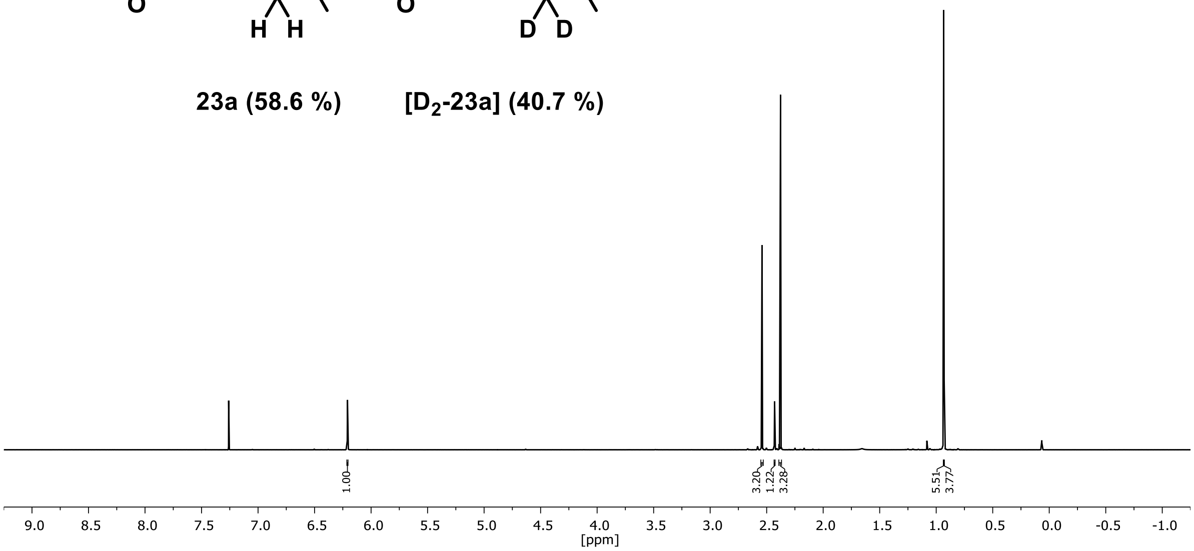

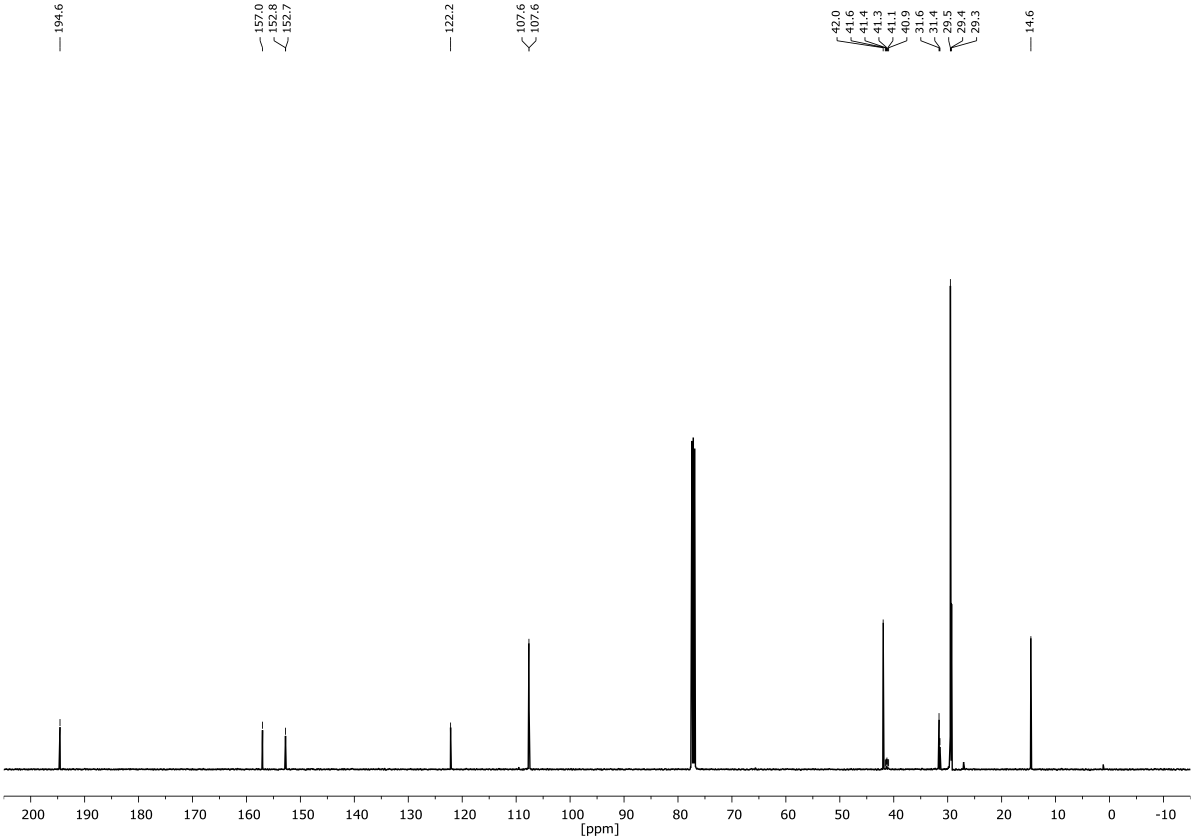

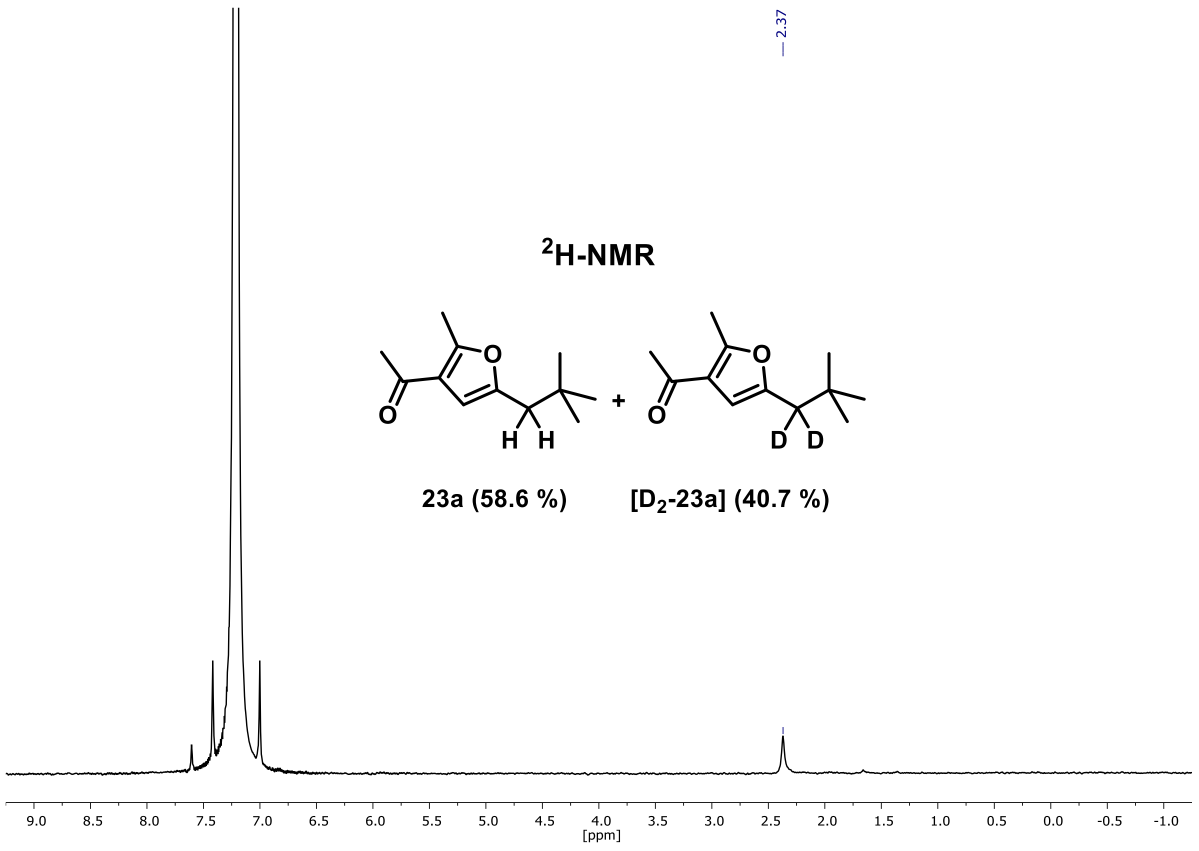

2.37
²H-NMR
O
H H
+
O
D D
23a (58.6 %)
[D2-23a] (40.7 %)
9.0 8.5 8.0 7.5 7.0 6.5 6.0 5.5 5.0 4.5 4.0 3.5 3.0 2.5 2.0 1.5 1.0 0.5 0.0 -0.5 -1.0
[ppm]

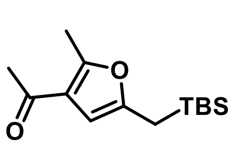
TBS
O
O

23b

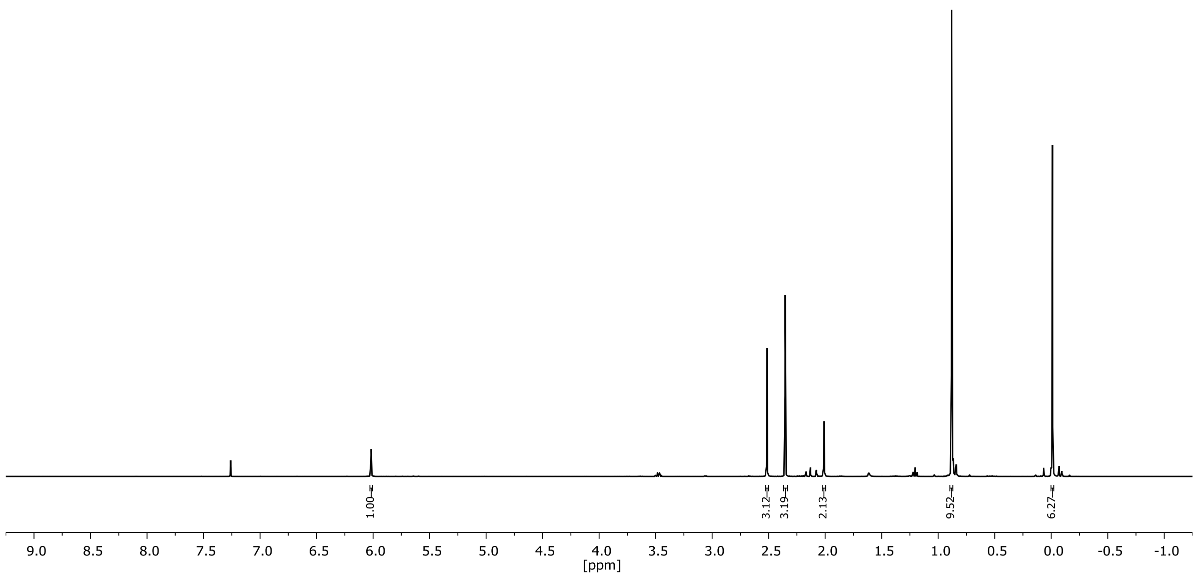
1.00
3.12
3.19
2.13
9.52
6.27
9.0 8.5 8.0 7.5 7.0 6.5 6.0 5.5 5.0 4.5 4.0 3.5 3.0 2.5 2.0 1.5 1.0 0.5 0.0 -0.5 -1.0
[ppm]

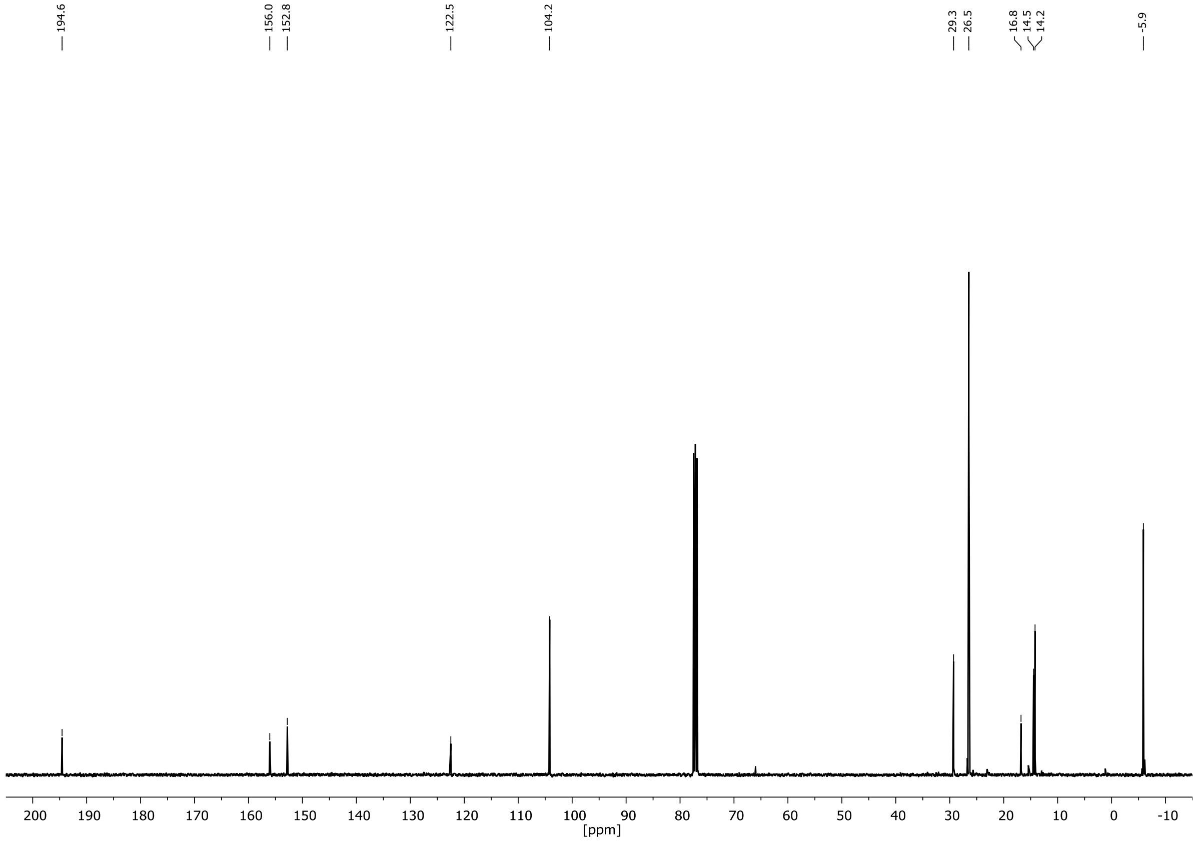
194.6
156.0
152.8
122.5
104.2
29.3
26.5
16.8
14.5
14.2
-5.9
200 190 180 170 160 150 140 130 120 110 100 90 80 70 60 50 40 30 20 10 0 -10
[ppm]

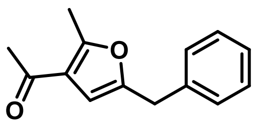

23c

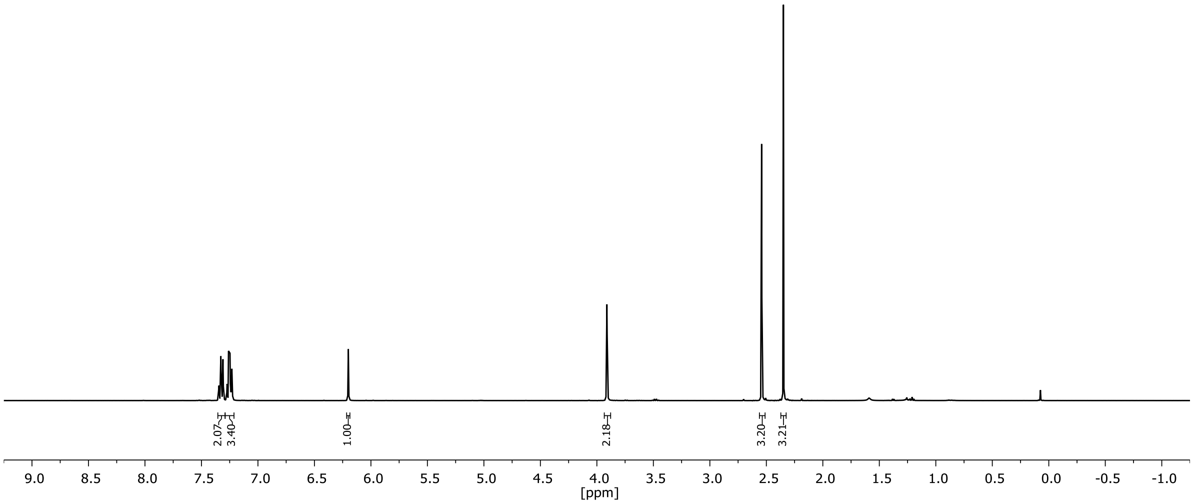
2.07
3.40
1.00
2.18
3.20
3.21
9.0 8.5 8.0 7.5 7.0 6.5 6.0 5.5 5.0 4.5 4.0 3.5 3.0 2.5 2.0 1.5 1.0 0.5 0.0 -0.5 -1.0
[ppm]

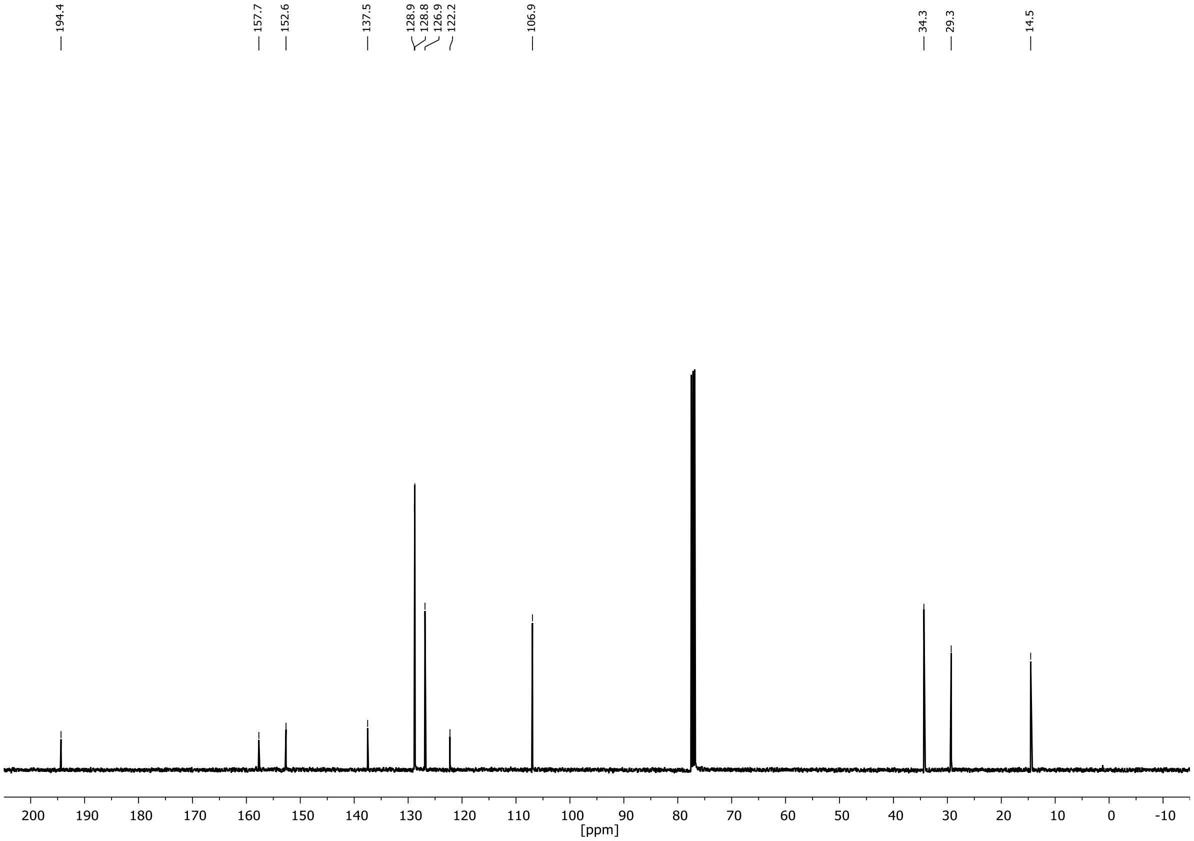
194.4
157.7
152.6
137.5
128.9
128.8
126.9
122.2
106.9
34.3
29.3
14.5
200 190 180 170 160 150 140 130 120 110 100 90 80 70 60 50 40 30 20 10 0 -10
[ppm]

24

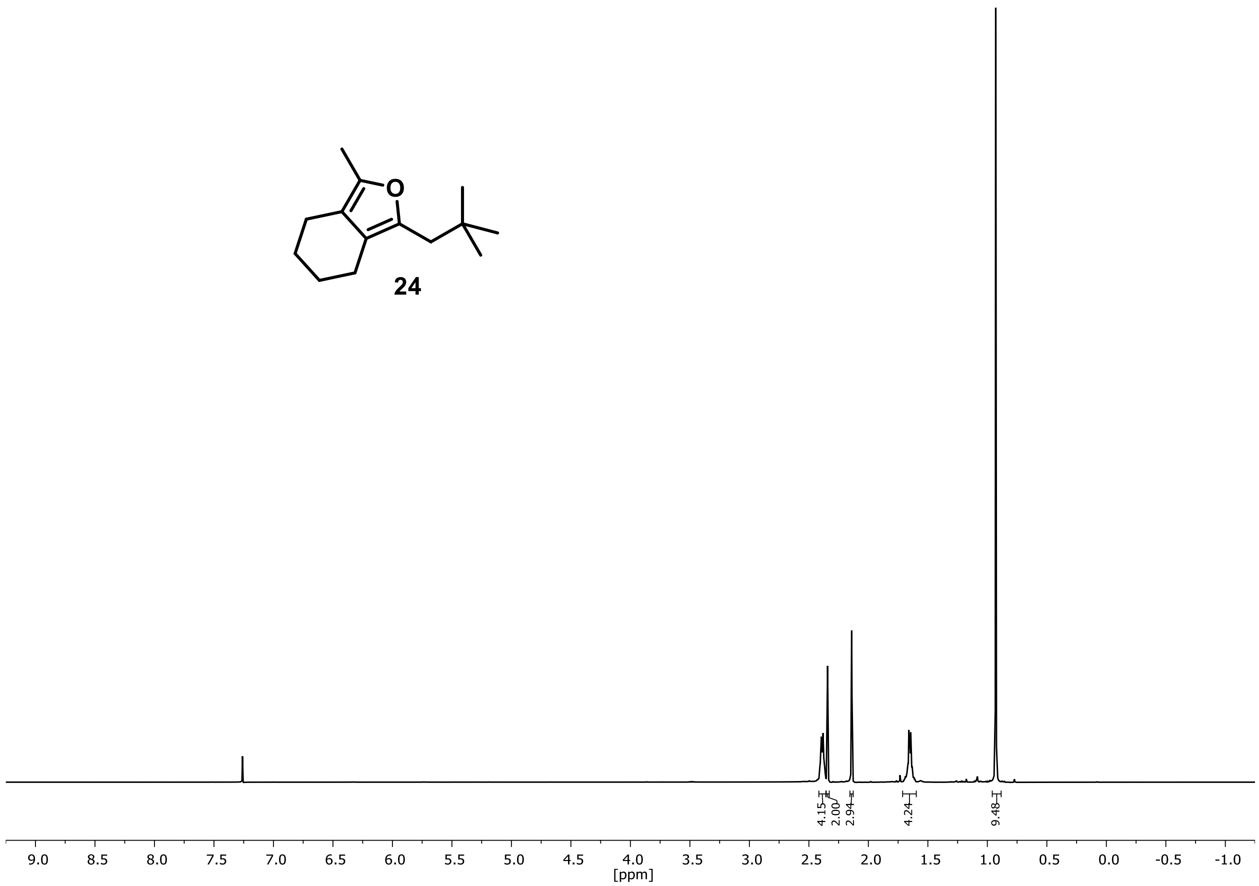

4.15
2.00
2.94
4.24
9.48
9.0
8.5
8.0
7.5
7.0
6.5
6.0
5.5
5.0
4.5
4.0
3.5
3.0
2.5
2.0
1.5
1.0
0.5
0.0
-0.5
-1.0
[ppm]

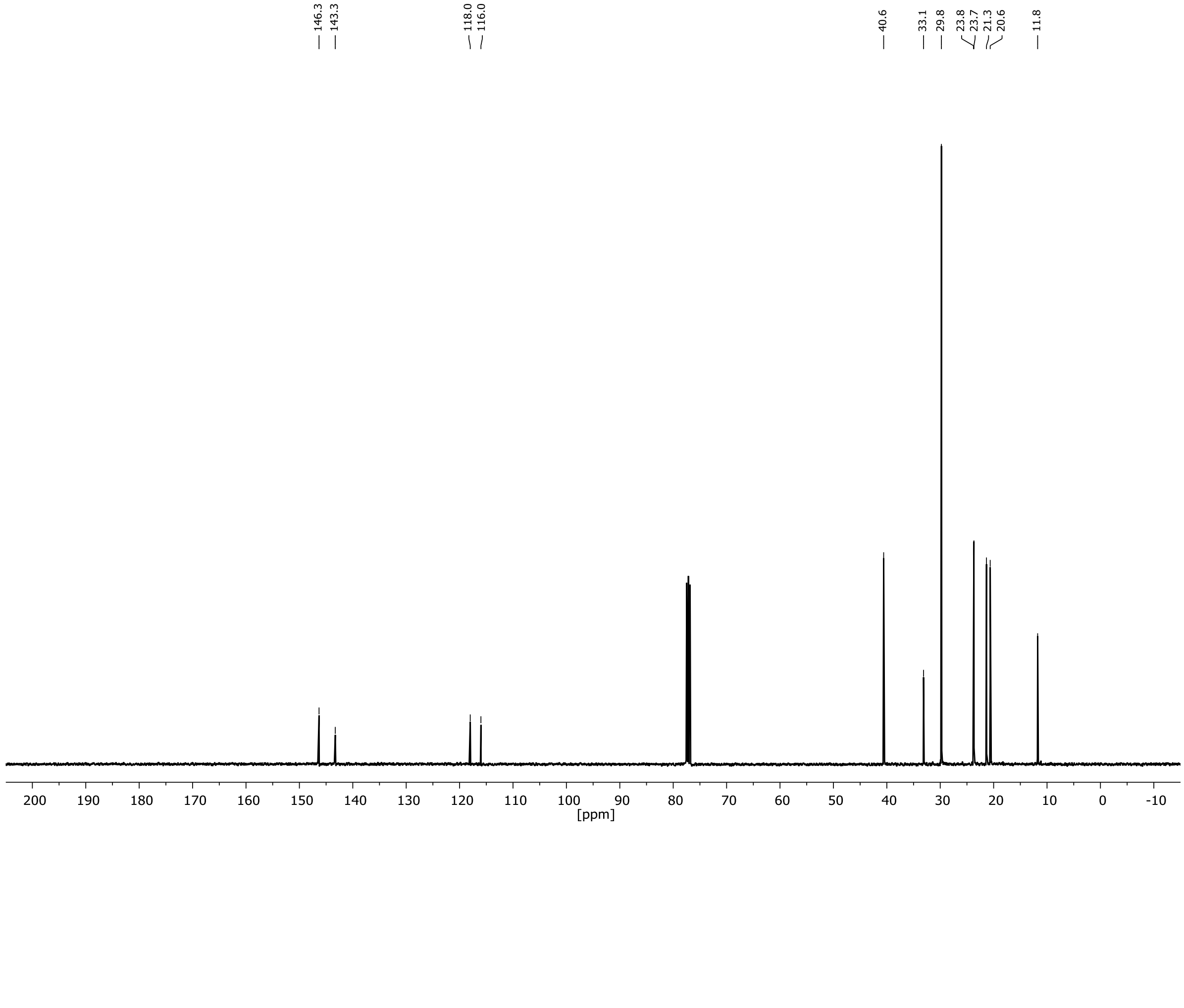

146.3
143.3
118.0
116.0
40.6
33.1
29.8
23.8
23.7
21.3
20.6
11.8
200
190
180
170
160
150
140
130
120
110
100
90
80
70
60
50
40
30
20
10
0
-10
[ppm]

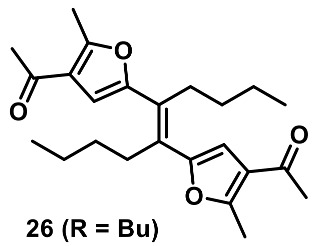
O
O
O
O

26 (R = Bu)

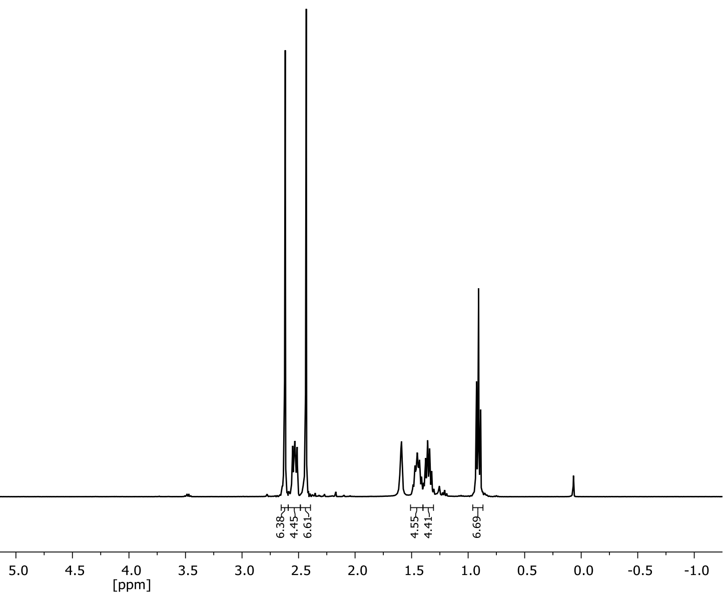
2.00
6.38
4.45
6.61
4.55
4.41
6.69
9.0 8.5 8.0 7.5 7.0 6.5 6.0 5.5 5.0 4.5 4.0 3.5 3.0 2.5 2.0 1.5 1.0 0.5 0.0 -0.5 -1.0
[ppm]

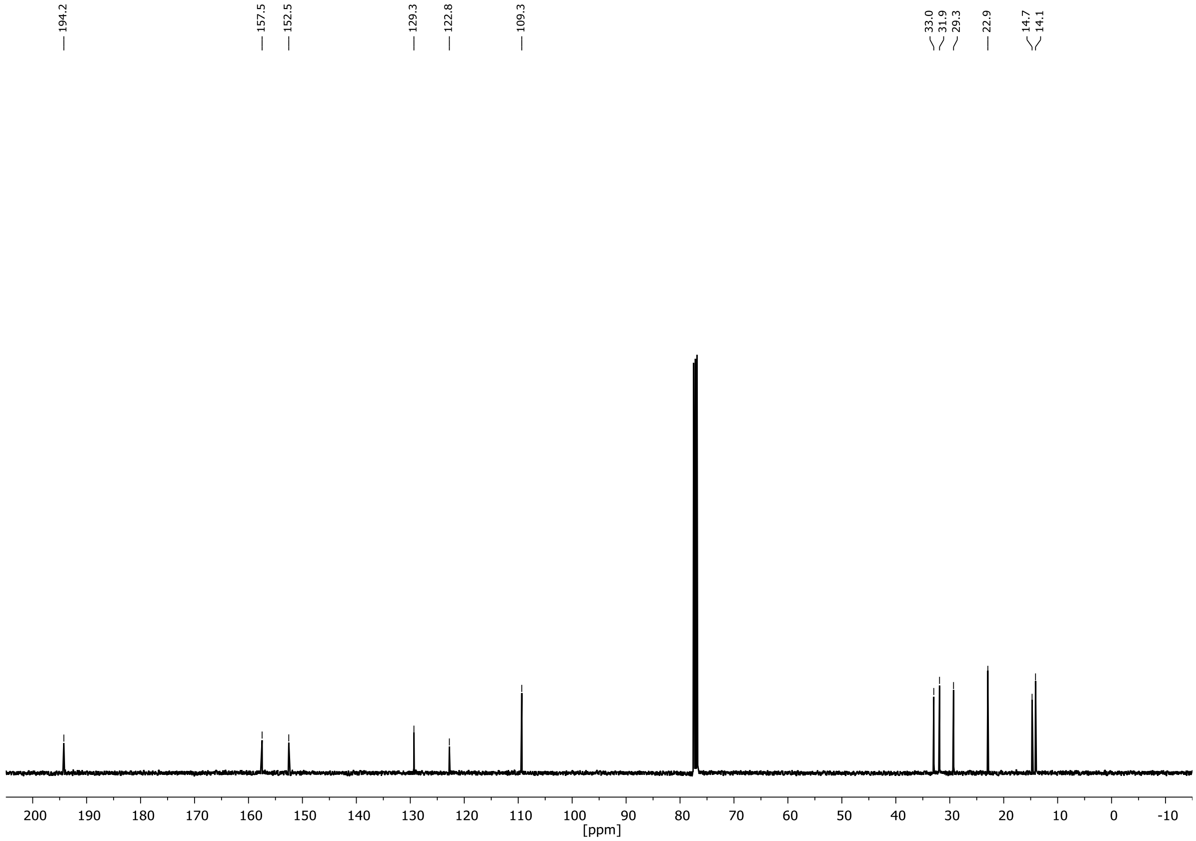
194.2
157.5
152.5
129.3
122.8
109.3
33.0
31.9
29.3
22.9
14.7
14.1
200 190 180 170 160 150 140 130 120 110 100 90 80 70 60 50 40 30 20 10 0 -10
[ppm]

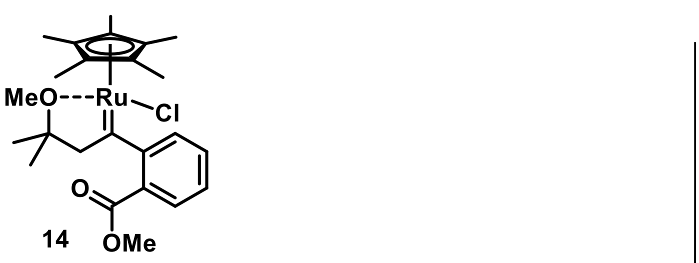
MeO
Ru
Cl
O
14
OMe

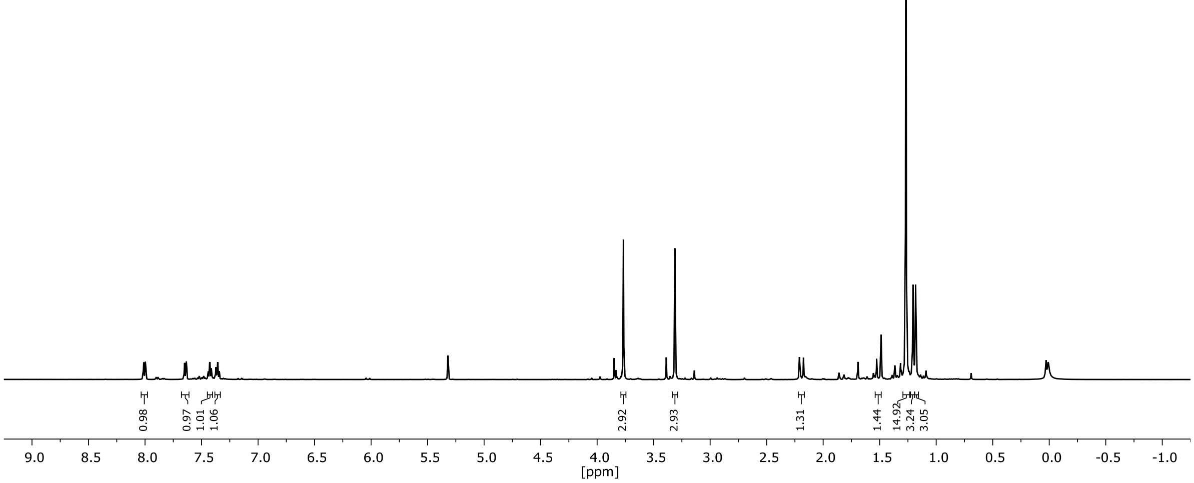
0.98
0.97
1.01
1.06
2.92
2.93
1.31
1.44
14.92
3.24
3.05
9.0 8.5 8.0 7.5 7.0 6.5 6.0 5.5 5.0 4.5 4.0 3.5 3.0 2.5 2.0 1.5 1.0 0.5 0.0 -0.5 -1.0
[ppm]

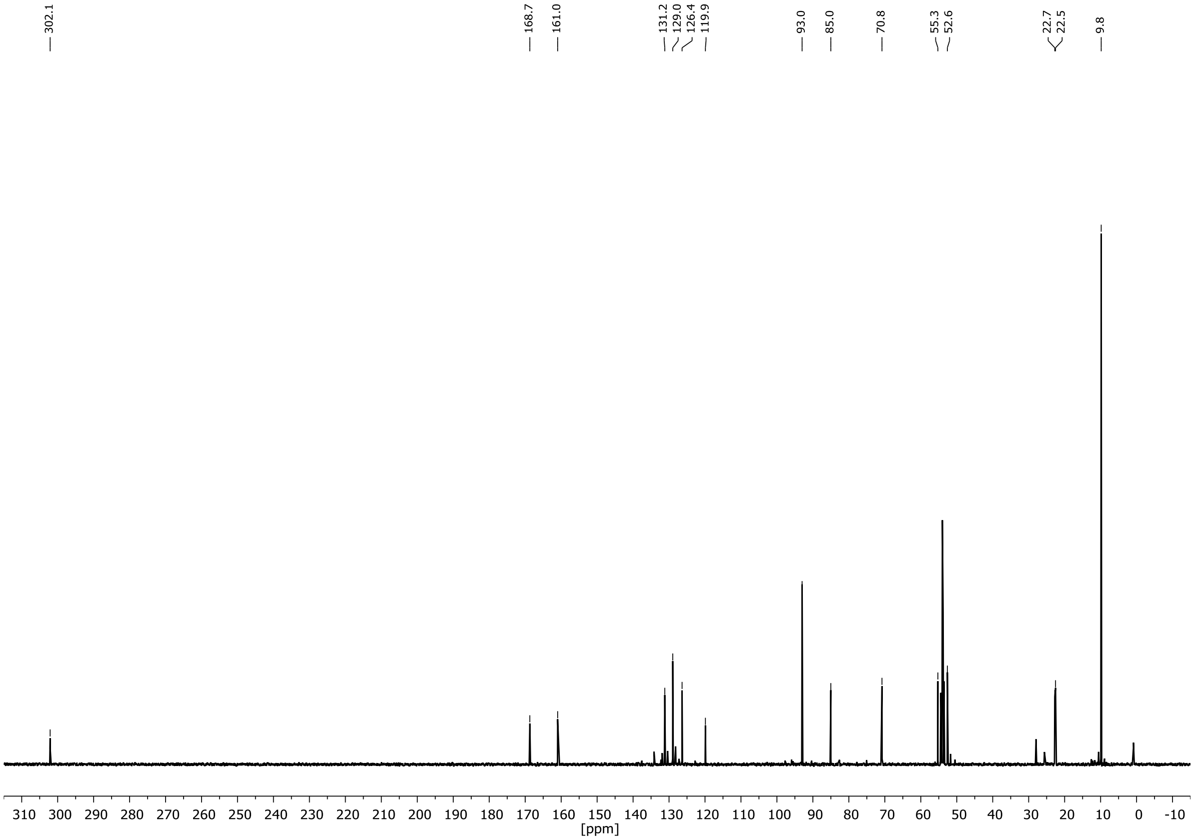
302.1
168.7
161.0
131.2
129.0
126.4
119.9
93.0
85.0
70.8
55.3
52.6
22.7
22.5
9.8
310 300 290 280 270 260 250 240 230 220 210 200 190 180 170 160 150 140 130 120 110 100 90 80 70 60 50 40 30 20 10 0 -10
[ppm]

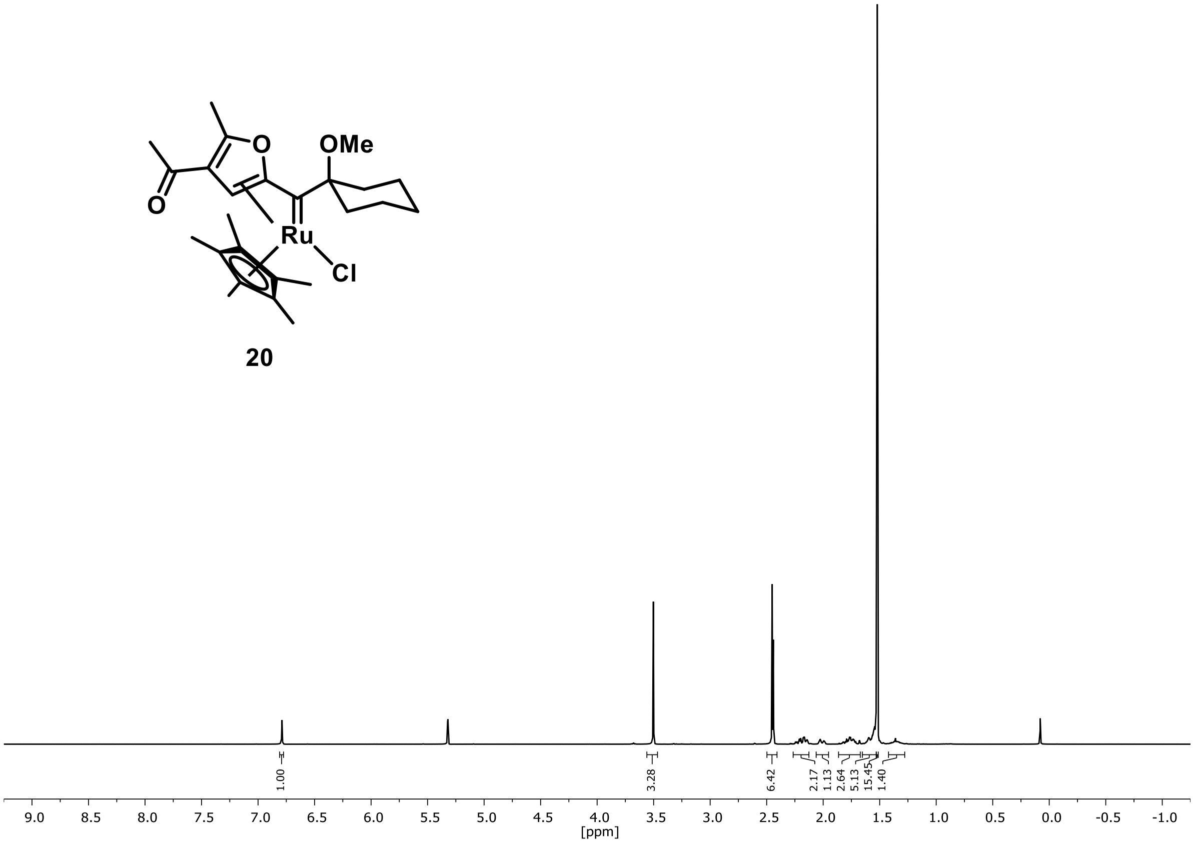
OMe
O
O
Ru
Cl
20
1.00
3.28
6.42
2.17
1.13
2.64
5.13
15.45
1.40
9.0 8.5 8.0 7.5 7.0 6.5 6.0 5.5 5.0 4.5 4.0 3.5 3.0 2.5 2.0 1.5 1.0 0.5 0.0 -0.5 -1.0
[ppm]

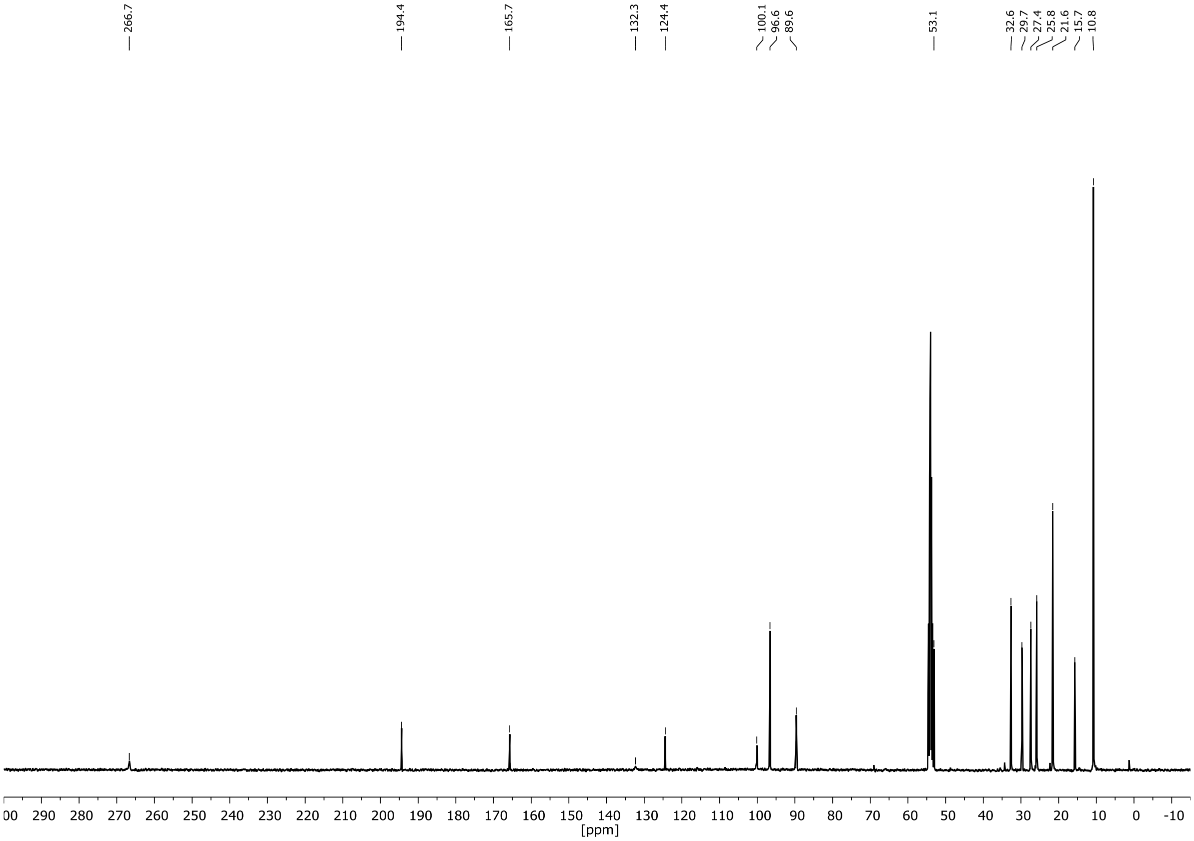
266.7
194.4
165.7
132.3
124.4
100.1
96.6
89.6
53.1
32.6
29.7
27.4
25.8
21.6
15.7
10.8
00 290 280 270 260 250 240 230 220 210 200 190 180 170 160 150 140 130 120 110 100 90 80 70 60 50 40 30 20 10 0 -10
[ppm]

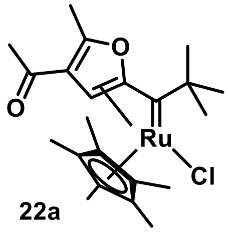
O
O
Ru
Cl

22a

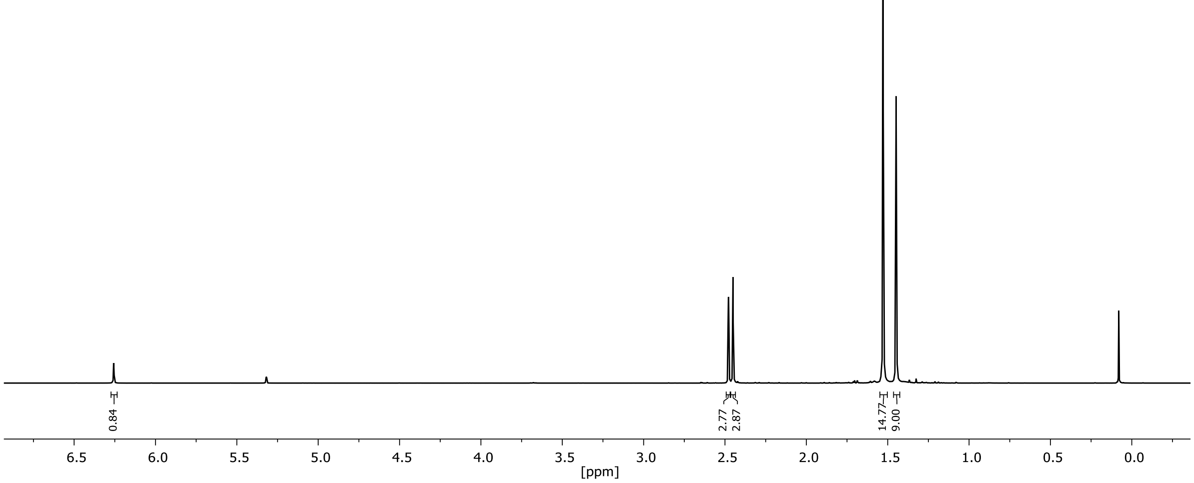
0.84
2.77
2.87
14.77
9.00
6.5
6.0
5.5
5.0
4.5
4.0
3.5
3.0
2.5
2.0
1.5
1.0
0.5
0.0
[ppm]

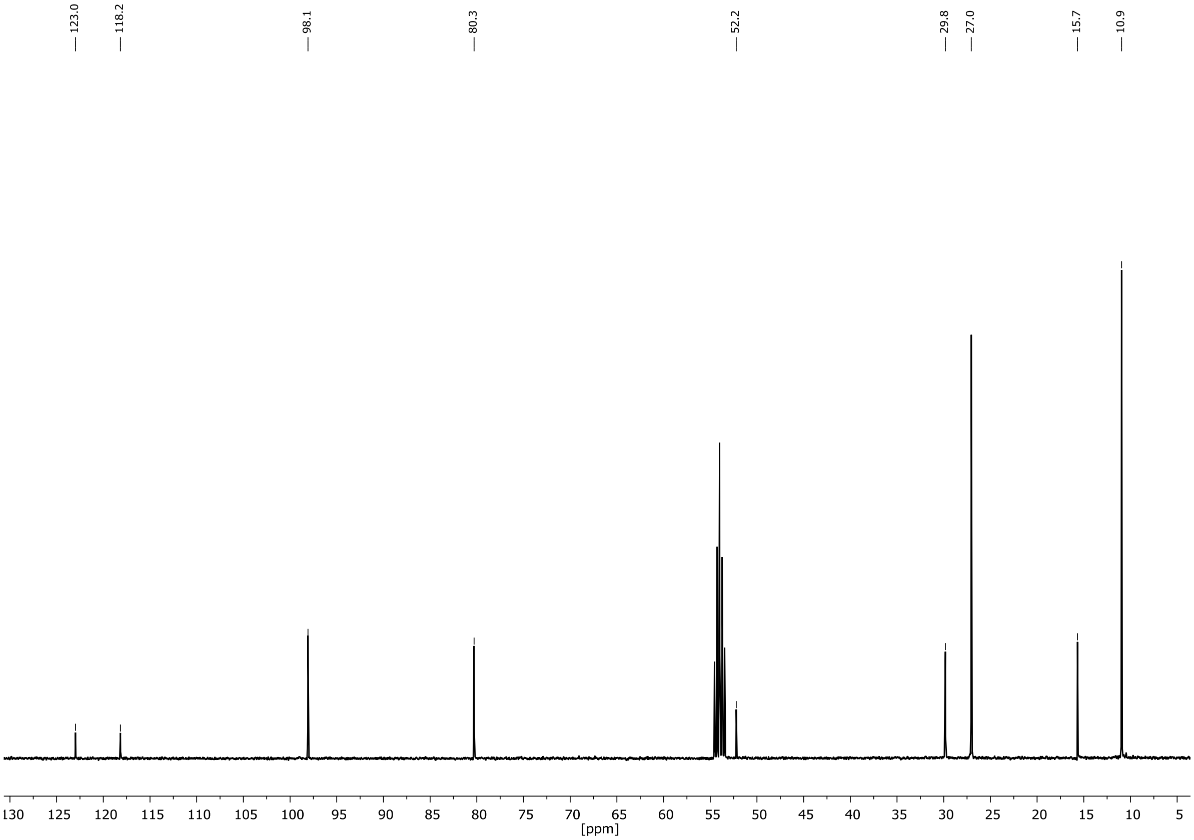
123.0
118.2
98.1
80.3
52.2
29.8
27.0
15.7
10.9
130
125
120
115
110
105
100
95
90
85
80
75
70
65
60
55
50
45
40
35
30
25
20
15
10
5
[ppm]

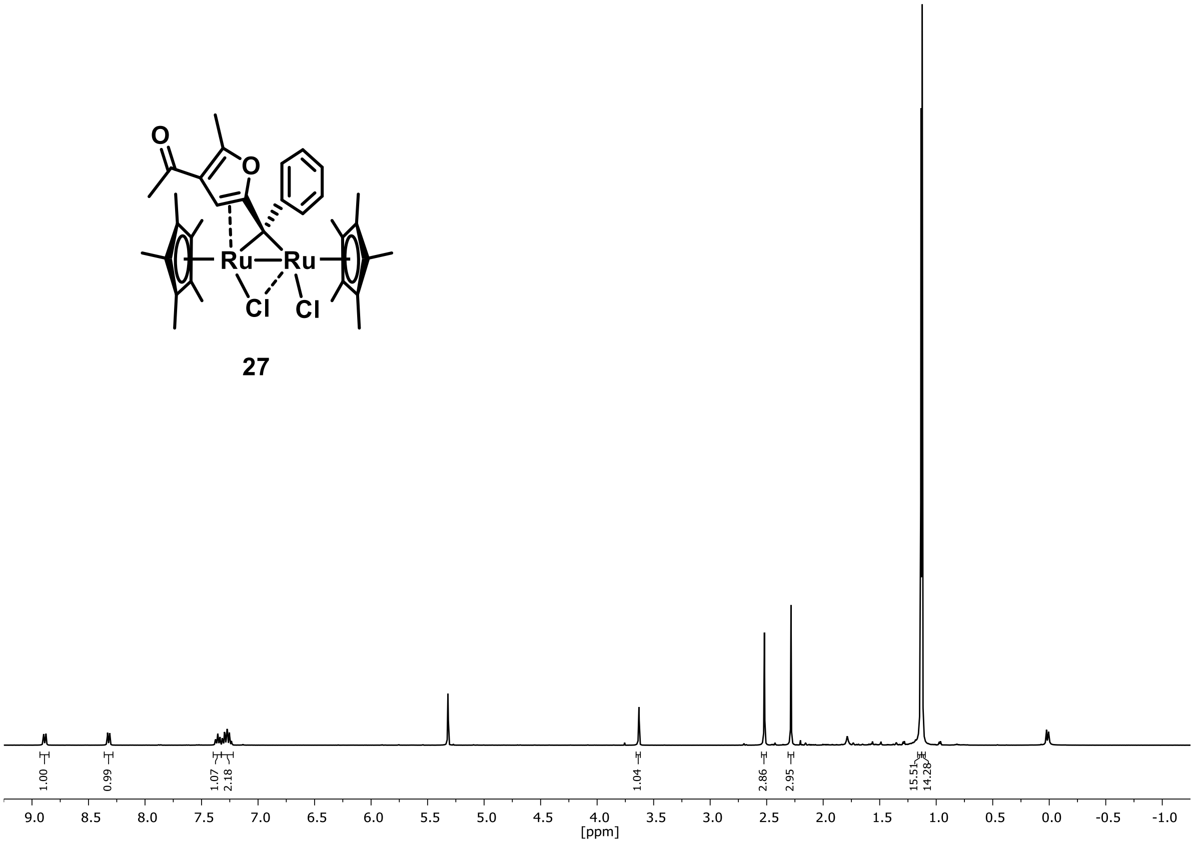
O
O
Ru
Ru
Cl
Cl
1.00
0.99
1.07
2.18
1.04
2.86
2.95
15.51
14.28
9.0 8.5 8.0 7.5 7.0 6.5 6.0 5.5 5.0 4.5 4.0 3.5 3.0 2.5 2.0 1.5 1.0 0.5 0.0 -0.5 -1.0
[ppm]

27

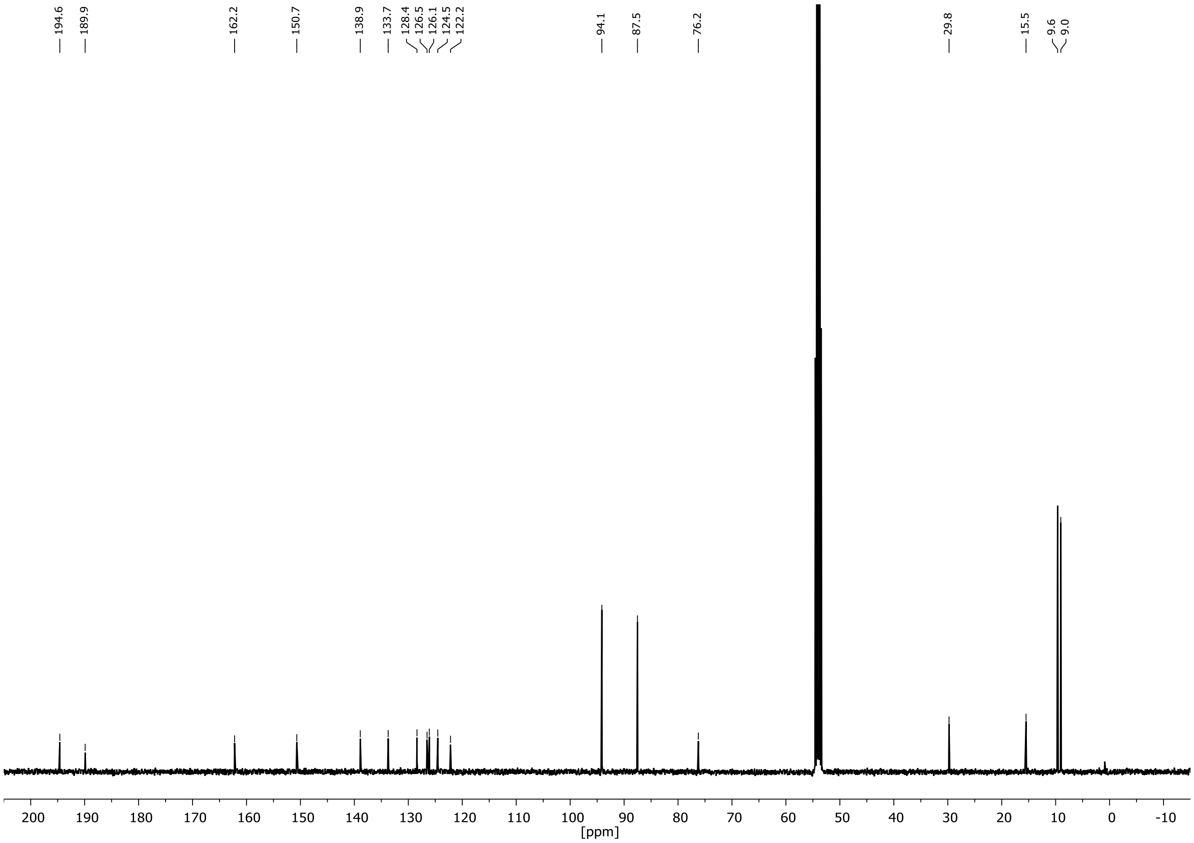
194.6
189.9
162.2
150.7
138.9
133.7
128.4
126.5
126.1
124.5
122.2
94.1
87.5
76.2
29.8
15.5
9.6
9.0
200 190 180 170 160 150 140 130 120 110 100 90 80 70 60 50 40 30 20 10 0 -10
[ppm]

REFERENCES

[1] a) J. A. Aguilar, R. W. Adams, S. B. Duckett, G. G. R. Green, R. Kandiah, *J. Magn. Reson.* **2011**, 208, 49–57; b) J. A. Aguilar, P. I. P. Elliott, J. López-Serrano, R. W. Adams, S. B. Duckett, *Chem. Commun.* **2007**, 1183 - 1185

[2] P. J. Fagan, W. S. Mahoney, J. S. Calabrese, J. C. Williams, *Organometallics* **1990**, 9, 1843 – 1852

[3] T. Biberger, C. P. Gordon, M. Leutzsch, S. Peil, A. Guthertz, C. Copéret, A. Fürstner, *Angew. Chem. Int. Ed.* **2019**, 58, 8845 – 8850

[4] C. Kong, T. G. Driver, *Org. Lett.* **2015**, 17, 802 - 805

[5] K. Ohe, K. Miki, T. Yokoi, F. Nishino, S. Uemura, *Organometallics* **2000**, 19, 5525 – 5528

[6] B. Scheiper, M. Bonnekessel, H. Krause, A. Fürstner, *J. Org. Chem.* **2004**, 69, 3943 - 3949

[7] C. E. Wagner, K. J. Shea, *Org. Lett.* **2004**, 6, 313 - 316

[8] A. Padwa, K. E. Krumpe, J. M. Kassir, *J. Org. Chem.* **1992**, 57, 4940 - 4948

[9] N. Su, J. A. Theorell, D. J. Wink, T. G. Driver, *Angew. Chem. Int. Ed* **2015**, 54, 12942 – 12946

[10] S. Bhunia, S. Ghorpade, D. B. Huple, R.-S. Liu, *Angew. Chem. Int. Ed* **2012**, 51, 2939 - 2942

[11] A. Weber, R. Dehn, N. Schläger, B. Dieter, A. Kirschning, *Org. Lett.* **2014**, 16, 568 - 571

[12] Y. Zhou, J. Ma, K. Chen, H. Jiang, S. Zhu, *Chem. Commun.* **2016**, 52, 13345 – 13348
